# Selective Synthesis of Cyclooctanoids by Radical Cyclization of Seven‐Membered Lactones: Neutron Diffraction Study of the Stereoselective Deuteration of a Chiral Organosamarium Intermediate

**DOI:** 10.1002/anie.201606792

**Published:** 2016-09-07

**Authors:** Xavier Just‐Baringo, Jemma Clark, Matthias J. Gutmann, David J. Procter

**Affiliations:** ^1^ School of ChemistryUniversity of ManchesterManchesterM13 9PLUK; ^2^ ISIS FacilityRutherford Appleton LaboratoryChilton, DidcotOxfordshireOX11 0QXUK

**Keywords:** cyclization, cyclooctanes, neutron diffraction, radicals, samarium diiodide

## Abstract

Seven‐membered lactones undergo selective SmI_2_–H_2_O‐promoted radical cyclization to form substituted cyclooctanols. The products arise from an exo‐mode of cyclization rather than the usual endo‐attack employed in the few radical syntheses of cyclooctanes. The process is terminated by the quenching of a chiral benzylic samarium. A labeling experiment and neutron diffraction study have been used for the first time to probe the configuration and highly diastereoselective deuteration of a chiral organosamarium intermediate.

Mapping new routes to challenging molecular architectures is a major driving force in the development of synthetic chemistry.^1^ Cyclooctanes are found in important natural products and pharmaceuticals and present a fascinating synthetic challenge due to their high ring strain and transannular interactions.^2^, ^3^ The race towards the total synthesis of paclitaxel (Taxol)^4^ spurred particular interest in the motif and the construction of cyclooctanes has become a fertile field (Scheme 1).^5^, ^6^, ^7^, ^8^, ^9^, ^10^, ^11^ Among these methods, radical cyclization approaches are scarce and have relied mainly on radicals generated from halides,^12^ ketones^13^, ^14^ and aldehydes^15^ and 8‐*endo* cyclization modes (Scheme 2 A).^16^

**Scheme 1:**
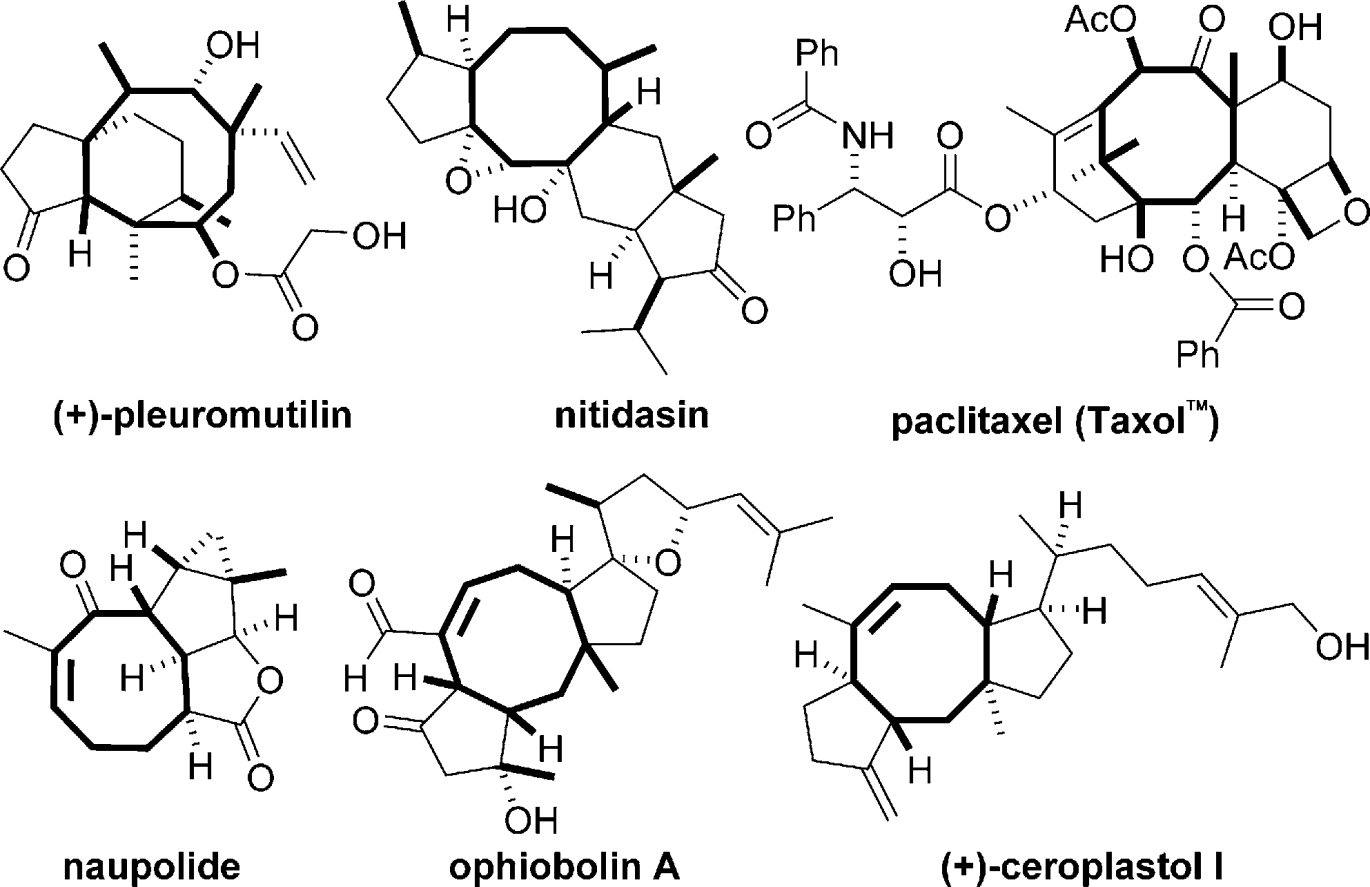
Natural products containing cyclooctane rings.

**Scheme 2:**
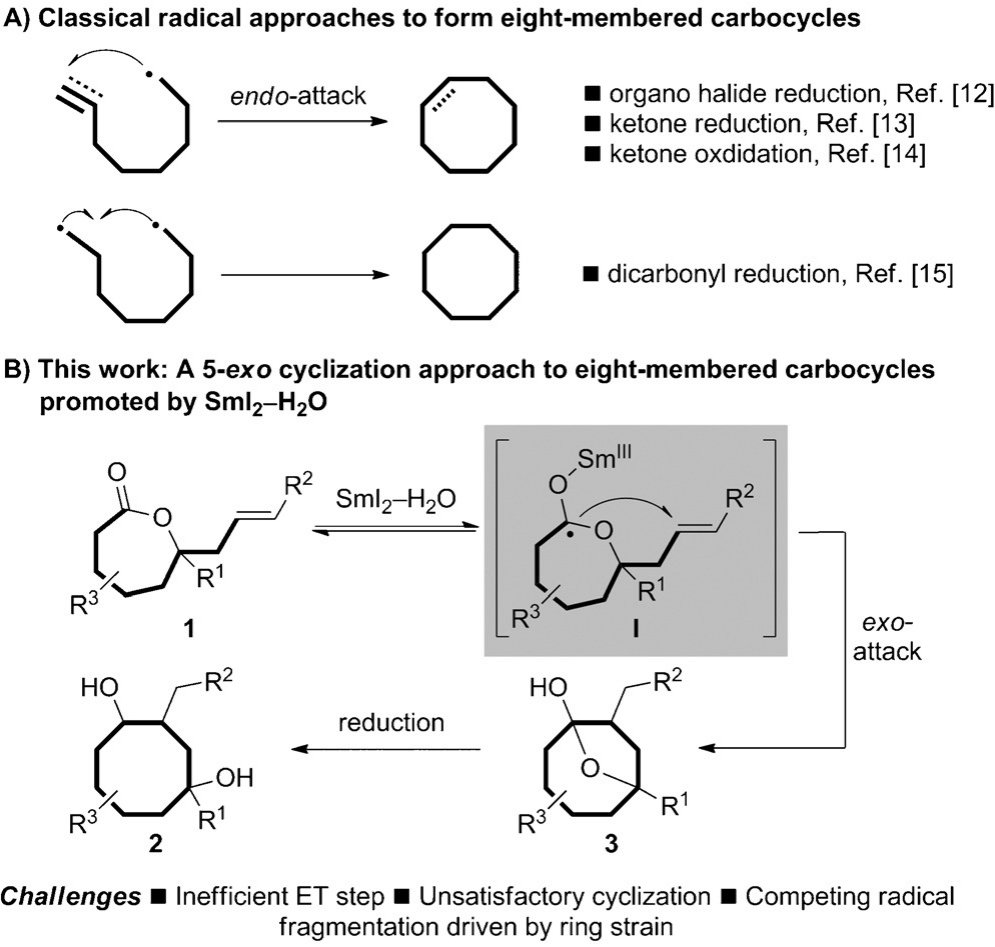
A) 8‐*Endo* cyclizations dominate cyclooctane synthesis under radical conditions. B) A proposed 5‐*exo* radical cyclization approach to cyclooctanes and the challenges involved.

Samarium diiodide (Kagan's reagent, SmI_2_)^17^, ^18^ is perhaps the most versatile reductive electron transfer (ET) reagent and has been used extensively for carbon–carbon bond forming reactions.^19^ We recently expanded the scope of SmI_2_‐mediated reactions by introducing activation modes involving the reduction of carboxylic acid derivatives using SmI_2_–H_2_O.^20^ The acyl‐type radicals now accessible have been exploited in new functional group transformations and highly selective radical cyclizations involving carbon–carbon bond formation.^20^, ^21^, ^22^, ^23^, ^24^ In the case of lactones, ET from Sm^II^ to the carbonyl gives rise to radical anions (cf. **I** in Scheme 2) that are stabilized by hyperconjugation with the adjacent oxygens and by H_2_O.^20^, 21a–21e Very recently, we demonstrated that ET to *all* lactones using SmI_2_–H_2_O is reversible and this back ET typically impedes productive reductive transformations.21d However, new opportunities for carbon–carbon bond formation arise if the transient radical anions can be trapped by a suitably placed radical acceptor.

Here we describe a synthesis of substituted cyclooctanes that exploits the first radical cyclizations of seven‐membered lactones, which can be easily accessed by Baeyer–Villiger oxidation of cyclohexanones. The process involves ET to the lactone **1**, and generation of radical anion **I**, followed by trapping of the radical by the tethered alkene. Crucially, potential issues involving back ET to Sm^III^, radical fragmentation, and radical reduction are overcome. In situ reduction of the hemiketal intermediate **3** delivers 1,4‐cyclooctandiols **2** (Scheme 2 B). The 5‐*exo*‐trig radical cyclization of lactones stands in sharp contrast to most radical approaches to cyclooctanes that involve 8‐*endo* attack (Scheme 2 B).3b Furthermore, we report the use of a labeling experiment and a neutron diffraction study to probe for the first time the configuration and highly diastereoselective quenching of a chiral organosamarium.

The feasibility of the transformation was first assessed using lactone **1 a** (R^1^=Me; R^2^=Ph; R^3^=H) (Table 1). As expected, no reaction was observed when **1 a** was treated with SmI_2_ in THF (2‐fold excess) and only upon addition of H_2_O was conversion observed. After optimization of the amount of H_2_O additive employed, 1,4‐cyclooctandiol **2 a** was obtained in good isolated yield. Oxidation of the crude diol **2 a** with Dess–Martin periodinane facilitated assessment of the diastereoselectivity and stereochemical course of the radical cyclization by simplifying the diastereoisomeric mixture and providing crystalline product **3 a** in 74 % overall, isolated yield. 7‐Methyl substituted lactones **1 b**–**h** (R^1^=Me), bearing aryl‐substituted alkene tethers with various groups in all positions of the aromatic moiety, underwent efficient cyclization to give the corresponding hemiketals **3 b**–**h** in good to excellent yields (62–93 %, 2 steps) and with good diastereoselectivities (75:25 to 89:11 d.r.). Variation of the substituent in position 7 of the lactone proved possible. For example, benzyl substituted hemiketals **3 l**,**m** (R^1^=Bn) were obtained in good to excellent isolated yield and with good diastereocontrol. Halogen substituents were compatible with the cyclization conditions (formation of **3 b**, **3 c**, **3 d**, **3 e**, **3 m**, **2 o**) and serve as handles for further functionalization of the products. The trifluoromethyl group also proved stable to the reducing conditions and cyclooctane **3 c** was obtained in excellent overall isolated yield. X‐ray crystallographic analysis of **3 a**–**d** revealed the *syn* selectivity of the cyclization.^25^ Terminal alkenes could also be employed to intercept the radical anion intermediate, however, in the absence of an aryl substituent on the alkene, radical cyclization was less efficient and cyclooctanes **3 i** and **3 k** were obtained in low overall yield and with lower diastereoselectivity. The cyclization proved surprisingly tolerant of steric hindrance: lactone **1 j**, bearing *gem*‐disubstitution α to the lactone carbonyl, underwent efficient radical cyclization upon treatment with SmI_2_–H_2_O. In this particular case, the product obtained in good overall yield after oxidation was the hydroxyketone **3 j** rather than the corresponding hemiketal.

**Table 1:** Cyclooctanoid synthesis by 5‐*exo*‐trig radical cyclization of seven‐membered lactones with SmI_2_–H_2_O.^[a]^ 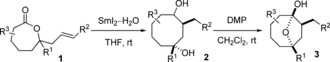

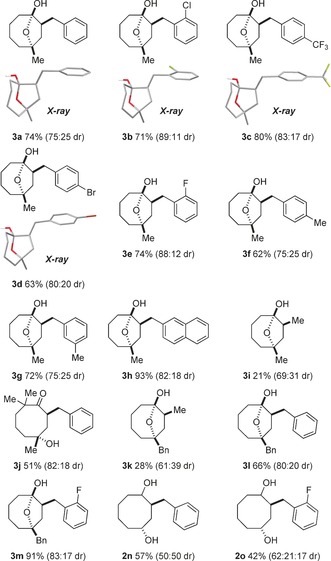

[a] Conditions: SmI_2_ (8 equiv, 2‐fold excess), THF, H_2_O (800 equiv), room temperature. Isolated yields for 2 steps. Diastereoselectivities were determined from ^1^H NMR spectra of crude product mixtures.

Lactones **1 n** and **1 o** lacking an alkyl substituent at the 7 position of the ring (R^1^=H) also underwent cyclization to give 1,4‐cyclooctandiols **2 n** and **2 o** in moderate yield. This observation likely results from the lower relative stability of the required reactive conformation in which the alkene tether adopts a pseudo‐axial conformation (Scheme 3). The absence of an alkyl substituent in position 7 of the lactone ring favors a pseudo‐equatorial conformation of the tether, disfavoring its interaction with the radical anion.

**Scheme 3:**
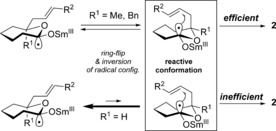
The impact of lactone conformation on the efficiency of radical cyclization.

The relative configuration of the products is consistent with an *anti* attack of the radical anion intermediate on the tethered alkene (conformation **IIb**) involving a product‐like transition state, followed by a second ET and subsequent protonation (or deuteration, see below) (Scheme 4). The observed selectivity likely results from minimization of electrostatic interactions and steric clashes between the alkene and the radical anion intermediate, thus favoring **IIb** over **IIa**.^26^ Subsequent ET to **III** and protonation of **IV** gives rise to hemiketal **3 a**, which is then reduced further.

**Scheme 4:**
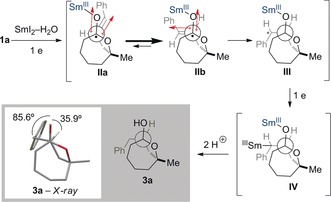
Proposed mechanism and rationale for the origin of diastereoselectivity in the radical cyclization.

Interestingly, carrying out the cyclization of **1 a** with SmI_2_–D_2_O gave ***d*** **‐3 a** with high diastereoselectivity at the benzylic position (>90:10 d.r.). To gain further insight into the mechanism of the radical cyclization and the nature of the organosamarium intermediate **IV**,^27^ formed upon reduction of radical **III**, we determined the relative configuration of the deuterated product ***d*** **‐3 a** using neutron diffraction (Scheme 5).^28^ Based on the analysis of a crystalline sample of ***d*** **‐3 a**, we propose that a chelated, chiral organosamarium intermediate **IV** is formed^29^ and the more stable ***anti*** **‐IV** diastereomer is quenched selectively with retention of configuration at carbon to generate samarium alkoxide **V**. Finally, deuteration of samarium(III) alkoxide **V** delivers ***d*** **‐3 a**.^30^ To our knowledge this is the first time that the configuration of a diastereoselectively deuterated product has been confirmed using neutron diffraction.^31^

**Scheme 5:**
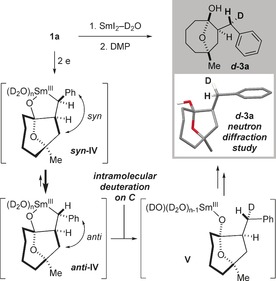
Proposed stereoretentive quenching of a chiral organosamarium: neutron scattering studies.

In conclusion, radical *exo*‐cyclization of unsaturated seven‐membered lactones, triggered by single ET to the carbonyl group by SmI_2_–H_2_O, generates cyclooctanes typically in good yield and high diastereoselectivity. Neutron diffraction has been used to probe, for the first time, the stereochemical course of the selective deuteration of a chiral organosamarium intermediate.

## Supporting information

As a service to our authors and readers, this journal provides supporting information supplied by the authors. Such materials are peer reviewed and may be re‐organized for online delivery, but are not copy‐edited or typeset. Technical support issues arising from supporting information (other than missing files) should be addressed to the authors.

Supplementary
