# Supplementary material for: Selective Synthesis of Cyclooctanoids by Radical Cyclization of Seven‐Membered Lactones: Neutron Diffraction Study of the Stereoselective Deuteration of a Chiral Organosamarium Intermediate
Source: Angew Chem Int Ed Engl. 2016 Sep 7;55(40):12499–502. doi: 10.1002/anie.201606792 (PMC5113801; doi:10.1002/anie.201606792)

Angewandte Chemie
Eine Zeitschrift der Gesellschaft Deutscher Chemiker

Supporting Information

# Selective Synthesis of Cyclooctanoids by Radical Cyclization of Seven-Membered Lactones: Neutron Diffraction Study of the Stereoselective Deuteration of a Chiral Organosamarium Intermediate

*Xavier Just-Baringo, Jemma Clark, Matthias J. Gutmann, and David J. Procter**

anie_201606792_sm_miscellaneous_information.pdf

## Table of contents

1. General Information…………………………………………………………….... S1
2. Control experiment………………………………………………………………S2
3. Experimental procedures………………………………………………………... S2
4. X-ray structure of **3a**……………………………………………………………… S42
5. X-ray structure of **3b**……………………………………………………….....….. S44
6. X-ray structure of **3c**……………………………………………………………….. S46
7. X-ray structure of **3d**……………………………………………………....……….. S48
8. Neutron diffraction studies on ***d*-3a**………………………………………........... S50
9. NMR spectra…………………………………………………………………….. S52
10. $D_2O$ experiment…………………………………………….....……………….. S94

## 1. General Information

THF, triethylamine and $CH_2Cl_2$ were freshly distilled before use. THF was distilled over sodium wire and benzophenone; $CH_2Cl_2$ and triethylamine were distilled over calcium hydride. All other solvents and reagents used were purchased from commercial suppliers and used according to relevant guidelines. $^{1}$H-NMR spectra were obtained at room temperature on a Bruker 400 MHz or 500 MHz spectrometer. $^{13}$C-NMR were obtained at 101 or 126 MHz respectively. $^{19}$F-NMR were obtained at 376 MHz. All NMR spectra were processed using *ACDLabs*© NMR software. Chemical shifts are reported in parts per million (ppm) and coupling constants (*J*) reported in Hz. Splitting patterns are reported as follows: singlet (s), doublet (d), triplet (t), doublet of doublets of triplets (ddt), doublet of doublets of doublets (ddd) and multiplet (m). In $^{13}$C NMR, quaternary carbons that cannot be unambiguously assigned are labeled as *C*Q. Information regarding peak shape and intensity for IR data is as follows: weak (w), medium (m), strong (s) and broad (b) and values are reported in $cm^{-1}$. TLC analysis was carried out on aluminium sheets coated with silica gel and visualised using potassium permanganate solution and/or UV light.

## 2. Control reaction

The requirement of $H_2O$ as an additive in the $SmI_2$-promoted lactone cyclization was assessed by treating lactone **1a** with $SmI_2$ alone. As expected, no cyclization took place and starting material was recovered unchanged.

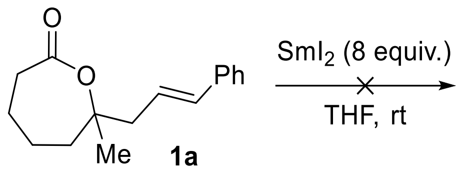


## 3. Experimental procedures

**General Procedure A: Dieckmann condensation and alkylation**

**Allyl 1-methyl-2-oxocyclohexane-1-carboxylate (S1)**

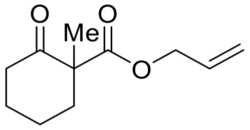


A solution of diallyl pimelate[1] (20.0 g, 78.2 mmol) in THF (13 mL) was added slowly to a stirred suspension of 60% NaH in mineral oil (3.44 g, 86 mmol) in THF (65 mL). The resulting mixture was stirred at 40 ºC. After 18 h, methyl iodide (6.3 mL, 102 mmol) was added and the mixture was stirred at 40 ºC for 3 h. The mixture was then allowed to cool down to room temperature before carefully quenching the the reaction by slow addition of $H_2O$ (16 mL). Most of the volatiles were removed under vacuum and the remaining solution was extracted with EtOAc (4 × 20 mL). The combined organics were washed with brine (15 mL), dried ($MgSO_4$) and concentrated. The crude product was used in the next step without further purification.

[1] Prepared according to the procedure reported in: Mohr, J. T.; Krout, M. R.; Stoltz, B. M. *Org. Synth.* **2009**, *86*, 194.

**General procedure B: decarboxylative allylation**

**2-Allyl-2-methylcyclohexan-1-one (S2)**[2]

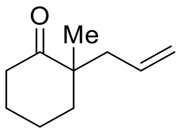


To a solution of crude ketoester **S1** (78.2 mmol) in THF (700 mL) were added $Pd_2(dba)_3$ (3.58 g, 3.91 mmol) and triphenylphosphine (4.10 g, 15.6 mmol) in one portion. The resulting solution was stirred at reflux for 14 h before allowing it to cool down to room temperature. Solvent was removed under vacuum and the remaining residue was filtered through a short pad of silica with $Et_2O$ as the eluent. The product was distilled from the crude mixture (97−100 ºC, 2 mmbar) and was obtained as a colourless oil (7.75 g, 50.9 mmol, 65% from pimelic acid). $^1$H-NMR (400 MHz, $CDCl_3$) δ 1.08 (s, 3 H, $CH_3$), 1.54–1.64 (m, 1 H, $CH_aH_bCH_2CH_2CH_2C(O)$), 1.65–1.93 (m, 5 H, $CH_aH_bCH_2CH_2CH_2C(O)$ + $CH_2CH_2CH_2CH_2C(O)$ + $CH_2CH_2CH_2CH_2C(O)$), 2.24 (dd, $J$ = 13.8, 7.2 Hz, 1 H, $CH_aH_bCH{=}CH_2$), 2.32–2.44 (m, 3 H, $CH_2CH_2CH_2CH_2C(O)$ + $CH_aH_bCH{=}CH_2$), 5.01–5.09 (m, 2 H, $CH_2CH{=}CH_2$), 5.64–5.76 (m, 1 H, $CH_2CH{=}CH_2$) ppm; $^{13}$C-NMR (100 MHz, $CDCl_3$) δ 21.0 ($CH_2CH_2CH_2CH_2C(O)$), 22.6 ($CH_3$), 27.4 ($CH_2CH_2CH_2CH_2C(O)$), 38.6 ($CH_2CH_2CH_2CH_2C(O)$), 38.8 ($CH_2CH_2CH_2CH_2C(O)$), 41.9 ($CH_2CH{=}CH_2$), 48.4 ($CH_3C$), 117.9 ($CH_2CH{=}CH_2$), 133.8 ($CH_2CH{=}CH_2$), 215.4 ($C$(O)) ppm; IR $\nu_{max}$ (thin film/cm$^{-1}$): 2932, 2864, 1705 (C=O), 1451, 1124, 994, 913; HRMS calcd for $C_{10}H_{16}ONa$, $[M+Na]^+$: 175.1099, found 175.1104.

**General Procedure C: Baeyer-Villiger Oxidation**

**7-Allyl-7-methyloxepan-2-one (1i)**

[2] The product matched the data reported in ref. 1.

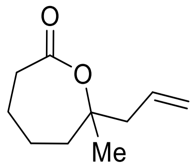


$NaHCO_3$ (1.10 g, 13.1 mmol) was added to a solution of ketone **S2** (1.00 g, 6.57 mmol) in $CH_2Cl_2$ (14.6 mL). The resulting suspension was placed in an ice bath and *m*CPBA (1.62 g, 7.23 mmol) was added in portions. The mixture was subsequently stirred for 17 h while allowing it to reach room temperature. The mixture was partitioned between $CH_2Cl_2$ (25 mL) and saturated aqueous $NaHCO_3$ (60 mL). Layers were separated and the aqueous layer was extracted with $CH_2Cl_2$ (2 × 25 mL). The combined organic fractions were dried ($MgSO_4$) and concentrated under reduced pressure. The resulting crude mixture was purified by silica gel column chromatography (hexane/EtOAc, 90:10 to 85:15). The title product was obtained as a colourless oil (669 mg, 3.98 mmol, 61%). $^1$H-NMR (400 MHz, $CDCl_3$) δ 1.44 (s, 3 H, $CH_3$), 1.61-1.72 (m, 1 H, $CH_2CH_2CH_aH_bCH_2C(O)O$), 1.73-1.90 (m, 5 H, $CH_2CH_2CH_aH_bCH_2C(O)O$ + $CH_2CH_2CH_2CH_2C(O)O$ + $CH_2CH_2CH_2CH_2C(O)O$), 2.44 (dd, $J$ = 14.0, 7.6 Hz, 1 H, $CH_aH_bCH{=}CH_2$), 2.52 (dd, $J$ = 14.0, 7.0 Hz, 1 H, $CH_aH_bCH{=}CH_2$), 2.63-2.79 (m, 2 H, $CH_2CH_2CH_2CH_2C(O)O$), 5.08-5.19 (m, 2 H, $CH_2CH{=}CH_2$), 5.87 (m, 1 H, $CH_2CH{=}CH_2$) ppm; $^{13}$C-NMR (100 MHz, $CDCl_3$) δ 23.4 ($CH_2CH_2CH_2CH_2C(O)O$), 23.9 ($CH_2CH_2CH_2CH_2C(O)O$), 24.8 ($CH_3$), 37.4 ($CH_2CH_2CH_2CH_2C(O)O$), 38.4 ($CH_2CH_2CH_2CH_2C(O)O$), 46.7 ($CH_2CH{=}CH_2$), 82.8 ($CH_3CO$), 119.0 ($CH_2CH{=}CH_2$), 132.8 ($CH_2CH{=}CH_2$), 174.8 ($C(O)O$) ppm; IR $\nu_{max}$ (thin film/cm$^{-1}$): 2935, 1711 (C=O), 1145, 1285, 1170, 1017; HRMS calcd for $C_{10}H_{16}O_2Na$, $[M+Na]^+$: 191.1048, found 191.1048.

**General Procedure D: Cross-Metathesis**

**7-Cinnamyl-7-methyloxepan-2-one (1a)**

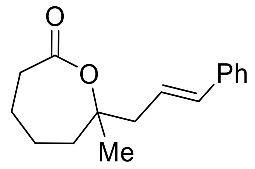

To a solution of lactone **1i** (200 mg, 1.19 mmol) and styrene (410 μL, 3.57 mmol) in degassed $CH_2Cl_2$ (3.0 mL) was added Hoveyda-Grubbs 2nd generation catalyst (7 mg, 11.9 μmol). The resulting solution was stirred at room temperature under a very slow stream of nitrogen for 23 h. Solvent was removed under vacuum and the resulting crude mixture was purified by silica gel column chromatography (hexane/EtOAc, 100:0 to 80:20). The title product was obtained as a pale yellow oil (112 mg, 0.458 mmol, 39%). $^1$H-NMR (400 MHz, $CDCl_3$) δ 1.50 (s, 3 H, $CH_3$), 1.62-1.73 (m, 1 H, $CH_2CH_2CH_aH_bCH_2C(O)O$), 1.75-1.94 (m, 5 H, $CH_2CH_2CH_aH_bCH_2C(O)O$ + $CH_2CH_2CH_2CH_2C(O)O$ + $CH_2CH_2CH_2CH_2C(O)O$), 2.56-2.83 (m, 4 H, $CH_2CH$=CHPh + $CH_2CH_2CH_2CH_2C(O)O$), 6.28 (dt, *J* = 15.8, 7.2 Hz, 1 H, $CH_2CH$=CHPh), 6.47 (d, *J* = 15.8 Hz, 1 H, $CH_2CH$=C*H*Ph), 7.23 (tt, *J* = 7.2, 1.6 Hz, 1 H, Ar*H*), 7.29-7.35 (m, 2 H, Ar*H*), 7.36-7.41 (m, 2 H, Ar*H*) ppm; $^{13}$C-NMR (100 MHz, $CDCl_3$) δ 23.4 ($CH_2CH_2CH_2CH_2C(O)O$), 24.0 ($CH_2CH_2CH_2CH_2C(O)O$), 24.7 ($CH_3$), 37.4 ($CH_2CH_2CH_2CH_2C(O)O$), 38.6 ($CH_2CH_2CH_2CH_2C(O)O$), 46.4 ($CH_2CH$=CHPh), 83.2 ($CH_3C$), 124.4 ($CH_2CH$=CHPh), 126.2 (Ar-*C*H), 127.4 (Ar-*C*H), 128.6 (Ar-*C*H), 134.0 (CH=*C*HPh), 137.1 (Ar-*C*), 174.8 (*C*(O)O) ppm; IR $\nu_{max}$ (thin film/cm$^{-1}$): 2933, 1710 (C=O), 1448, 1286, 1176, 1017, 969; HRMS calcd for $C_{16}H_{24}O_2N$ $[M+NH_4]^+$: 262.1802, found 262.1806.

**(*E*)-7-(3-(2-Chlorophenyl)allyl)-7-methyloxepan-2-one (1b)**

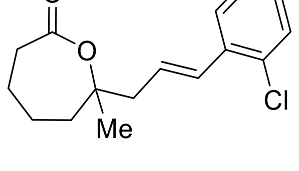

Prepared according to general procedure D using lactone **1i** (100 mg, 0.594 mmol), 2-chlorostyrene (230 μL, 1.78 mmol) and Hoveyda-Grubbs 2nd generation catalyst (4 mg, 5.94 μmol) in $CH_2Cl_2$ (1.5 mL). The crude mixture was purified by silica gel column chromatography (hexane/EtOAc, 100:0 to 80:20). The title product was obtained as a pale yellow oil (109 mg, 0.391 mmol, 66%). $^1$H-NMR (400 MHz, $CDCl_3$) δ 1.51 (s, 3 H, $CH_3$), 1.64-1.73 (m, 1 H, $CH_2CH_2C\mathit{H}_aH_bCH_2C(O)O$), 1.76-1.95 (m, 5 H, $CH_2CH_2CH_a\mathit{H}_bCH_2C(O)O$ + $CH_2C\mathit{H}_2CH_2CH_2C(O)O$ + $C\mathit{H}_2CH_2CH_2CH_2C(O)O$), 2.58-2.84 (4 H, $C\mathit{H}_2CH{=}CHAr$ + $CH_2CH_2CH_2C\mathit{H}_2C(O)O$), 6.28 (dt, *J* = 15.8, 7.6 Hz, 1 H, $CH_2C\mathit{H}{=}CHAr$), 6.84 (d, *J* = 15.8 Hz, 1 H, $CH_2CH{=}C\mathit{H}Ar$), 7.17 (dt, *J* = 7.6, 1.6 Hz, 1 H, Ar*H*), 7.22 (dt, *J* = 7.6, 1.6 Hz, 1 H, Ar*H*), 7.35 (dt, *J* = 7.6, 1.6 Hz, 1 H, Ar*H*), 7.55 (dt, *J* = 7.6, 1.6 Hz, 1 H, Ar*H*) ppm; $^{13}$C-NMR (100 MHz, $CDCl_3$) δ 23.4 ($CH_2CH_2\mathit{C}H_2CH_2C(O)O$), 24.0 ($CH_2\mathit{C}H_2CH_2CH_2C(O)O$), 24.7 ($\mathit{C}H_3$), 37.4 ($CH_2CH_2CH_2\mathit{C}H_2C(O)O$), 38.7 ($\mathit{C}H_2CH_2CH_2CH_2C(O)O$), 46.5 ($\mathit{C}H_2CH{=}CHAr$), 83.0 ($CH_3\mathit{C}O$), 126.8 (Ar*C*H), 126.9 (Ar*C*H), 127.5 ($CH_2\mathit{C}H{=}CHAr$), 128.4 (Ar*C*H), 129.6 (Ar*C*H), 130.1 ($CH_2CH{=}\mathit{C}HAr$), 132.6 (Ar*C*), 135.2 (Ar*C*), 174.8 (*C*(O)O) ppm; IR $\nu_{max}$ (thin film/cm$^{-1}$): 2934, 1711 (C=O), 1441, 1290, 1177, 1101, 1017, 969; HRMS calcd for $C_{16}H_{19}O_2ClNa$ $[M+Na]^+$: 301.0971, found 301.0961.

**(*E*)-7-Methyl-7-(3-(4-(trifluoromethyl)phenyl)allyl)oxepan-2-one (1c)**

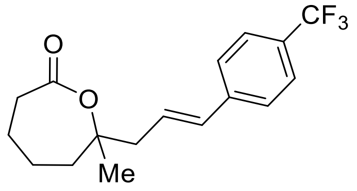


Prepared according to general procedure D using lactone **1i** (100 mg, 0.594 mmol), 4-(trifluoromethyl)styrene (263 μL, 1.78 mmol) and Hoveyda-Grubbs 2nd generation catalyst (4 mg, 5.94 μmol) in $CH_2Cl_2$ (1.5 mL). The crude mixture was purified by silica gel column chromatography (hexane/EtOAc, 100:0 to 80:20). The title product was obtained as a pale

yellow oil (71 mg, 0.227 mmol, 38%). $^1$H-NMR (400 MHz, $CDCl_3$) δ 1.50 (s, 3 H, $CH_3$), 1.60-1.74 (m, 1 H, $CH_2CH_2CH_aH_bCH_2C(O)O$), 1.76-1.95 (m, 5 H, $CH_2CH_2CH_aH_bCH_2C(O)O$ + $CH_2CH_2CH_2CH_2C(O)O$ + $CH_2CH_2CH_2CH_2C(O)O$), 2.56-2.84 (m, 4 H, $CH_2CH$=CHAr + $CH_2CH_2CH_2CH_2C(O)O$), 6.41 (dt, $J$ = 15.8, 7.0 Hz, 1 H, $CH_2CH$=CHAr), 6.50 (d, $J$ = 15.8 Hz, 1 H, $CH_2CH$=CHAr), 7.47 (d, $J$ = 8.2 Hz, 2 H, Ar*H*), 7.57 (d, $J$ = 8.2 Hz, 2 H, Ar*H*) ppm; $^{13}$C-NMR (100 MHz, $CDCl_3$) δ 23.4 ($CH_2CH_2CH_2CH_2C(O)O$), 24.0 ($CH_2CH_2CH_2CH_2C(O)O$), 24.6 ($CH_3$), 37.4 ($CH_2CH_2CH_2CH_2C(O)O$), 38.8 ($CH_2CH_2CH_2CH_2C(O)O$), 46.7 ($CH_2CH$=CHAr), 82.9 ($CH_3CO$), 125.5 (q, $J$ = 3.9 Hz, Ar*C*H), 126.4 (Ar*C*H), 127.4 ($CH_2CH$=CHAr), 129.2 (q, $J$ = 32.1 Hz, Ar*C*$CF_3$), 132.7 ($CH_2CH$=*C*HAr), 140.5 (Ar*C*), 174.7 (*C*(O)O) ppm, Ar$CF_3$ not observed; $^{19}$F-NMR (376 MHz, $CDCl_3$) δ –62.5 (s, $CF_3$) ppm; IR $\nu_{max}$ (thin film/cm$^{-1}$): 2943, 1711 (C=O), 1323, 1160, 1124, 1068; HRMS calcd for $C_{17}H_{19}O_2F_3Na$ $[M+Na]^+$: 335.1235, found 335.1237.

**(*E*)-7-(3-(4-Bromophenyl)allyl)-7-methyloxepan-2-one (1d)**

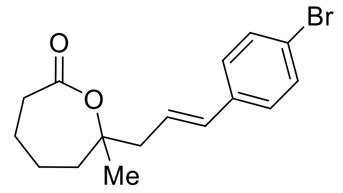


Prepared according to general procedure D using lactone **1i** (100 mg, 0.594 mmol), 4-bromostyrene (233 μL, 1.78 mmol) and Hoveyda-Grubbs 2$^{nd}$ generation catalyst (4 mg, 5.94 μmol) in $CH_2Cl_2$ (1.5 mL). The crude mixture was purified by silica gel column chromatography (hexane/EtOAc, 100:0 to 85:15). The title product was obtained as a palw yellow oil (55 mg, 0.170 mmol, 29%). $^1$H-NMR (400 MHz, $CDCl_3$) δ 1.49 (s, 3 H, $CH_3$), 1.55-1.73 (m, 1 H, $CH_2CH_2CH_aH_bCH_2C(O)O$), 1.74-1.93 (m, 5 H, $CH_2CH_2CH_aH_bCH_2C(O)O$ + $CH_2CH_2CH_2CH_2C(O)O$ + $CH_2CH_2CH_2CH_2C(O)O$), 2.52-2.83 (m, 4 H, $CH_2CH$=CHAr + $CH_2CH_2CH_2CH_2C(O)O$), 6.28 (dt, $J$ = 16.0, 7.2 Hz, 1 H, $CH_2CH$=CHAr), 6.40 (d, $J$ = 16.0

Hz, 1 H, $CH_2CH=CHAr$), 7.24 (d, $J$ = 8.6 Hz, 2 H, Ar$H$), 7.43 (d, $J$ = 8.6 Hz, 2 H, Ar$H$) ppm; $^{13}$C-NMR (100 MHz, $CDCl_3$) δ 23.3 ($CH_2CH_2\mathit{C}H_2CH_2C(O)O$), 23.9 ($CH_2\mathit{C}H_2CH_2CH_2C(O)O$), 24.6 ($\mathit{C}H_3$), 37.4 ($CH_2CH_2CH_2\mathit{C}H_2C(O)O$), 38.6 ($\mathit{C}H_2CH_2CH_2CH_2C(O)O$), 46.5 ($\mathit{C}H_2CH=CHAr$), 83.0 ($CH_3\mathit{C}O$), 121.1 (Ar$C$Br), 125.3 ($CH_2\mathit{C}H=CHAr$), 127.7 (Ar$C$H), 131.6 (Ar$C$H), 132.7 ($CH_2CH=\mathit{C}HAr$), 136.0 (Ar$C$), 174.7 ($\mathit{C}(O)O$) ppm; IR $\nu_{max}$ (thin film/$cm^{-1}$): 2933, 1711 (C=O), 1486, 1289, 1178; HRMS calcd for $C_{16}H_{19}O_2BrNa$ $[M+Na]^+$: 345.0466, found 345.0477.

**(*E*)-7-(3-(2-Fluorophenyl)allyl)-7-methyloxepan-2-one (1e)**

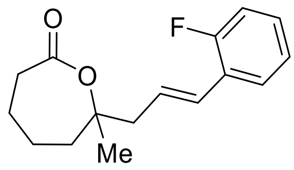


Prepared according to general procedure D using lactone **1i** (300 mg, 1.78 mmol), 4-fluorostyrene (640 μL, 5.35 mmol) and Hoveyda-Grubbs 2nd generation catalyst (11 mg, 178 μmol) in $CH_2Cl_2$ (4.5 mL). The crude miture was purified by silica gel column chromatography (hexane/EtOAc, 100:0 to 92:8). The title product was obtained as a light orange oil (274 mg, 1.05 mmol, 59%). $^1$H-NMR (400 MHz, $CDCl_3$) δ 1.50 (s, 3 H, $C\mathit{H}_3$), 1.64–1.73 (m, 1 H, $CH_2CH_2C\mathit{H}_aH_bCH_2C(O)O$), 1.75–1.94 (m, 5 H, $CH_2CH_2CH_a\mathit{H}_bCH_2C(O)O$ + $CH_2C\mathit{H}_2CH_2CH_2C(O)O$ + $C\mathit{H}_2CH_2CH_2CH_2C(O)O$), 2.57–2.84 (m, 4 H, $C\mathit{H}_2CH=CHAr$ + $CH_2CH_2CH_2C\mathit{H}_2C(O)O$), 6.35 (dt, $J$ = 16.0, 7.4 Hz, 1 H, $C\mathit{H}=CHAr$), 6.63 (d, $J$ = 16.0 Hz, 1 H, $CH=C\mathit{H}Ar$), 7.03 (ddd, $J$ = 10.8, 8.0, 1.2 Hz, 1 H, Ar$H$), 7.09 (td, $J$ = 7.6, 1.2 Hz, 1 H, Ar$H$), 7.16–7.24 (m, 1 H, Ar$H$), 7.48 (td, $J$ = 7.6, 1.6 Hz, 1 H, Ar$H$) ppm; $^{13}$C-NMR (100 MHz, $CDCl_3$) δ 23.3 ($CH_2CH_2\mathit{C}H_2CH_2C(O)O$), 23.9 ($CH_2\mathit{C}H_2CH_2CH_2C(O)O$), 24.6 ($\mathit{C}H_3$), 37.4 ($CH_2CH_2CH_2\mathit{C}H_2C(O)O$), 38.6 ($\mathit{C}H_2CH_2CH_2CH_2C(O)O$), 46.8 ($\mathit{C}H_2CH=CHAr$), 83.1 ($CH_3\mathit{C}O$), 115.7 (d, $J$ = 22.4 Hz,

Ar*C*H), 124.0 (d, *J* = 3.9 Hz, Ar*C*H), 124.8 (d, *J* = 12.6 Hz, Ar*C*), 126.1 (d, *J* = 3.9 Hz, $CH_2$CH=*C*HAr), 127.1 (d, *J* = 4.9 Hz, $CH_2$*C*H=CHAr), 127.1 (d, *J* = 3.9 Hz, Ar*C*H), 128.7 (d, *J* = 7.8 Hz, Ar*C*H), 159.9 (d, *J* = 246.9 Hz, Ar*C*F), 174.8 ($CH_2CH_2CH_2CH_2$*C*(O)O) ppm; $^{19}$F-NMR (376 MHz, $CDCl_3$) δ –118.8 (m, Ar*F*) ppm; IR $\nu_{max}$ (thin film/cm$^{-1}$): 2935, 2864, 1713, 1486, 1456, 1287, 1228, 1179, 1106, 1018; HRMS calcd for $C_{16}H_{19}O_2FNa$ [M+Na]$^+$: 285.1261, found 285.1263.

**(*E*)-7-Methyl-7-(3-(*p*-tolyl)allyl)oxepan-2-one (1f)**

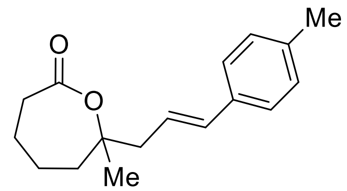


Prepared according to general procedure D using lactone **1i** (100 mg, 0.594 mmol), 4-methylstyrene (235 μL, 1.78 mmol) and Hoveyda-Grubbs 2$^{nd}$ generation catalyst (4 mg, 5.94 μmol) in $CH_2Cl_2$ (1.5 mL). The crude mixture was purified by silica gel column chromatography (hexane/EtOAc, 100:0 to 80:20). The title product was obtained as a colourless oil (50 mg, 0.194 mmol, 33%). $^1$H-NMR (400 MHz, $CDCl_3$) δ 1.49 (s, 3 H, C*H*$_3$CO), 1.62–1.74 (m, 1 H, $CH_2CH_2C$*H*$_a$H$_b$$CH_2$C(O)O), 1.75–1.94 (m, 5 H, $CH_2CH_2CH_a$*H*$_b$$CH_2$C(O)O + $CH_2$C*H*$_2$$CH_2CH_2$C(O)O + C*H*$_2$$CH_2CH_2CH_2$C(O)O), 2.34 (s, 3 H, ArC*H*$_3$), 2.55–2.83 (m, 4 H, C*H*$_2$CH=CHAr + $CH_2CH_2CH_2$C*H*$_2$C(O)O), 6.21 (dt, *J* = 15.8, 7.6 Hz, 1 H, $CH_2$C*H*=CHAr), 6.43 (d, *J* = 15.8 Hz, 1 H, $CH_2$CH=C*H*Ar), 7.12 (d, *J* = 7.8 Hz, 2 H, Ar*H*), 7.28 (d, *J* = 7.8 Hz, 2 H, Ar*H*) ppm; $^{13}$C-NMR (100 MHz, $CDCl_3$) δ 21.2 (Ar*C*H$_3$), 23.4 ($CH_2CH_2$*C*H$_2$$CH_2$C(O)O), 24.0 ($CH_2$*C*H$_2$$CH_2CH_2$C(O)O), 24.8 (*C*H$_3$CO), 37.4 ($CH_2CH_2CH_2$*C*H$_2$C(O)O), 38.5 (*C*H$_2$$CH_2CH_2CH_2$C(O)O), 46.4 (*C*H$_2$CH=CHAr), 83.3 ($CH_3$*C*O), 123.3 ($CH_2$*C*H=CHAr), 126.1 (Ar*C*H), 129.3 (Ar*C*H), 133.8 ($CH_2$CH=*C*HAr), 134.3 (Ar*C*CH$_3$), 137.2 (Ar*C*), 174.9 (*C*(O)O) ppm; IR $\nu_{max}$ (thin film/cm$^{-1}$): 2932, 1712

(C=O), 1512, 1446, 1287, 1176, 1101, 1017, 970; HRMS calcd for $C_{17}H_{22}O_2Na$ $[M+Na]^+$: 281.1517, found 281.1526.

**(*E*)-7-Methyl-7-(3-(*m*-tolyl)allyl)oxepan-2-one (1g)**

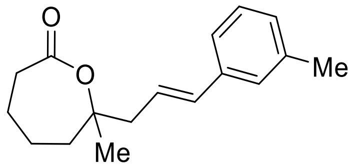


Prepared according to general procedure D using lactone **1i** (100 mg, 0.594 mmol), 3-methylstyrene (237 μL, 1.78 mmol) and Hoveyda-Grubbs 2nd generation catalyst (4 mg, 5.94 μmol) in $CH_2Cl_2$ (1.5 mL). The crude mixture was purified by silica gel column chromatography (hexane/EtOAc, 100:0 to 80:20). The title product was obtained as a colourless oil (71 mg, 0.275 mmol, 46%). $^1$H-NMR (400 MHz, $CDCl_3$) δ 1.49 (s, 3 H, C$H_3$CO), 1.63–1.73 (m, 1 H, $CH_2CH_2C$$H_a$$H_b$$CH_2C$(O)O), 1.75–1.95 (m, 5 H, $CH_2CH_2CH_a$$H_b$$CH_2C$(O)O + $CH_2C$$H_2$$CH_2CH_2C$(O)O + C$H_2$$CH_2CH_2CH_2C$(O)O), 2.35 (s, 3 H, ArC$H_3$), 2.55–2.83 (m, 4 H, C$H_2$CH=CHAr + $CH_2CH_2CH_2C$$H_2$C(O)O), 6.26 (dt, *J* = 15.6, 7.2 Hz, 1 H, $CH_2C$*H*=CHAr), 6.43 (d, *J* = 15.6 Hz, 1 H, $CH_2$CH=C*H*Ar), 7.05 (d, *J* = 6.8 Hz, 1 H, Ar*H*), 7.14–7.24 (m, 3 H, Ar*H*) ppm; $^{13}$C-NMR (100 MHz, $CDCl_3$) δ 21.4 (Ar*C*$H_3$), 23.4 ($CH_2CH_2$*C*$H_2CH_2C$(O)O), 24.0 ($CH_2$*C*$H_2CH_2CH_2C$(O)O), 24.7 (*C*$H_3$CO), 37.4 ($CH_2CH_2CH_2$*C*$H_2$C(O)O), 38.5 (*C*$H_2CH_2CH_2CH_2C$(O)O), 46.4 (*C*$H_2$CH=CHAr), 83.2 ($CH_3$*C*O), 123.4 (Ar*C*H), 124.1 ($CH_2$*C*H=CHAr), 126.8 (Ar*C*H), 128.2 (Ar*C*H), 128.4 (Ar*C*H), 134.0 ($CH_2$CH=*C*HAr), 137.0 (Ar*C*), 138.1 (Ar*C*$CH_3$), 174.8 (*C*(O)O) ppm; IR $\nu_{max}$ (thin film/$cm^{-1}$): 2933, 1711 (C=O), 1446, 1287, 1178, 1103, 1017, 968; HRMS calcd for $C_{17}H_{22}O_2Na$ $[M+Na]^+$: 281.1517, found 281.1530.

**(*E*)-7-Methyl-7-(3-(naphthalen-2-yl)allyl)oxepan-2-one (1h)**

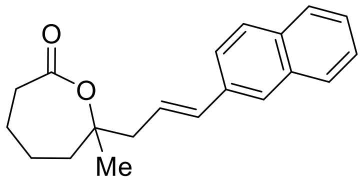


Prepared according to general procedure D using lactone **1i** (100 mg, 0.594 mmol), 3-methylstyrene (275 μL, 1.78 mmol) and Hoveyda-Grubbs 2nd generation catalyst (4 mg, 5.94 μmol) in $CH_2Cl_2$ (1.5 mL). The crude mixture was purified by silica gel column chromatography (hexane/EtOAc, 100:0 to 85:15). The title product was obtained as a colourless oil (61 mg, 0.207 mmol, 35%). $^1$H-NMR (400 MHz, $CDCl_3$) δ 1.53 (s, 3 H, C$H_3$), 1.64–1.75 (m, 1 H, $CH_2CH_2C$$H_a$$H_b$$CH_2C(O)O$), 1.61–1.97 (m, 5 H, $CH_2CH_2CH_a$$H_b$$CH_2C(O)O$ + $CH_2C$$H_2$$CH_2CH_2C(O)O$ + C$H_2$$CH_2CH_2CH_2C(O)O$), 2.60–2.85 (m, 4 H, C$H_2$CH=CHAr + $CH_2CH_2CH_2C$$H_2$C(O)O), 6.41 (dt, *J* = 16.0, 7.2 Hz, 1 H, $CH_2C$*H*=CHAr), 6.63 (d, *J* = 16.0 Hz, 1 H, $CH_2$CH=C*H*Ar), 7.41–7.50 (m, 2 H, Ar*H*), 7.61 (dd, *J* = 8.8, 1.6 Hz, 1 H, Ar*H*), 7.72 (bs, 1 H, Ar*H*), 7.76–7.84 (m, 3 H, Ar*H*) ppm; $^{13}$C-NMR (100 MHz, $CDCl_3$) δ 23.4 ($CH_2CH_2$*C*$H_2CH_2C(O)O$), 24.0 ($CH_2$*C*$H_2CH_2CH_2C(O)O$), 24.7 (*C*$H_3$), 37.4 ($CH_2CH_2CH_2$*C*$H_2C(O)O$), 38.7 (*C*$H_2CH_2CH_2CH_2C(O)O$), 46.6 (*C*$H_2$CH=CHAr), 83.3 (*C*$H_3$*C*O), 123.6 (Ar*C*H), 124.9 ($CH_2$*C*H=CHAr), 125.8 (Ar*C*H), 125.9 (Ar*C*H), 126.3 (Ar*C*H), 127.7 (Ar*C*H), 127.9 (Ar*C*H), 128.2 (Ar*C*H), 132.9 (Ar*C*), 133.6 (Ar*C*), 134.1 ($CH_2$CH=*C*HAr), 134.6 (Ar*C*), 174.8 (*C*(O)O) ppm; IR $\nu_{max}$ (thin film/cm$^{-1}$): 2933, 1709 (C=O), 1445, 1288, 1178, 1102, 1017, 968; HRMS calcd for $C_{20}H_{22}O_2Na$ $[M+Na]^+$: 317.1517, found 317.1509.

**Ethyl 6-oxo-heptanoate (S3)**[3]

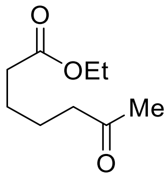


To a flask charged with $FeCl_3$ (1.00 g, 6.20 mmol) were added dry EtOH (36.0 mL, 620 mmol) and 2-acetylcyclopentanone (15.0 mL, 124 mmol). The flask was sealed under $N_2$ and the mixture was stirred vigorously at 80 ºC. After 16 h the reaction was allowed to reach room temperature and the mixture was poured onto EtOAc (1.0 L) and washed with $H_2O$ (2 × 200 mL) and brine (200 mL). The organic layer was dried ($MgSO_4$) and concentrated under vacuum. The resulting crude mixture was purified by silica gel column chromatography (hexane/EtOAc, 100:0 to 80:20). The title product was obtained as a yellow oil (20.6 g, 120 mmol, 96%). $^1$H-NMR (400 MHz, $CDCl_3$) δ 1.26 (t, $J$ = 7.0 Hz, 3 H, $OCH_2CH_3$), 1.57–1.69 (m, 4 H, $CH_2CH_2CH_2CH_2C(O)O$ + $CH_2CH_2CH_2CH_2C(O)O$), 2.14 (s, 3 H, $CH_3C(O)$), 2.28–2.34 (m, 2 H, $CH_2CH_2CH_2CH_2C(O)O$), 2.42–2.50 (m, 2 H, $CH_2CH_2CH_2CH_2C(O)O$), 4.12 (q, $J$ = 7.0 Hz, 2 H, $OCH_2CH_3$) ppm; $^{13}$C-NMR (100 MHz, $CDCl_3$) δ 14.2 ($OCH_2CH_3$), 23.1 ($CH_2$), 24.4 ($CH_2$), 29.9 ($CH_3C(O)$), 34.0 ($CH_2CH_2CH_2CH_2C(O)O$), 43.3 ($CH_2CH_2CH_2CH_2C(O)O$), 60.3 ($OCH_2CH_3$), 173.4 ($C(O)O$), 208.7 ($CH_3C(O)$) ppm; IR $\nu_{max}$ (thin film/cm$^{-1}$): 2940, 1730 (C=O), 1714 (C=O), 1370, 1177, 1031; HRMS calcd for $C_9H_{17}O_3$ $[M+H]^+$: 173.1172, found 173.1173.

**Ethyl 5-(2-methyl-1,3-dioxolan-2-yl)pentanoate (S4)**

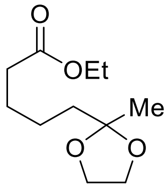


[3] Prepared using an adapted procedure from: Biswas, S.; Maiti, S.; Jana, U. *Eur. J. Org. Chem.* **2010**, 2861.

A solution of ketoester **S3** (5.00 g, 29.0 mmol), ethylene glycol (2.40 mL, 43.5 mmol) and *p*-toluenesulfonic acid monohydrate (110 mg, 0.58 mmol) in toluene was refluxed in a flask fitted with a Dean-Stark. After 23 h, the reaction mixture was allowed to cool down before it was poured onto a $Na_2CO_3$ solution in icy water and then extracted with $Et_2O$ (100 mL). The organic layer was washed with $H_2O$ (2 × 20 mL) and brine (20 mL), dried ($MgSO_4$) and concentrated under vacuum. The crude product (4.76 g, 22.0 mmol, 76%) was was used without further purification. $^1$H-NMR (400 MHz, $CDCl_3$) δ 1.26 (t, *J* = 7.1 Hz, 3 H, $OCH_2CH_3$), 1.31 (s, 3 H, $CH_3C$), 1.37–1.48 (m, 2 H, $CH_2$), 1.57–1.73 (m, 4 H, $CH_2$ + $CH_2C(OCH_2)_2$), 2.31 (t, *J* = 7.6 Hz, 2 H, $CH_2C(O)O$), 3.89–3.98 (m, 4 H, $C(OCH_2)_2$), 4.13 (q, *J* = 7.1 Hz, 2 H, $OCH_2CH_3$) ppm; $^{13}$C-NMR (100 MHz, $CDCl_3$) δ 14.2 ($OCH_2CH_3$), 23.6 ($CH_2$), 23.7 ($CH_3C$), 25.1 ($CH_2$), 34.3 ($CH_2C(O)O$), 38.8 ($CH_2C(OCH_2)_2$), 60.2 ($OCH_2CH_3$), 64.6 ($C(OCH_2)_2$), 109.9 ($C(OCH_2)_2$), 173.7 ($C(O)O$) ppm; IR $\nu_{max}$ (thin film/cm$^{-1}$): 2981, 2946, 2875, 1732 (C=O), 1375, 1244, 1177, 1064, 1038; HRMS calcd for $C_{11}H_{21}O_4$: 217.1434, found 217.1436.

**General procedure E: ester alkylation**

**Ethyl 2-methyl-5-(2-methyl-1,3-dioxolan-2-yl)pentanoate (S5)**

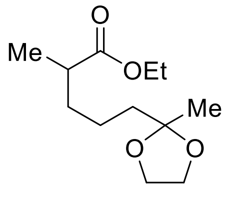


LDA was prepared by dropwise addition of 1.6 M *n*-BuLi in hexanes (3.5 mL, 5.55 mmol) to a stirred solution of diisopropylamine (780 μL, 5.55 mmol) in THF (7.7 mL) at −78 ºC. After 30 min, the solution was stirred at 0 ºC for 10 min and then cooled down back to −78 ºC. A solution of ester **S4** (1.00 g, 4.62 mmol) in THF (2.7 mL) was added dropwise over the course of 1 h. After the addition, the mixture was stirred for 1 h at −78 ºC, then at 0 ºC for 10

min and finally back at −78 °C before adding dropwise a solution of methyl iodide (350 μL, 5.55 mmol) in HMPA (970 μL, 5.55 mmol) over the course of 15 min. The resulting mixture was allowed to slowly warm up overnight. The reaction was quenched with saturated aqueous $NH_4Cl$ (10 mL), extracted with $Et_2O$ (3 × 20 mL), dried ($MgSO_4$) and concentrated *in vacuo*. The crude product was purified by silica gel column chromatography (hexane/EtOAc, 95:5) to afford the title product as a colourless oil (873 mg, 3.79 mmol, 82%). $^1$H-NMR (400 MHz, $CDCl_3$) δ 1.15 (d, *J* = 6.8 Hz, 3 H, $C\mathit{H}_3CH$), 1.26 (t, *J* = 7.2 Hz, 3 H, $OCH_2C\mathit{H}_3$), 1.31 (s, 3 H, $C\mathit{H}_3C(OCH_2)_2$), 1.35–1.47 (m, 3 H, $CH_2C\mathit{H}_2CH_2CHC(O)O$ + $CH_2CH_2C\mathit{H}_aH_bCHC(O)O$), 1.58–1.73 (m, 3 H, $CH_2CH_2CH_a\mathit{H}_bCHC(O)O$ + $C\mathit{H}_2CH_2CH_2CHC(O)O$, 2.37–2.48 (m, 1 H, $CH_2CH_2CH_2\mathit{C}HC(O)O$), 3.89–3.98 (m, 4 H, $CH_3C(OC\mathit{H}_2)_2$), 4.13 (q, *J* = 7.2 Hz, 2 H, $OC\mathit{H}_2CH_3$) ppm; $^{13}$C-NMR (100 MHz, $CDCl_3$) δ 14.2 ($OCH_2\mathit{C}H_3$), 17.0 ($\mathit{C}H_3CH$), 21.8 ($CH_2\mathit{C}H_2CH_2CHC(O)O$), 23.7 ($\mathit{C}H_3C(OCH_2)_2$), 33.9 ($CH_2CH_2\mathit{C}H_2CHC(O)O$), 39.0 ($\mathit{C}H_2CH_2CH_2CHC(O)O$)), 39.5 ($CH_2CH_2CH_2\mathit{C}HC(O)O$), 60.1 ($O\mathit{C}H_2CH_3$), 64.6 ($CH_3C(O\mathit{C}H_2)_2$), 109.9 ($CH_3\mathit{C}(OCH_2)_2$), 176.8 ($\mathit{C}(O)O$) ppm; IR $\nu_{max}$ (thin film/cm$^{-1}$): 2979, 2948, 2877, 1730 (C=O), 1464, 1376, 1178, 1150, 1041; HRMS calcd for $C_{12}H_{22}O_4Na$ $[M+Na]^+$: 253.1410, found 253.1404.

**Ethyl 2,2-dimethyl-5-(2-methyl-1,3-dioxolan-2-yl)pentanoate (S6)**

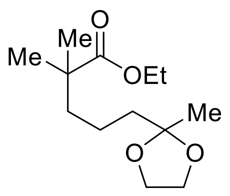


Prepared according to general procedure E using a solution of diisopropylamine (635 μL, 4.51 mmol) in THF (6.3 mL), 1.6 M *n*-BuLi in hexanes (2.8 mL, 4.51 mmol), a solution of ester **S5** (865 mg, 3.76 mmol) in THF (2.2 mL), methyl iodide (280 μL, 4.51 mmol) and HMPA (790 μL, 4.51 mmol). The crude product was purified by silica gel column

chromatography (hexane/EtOAc, 95:5) to afford the title product as a colourless oil (836 mg, 3.42 mmol, 91%). $^1$H-NMR (400 MHz, $CDCl_3$) δ 1.17 (s, 6 H, $(CH_3)_2C$), 1.25 (t, $J$ = 7.2 Hz, 3 H, $OCH_2CH_3$), 1.29–1.38 (m, 5 H, $CH_2CH_2CH_2CC(O)O$ + $CH_3C(OCH_2)_2$), 1.49–1.56 (m, 2 H, $CH_2CH_2CH_2CC(O)O$), 1.57–1.65 (m, 2 H, $CH_2CH_2CH_2CC(O)O$), 3.88–3.98 (m, 4 H, $CH_3C(OCH_2)_2$), 4.12 (q, $J$ = 7.2 Hz, 2 H, $OCH_2CH_3$) ppm; $^{13}$C-NMR (100 MHz, $CDCl_3$) δ 14.2 ($OCH_2CH_3$), 19.5 ($CH_2CH_2CH_2CC(O)O$), 23.7 ($CH_3C(OCH_2)_2$), 25.1 ($(CH_3)_2C$), 39.5 ($CH_2CH_2CH_2CC(O)O$), 40.7 ($CH_2CH_2CH_2CC(O)O$), 42.1 ($(CH_3)_2C$), 60.2 ($OCH_2CH_3$), 64.6 ($CH_3C(OCH_2)_2$), 109.9 ($CH_3C(OCH_2)_2$), 178.0 ($C(O)O$) ppm; IR $\nu_{max}$ (thin film/cm$^{-1}$): 2979, 2876, 1725 (C=O), 1474, 1376, 1148, 1060; HRMS calcd for $C_{13}H_{25}O_4$ $[M+H]^+$: 245.1747, found 245.1749.

**Ethyl 2,2-dimethyl-6-oxoheptanoate (S7)**

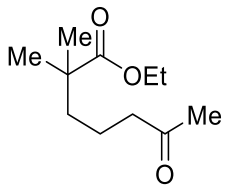


To a solution of acetal **S6** (826 mg, 3.38 mmol) in acetone (48 mL) was added *p*-toluenesulfonic acid monohydrate (1.30 g, 6.76 mmol). The resulting solution was stirred at room temperature for 3 h before addition of $NaHCO_3$ (50 mL). The mixture was extracted with $CH_2Cl_2$ (3 × 50 mL), dried ($MgSO_4$) and concentrated *in vacuo*. The crude product was purified by filtration through a silica plug with hexane/EtOAc (9:1) as the eluent to afford the title product as a pale yellow oil (651 mg, 3.25 mmol, 96%). $^1$H-NMR (400 MHz, $CDCl_3$) δ 1.17 (s, 6 H, $(CH_3)_2C$), 1.25 (t, $J$ = 7.3 Hz, 3 H, $OCH_2CH_3$), 1.47–1.57 (m, 4 H, $CH_2CH_2CH_2CC(O)O$ + $CH_2CH_2CH_2CC(O)O$), 2.13 (s, 3 H, $CH_3C(O)$), 2.41 (t, $J$ = 7.0 Hz, 2 H, $CH_2CH_2CH_2CC(O)O$), 4.12 (q, $J$ = 7.3 Hz, 2 H, $OCH_2CH_3$) ppm; $^{13}$C-NMR (100 MHz, $CDCl_3$) δ 14.2 ($OCH_2CH_3$), 19.3 ($CH_2CH_2CH_2CC(O)O$), 25.0 ($(CH_3)_2C$), 29.8 ($CH_3C(O)$)

39.9 ($CH_2CH_2\mathit{C}H_2CC(O)O$), 42.1 ($(CH_3)_2\mathit{C}$), 44.0 ($\mathit{C}H_2CH_2CH_2CC(O)O$), 60.3 ($O\mathit{C}H_2CH_3$), 177.7 ($\mathit{C}(O)O$), 208.6 ($CH_3\mathit{C}(O)$) ppm; IR $\nu_{max}$ (thin film/$cm^{-1}$): 2977, 1719 (C=O), 1363, 1174, 1133; HRMS calcd for $C_{11}H_{20}O_3Na$ $[M+Na]^+$: 223.1305, found 223.1308.

**Ethyl (*E*)-6-hydroxy-2,2,6-trimethyl-9-phenylnon-8-enoate (S8)**

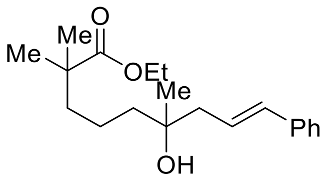


To a flask charged with $NiI_2$ (60 mg, 0.192 mmol) was added a 0.1 M $SmI_2$ solution in THF (96 mL, 9.60 mmol). The mixture was cooled to 0 ºC and then a solution of ketoester **S7** (641 mg, 3.20 mmol) in THF (1.5 mL) was added, followed by addition of a solution of cinnamyl bromide (694 mg, 3.52 mmol) in THF (1.0 mL). The resulting solution was stirred at 0 ºC for 1.5 h before opening the flask and allowing the mixture to decolourise. A saturated solution of Rochelle's salt (50 mL) was added, layers were separated and the aqueous layer was extracted with $Et_2O$ (3 × 75 mL). The combined organic layers were washed with brine, dried ($MgSO_4$) and concentrated *in vacuo*. The crude product was purified by silica gel column chromatography (hexanes/EtOAc, 95:5 to 90:10) to afford the title product as a pale yellow oil (478 mg, 1.50 mmol, 47%). $^1$H-NMR (400 MHz, $CDCl_3$) δ 1.19 (s, 6 H, $(C\mathit{H}_3)_2C$), 1.22 (s, 3 H, $C\mathit{H}_3COH$), 1.25 (t, *J* = 7.2 Hz, 3 H, $OCH_2C\mathit{H}_3$), 1.29–1.38 (m, 2 H, $CH_2C\mathit{H}_2CH_2CC(O)O$), 1.44 (s, 1 H, O*H*), 1.45–1.59 (m, 2 H, $C\mathit{H}_2CH_2CH_2CC(O)O$), 1.51–1.56 (m, 2 H, $CH_2CH_2C\mathit{H}_2CC(O)O$), 2.32–2.42 (m, 2 H, $C\mathit{H}_2CH{=}CHPh$), 4.13 (q, *J* = 7.2 Hz, 2 H, $OC\mathit{H}_2CH_3$), 6.22–6.31 (m, 1 H, $CH_2C\mathit{H}{=}CHPh$), 6.46 (d, *J* = 16.0 Hz, 1 H, $CH_2CH{=}C\mathit{H}Ph$), 7.23 (t, *J* = 7.5 Hz, 1 H, Ar*H*), 7.32 (t, *J* = 7.5 Hz, 2 H, Ar*H*), 7.38 (d, *J* = 7.5 Hz, 2 H, Ar*H*) ppm; $^{13}$C-NMR (100 MHz, $CDCl_3$) δ 14.3 ($OCH_2\mathit{C}H_3$), 19.3 ($CH_2\mathit{C}H_2CH_2CC(O)O$), 25.1 ($(\mathit{C}H_3)_a(CH_3)_bC$), 25.2 ($(CH_3)_a(\mathit{C}H_3)_bC$), 26.9 ($\mathit{C}H_3COH$), 41.1

($CH_2CH_2\mathit{C}H_2CC(O)O$), 42.2 ($CH_2CH_2CH_2\mathit{C}C(O)O$), 42.3 ($\mathit{C}H_2CH_2CH_2CC(O)O$), 45.6 ($\mathit{C}H_2CH{=}CHPh$), 60.2 ($O\mathit{C}H_2CH_3$), 72.7 ($CH_3\mathit{C}OH$), 125.5 ($CH_2\mathit{C}H{=}CHPh$), 126.1 (Ar*C*H), 127.3 (Ar*C*H), 128.5 (Ar*C*H), 133.7 ($CH_2CH{=}\mathit{C}HPh$), 137.3 (Ar*C*), 178.0 (*C*(O)O) ppm; IR $\nu_{max}$ (thin film/$cm^{-1}$): 3499 (O-H), 3026, 2972, 1725 (C=O), 1473, 1449, 1386, 1366, 1274, 1148; HRMS calcd for $C_{20}H_{30}O_3Na$ $[M+Na]^+$: 341.2987, found 341.2087.

**7-Cinnamyl-3,3,7-trimethyloxepan-2-one (1j)**

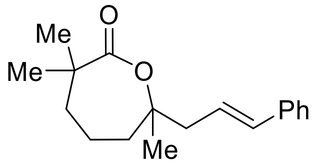


To a solution of hydroxyester **S8** (472 mg, 1.48 mmol) in MeOH (16.6 mL) was added a solution of KOH (416 mg, 7.41 mmol) in $H_2O$ (3.3 mL). The resulting mixture was stirred at 50 ºC for 40 h. The mixture was allowed to cool down to room temperature and was then quenched with a mixture of saturated aqueous $NaHCO_3/H_2O$ (1:2, 15 mL) and washed with $CH_2Cl_2$ (30 mL). The aqueous layer was acidified with aqueous 1 N HCl to pH = 1-2 and was then extracted with $CH_2Cl_2$ (3 × 30 mL). The organic layers were dried ($MgSO_4$) and concentrated *in vacuo*. The resulting crude mixture was dissolved in dry $CH_2Cl_2$ (50 mL) and was added dropwise over the course of 18 h to a solution of Shiina's reagent (651 mg, 1.89 mmol), DMAP (18 mg, 0.15 mmol) and triethylamine (1.2 mL, 8.7 mmol) in dry $CH_2Cl_2$ (430 mL) at 40 ºC. The mixture was then stirred at 40 ºC for 3 h. The reaction mixture was then cooled down to 0 ºC and quenched with $NaHCO_3$ (75 mL). Layers were separated and the aqueous fraction was extracted with $CH_2Cl_2$ (2 × 75 mL). The combined organic fractions were washed with saturated aqueous $NH_4Cl$ (2 × 100 mL) and brine (100 mL), dried ($MgSO_4$) and concentrated *in vacuo*. The crude product was purified by silica gel column chromatography (hexanes/EtOAc, 95:5) to afford the title product as a yellow oil (194 mg,

0.712 mmol, 49%). $^1$H-NMR (400 MHz, $CDCl_3$) δ 1.24 (s, 6 H, $(C\mathit{H}_3)_2C$), 1.49 (s, 3 H, $C\mathit{H}_3CO$), 1.47–1.63 (m, 2 H, $CH_2CH_2C\mathit{H}_2CC(O)O$), 1.65–1.81 (m, 4 H, $C\mathit{H}_2CH_2CH_2CC(O)O$ + $CH_2C\mathit{H}_2CH_2CC(O)O$), 2.50 (dd, *J* = 14.0, 8.0 Hz, 1 H, $C\mathit{H}_aH_bCH{=}CHPh$), 2.76 (ddd, *J* = 14.0, 6.5, 1.2 Hz, 1 H, $CH_a\mathit{H}_bCH{=}CHPh$), 6.31 (ddd, *J* = 15.8, 8.0, 6.5 Hz, 1 H, $CH_2C\mathit{H}{=}CHPh$), 6.47 (d, *J* = 15.8 Hz, 1 H, $CH_2CH{=}C\mathit{H}Ph$), 7.22 (t, *J* = 7.2 Hz, 1 H, Ar*H*), 7.31 (t, *J* = 7.2 Hz, 2 H, Ar*H*), 7.39 (d, *J* = 7.2 Hz, 2 H, Ar*H*) ppm; $^{13}$C-NMR (100 MHz, $CDCl_3$) δ 19.5 ($CH_2\mathit{C}H_2CH_2CC(O)O$), 25.3 ($\mathit{C}H_3CO$), 25.4 ($(\mathit{C}H_3)_a(CH_3)_bC$), 25.7 ($(CH_3)_a(\mathit{C}H_3)_bC$), 37.9 ($\mathit{C}H_2CH_2CH_2CC(O)O$), 40.0 ($CH_2CH_2\mathit{C}H_2CC(O)O$), 44.1 ($CH_2CH_2CH_2\mathit{C}C(O)O$), 45.1 ($\mathit{C}H_2CH{=}CHPh$), 82.9 ($CH_3\mathit{C}O$), 125.4 ($CH_2\mathit{C}H{=}CHPh$), 126.1 (Ar*C*H), 127.2 (Ar*C*H), 128.5 (Ar*C*H), 133.3 ($CH_2CH{=}\mathit{C}HPh$), 137.4 (Ar*C*), 182.6 (*C*(O)O) ppm; IR $\nu_{max}$ (thin film/$cm^{-1}$): 2926, 2855, 1731 (C=O), 1450, 1087, 1063; HRMS calcd for $C_{18}H_{24}O_2Na$ $[M+Na]^+$: 295.1669, found 295.1665.

**Allyl 1-benzyl-2-oxocyclohexane-1-carboxylate (S9)[4]**

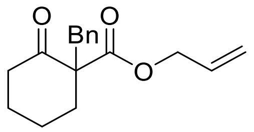


Prepared according to general procedure A using diallyl pimelate[1] (10.0 g, 41.6 mmol), 60% NaH in mineral oil (1.83 g, 45.8 mmol) and benzyl bromide (6.4 mL, 54.1 mmol) in THF (35 mL). The crude product was purified by silica gel column chromatography (hexane/$Et_2O$, 95:5) to afford the ketoester as a colourless oil (5.04 g, 32.9 mmol, 79%). $^1$H-NMR (400 MHz, $CDCl_3$) δ 1.42–1.53 (m, 1 H, $C\mathit{H}_aH_bCH_2CH_2CH_2C(O)$), 1.57–1.80 (m, 3 H, $CH_2C\mathit{H}_2CH_2CH_2C(O)$ + $CH_2CH_2C\mathit{H}_aH_bCH_2C(O)$), 1.96–2.08 (m, 1 H,

[4] Prepared according to the procedure reported in: Behenna, D. C.; Mohr, J. T.; Sherden, N. H.; Marinescu, S. C.; Harned, A. M.; Tani, K.; Seto, M.; Ma. S.; Novák, Z.; Krout, M. R.; McFadden, R. M.; Roizen, J. L.; Enquist, Jr., J. A.; White, D. E.; Levine, S. R.; Petrova, K. V.; Iwashita, A.; Virgil, S. C.; Stoltz, B. M. *Chem. Eur. J.* **2011**, *17*, 14199.

$CH_2CH_2CH_a\mathit{H}_bCH_2C(O)$), 2.36–2.55 (m, 3 H, $CH_a\mathit{H}_bCH_2CH_2CH_2C(O)$ + $CH_2CH_2CH_2C\mathit{H}_2C(O)$), 2.90 (d, $J$ = 13.8 Hz, 1 H, $CC\mathit{H}_aH_bAr$), 3.33 (d, $J$ = 13.8 Hz, 1 H, $CCH_a\mathit{H}_bAr$), 4.47−4.60 (m, 2 H, $OC\mathit{H}_2CH{=}CH_2$), 5.21−5.33 (m, 2 H, $OCH_2CH{=}C\mathit{H}_2$), 5.75−5.88 (m, 1 H, $OCH_2C\mathit{H}{=}CH_2$), 7.12 (d, $J$ = 6.6 Hz, 2 H, Ar$\mathit{H}$), 7.18−7.26 (m, 3 H, Ar$\mathit{H}$) ppm; $^{13}$C-NMR (100 MHz, $CDCl_3$) δ 22.4 ($CH_2\mathit{C}H_2CH_2CH_2C(O)$), 27.6 ($CH_2CH_2\mathit{C}H_2CH_2C(O)$), 35.9 ($\mathit{C}H_2CH_2CH_2CH_2C(O)$), 40.4 ($C\mathit{C}H_2Ar$), 41.3 ($CH_2CH_2CH_2\mathit{C}H_2C(O)$), 62.2 ($\mathit{C}CH_2Ar$), 65.8 ($O\mathit{C}H_2CH{=}CH_2$), 119.2 ($OCH_2CH{=}\mathit{C}H_2$), 126.7 (Ar$\mathit{C}$H), 128.0 (Ar$\mathit{C}$H), 130.3 (Ar$\mathit{C}$H), 131.3 ($OCH_2\mathit{C}H{=}CH_2$), 136.5 (Ar$\mathit{C}$), 170.6 ($\mathit{C}(O)O$), 207.1 ($\mathit{C}(O)$) ppm; IR $\nu_{max}$ (thin film/cm$^{-1}$): 2941, 1712 (C=O), 1495, 1452,1179; HRMS calcd for $C_{17}H_{20}O_3Na$ $[M+Na]^+$: 295.1319, found 295.1310.

**2-Allyl-2-benzylcyclohexan-1-one (S10)**[5]

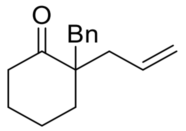


Prepared according to general procedure B using ketoester **S9** (2.60 g, 9.50 mmol), $Pd_2(dba)_3$ (0.46 g, 0.50 mmol) and $PPh_3$ (0.50 g, 1.90 mmol) in THF (70 mL). The crude product was purified by silica gel column chromatography (hexane/EtOAc, 97:3 to 90:10) to afford the title compound as a colourless oil (1.85 g, 8.08 mmol, 85%). $^1$H-NMR (400 MHz, $CDCl_3$) δ 1.65−1.92 (m, 6 H, $C\mathit{H}_2CH_2CH_2CH_2C(O)$ + $CH_2C\mathit{H}_2CH_2CH_2C(O)$ + $CH_2CH_2C\mathit{H}_2CH_2C(O)$), 2.29 (d, $J$ = 7.2 Hz, 2 H, $C\mathit{H}_2CH{=}CH_2$), 2.39−2.52 (m, 2 H, $CH_2CH_2CH_2C\mathit{H}_2C(O)$), 2.92 (s, 2 H, $C\mathit{H}_2Ar$) 5.03−5.14 (m, 2 H, $CH_2CH{=}C\mathit{H}_2$), 5.68–5.81 (m, 1 H, $CH_2C\mathit{H}{=}CH_2$), 7.12 (d, $J$ = 7.2 Hz, 2 H, Ar$\mathit{H}$), 7.18−7.30 (m, 3 H, Ar$\mathit{H}$) ppm; $^{13}$C-NMR (100 MHz, $CDCl_3$) δ 20.8 ($CH_2\mathit{C}H_2CH_2CH_2C(O)$), 26.8 ($CH_2CH_2\mathit{C}H_2CH_2C(O)$), 35.5 ($\mathit{C}H_2CH_2CH_2CH_2C(O)$), 39.2 ($CH_2CH_2CH_2\mathit{C}H_2C(O)$), 39.6 ($\mathit{C}H_2Ar$), 40.9 ($\mathit{C}H_2CH{=}CH_2$), 52.6 ($\mathit{C}CH_2CH{=}CH_2$), 118.2

[5] The product matched the compound reported in ref. 4.

($CH_2CH=CH_2$), 126.3 (Ar*C*H) 128.0 (Ar*C*H), 130.6 (Ar*C*H), 133.8 ($CH_2$*C*H=$CH_2$), 137.6 (Ar*C*), 214.1 (*C*(O)) ppm; IR $\nu_{max}$ (thin film/$cm^{-1}$): 2934, 1702 (C=O), 1495, 1452, 1124; HRMS calcd for $C_{16}H_{20}ONa$ $[M+Na]^+$: 251.1424, found 251.1412.

**7-Allyl-7-benzyloxepan-2-one (1k)**

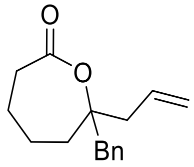

Prepared according to general procedure C using ketone **S10** (694 mg, 3.04 mmol), *m*CPBA (747 mg, 3.33 mmol) and $NaHCO_3$ (509 mg, 6.06 mmol) in $CH_2Cl_2$ (7 mL). The resulting crude mixture was purified by silica gel column chromatography (hexane/EtOAc, 95:5 to 80:20). The title product was obtained as a colourless oil (125 mg, 0.515 mmol, 17%). $^1$H-NMR (400 MHz, $CDCl_3$) δ 1.61−1.99 (m, 6 H, $CH_2CH_2CH_2CH_2C(O)O$ + $CH_2CH_2CH_2CH_2C(O)O$ + $CH_2CH_2CH_2CH_2C(O)O$), 2.45−2.61 (m, 2 H, $CCH_2CH=CH_2$), 2.61−2.78 (m, 2 H, $CH_2CH_2CH_2CH_2C(O)O$), 2.96−3.07 (m, 2 H, $CCH_2Ar$), 5.11−5.33 (m, 2 H, $CH_2CH=CH_2$), 5.84−5.98 (m, 1 H, $CH_2CH=CH_2$), 7.23−7.35 (m, 5 H, Ar*H*) ppm; $^{13}$C-NMR (100 MHz, $CDCl_3$) δ 23.0 ($CH_2CH_2CH_2CH_2C(O)O$), 23.1 ($CH_2CH_2CH_2CH_2C(O)O$), 36.0 ($CH_2CH_2CH_2CH_2C(O)O$), 37.3 ($CH_2CH_2CH_2CH_2C(O)O$), 41.8 ($CH_2CH=CH_2$), 45.3 ($CH_2Ar$), 84.5 ($(CH_2)_4CO$), 119.4 ($CH_2CH=CH_2$), 126.7 (Ar*C*H), 128.1 (Ar*C*H), 130.8 (Ar*C*H), 132.3 ($CH_2CH=CH_2$), 136.1 (Ar*C*), 174.3 (*C*(O)) ppm; IR $\nu_{max}$ (thin film/$cm^{-1}$): 2934, 1714 (C=O), 1452, 1284, 1169; HRMS cald for $C_{16}H_{20}O_2Na$ $[M+Na]^+$: 267.1354, found 267.1361.

**7-Benzyl-7-cinnamyloxepan-2-one (1l)**

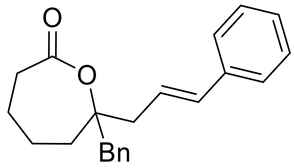


Prepared according to general procedure D using lactone **1k** (0.15 g, 0.60 mmol), styrene (210 μL, 1.80 mmol) and Hoveyda-Grubbs 2nd generation catalyst (4 mg, 5.94 μmol) in $CH_2Cl_2$ (1.5 mL). The crude mixture was purified by silica gel column chromatography (hexane/EtOAc, 100:0 to 80:20). The title product was obtained as a colourless oil (79 mg, 0.246 mmol, 41%). $^1$H-NMR (400 MHz, $CDCl_3$) δ 1.62−2.02 (m, 6 H, $CH_2CH_2CH_2CH_2C(O)O$ + $CH_2CH_2CH_2CH_2C(O)O$ + $CH_2CH_2CH_2CH_2C(O)O$), 2.66 (d, *J* = 7.2 Hz, 2 H, $CH_2CH{=}CHAr$), 2.73 (t, *J* = 6.2 Hz, 2 H, $CH_2CH_2CH_2CH_2C(O)O$), 3.08 (s, 2 H, $CCH_2Ar$), 6.22−6.32 (m, 1 H, $CH_2CH{=}CHAr$), 6.47 (d, *J* = 15.6 Hz, 1 H, $CH_2CH{=}CHAr$), 7.22−7.42 (m, 10 H, Ar*H*) ppm; $^{13}$C-NMR (100 MHz, $CDCl_3$) δ 23.0 ($CH_2CH_2CH_2CH_2C(O)O$), 23.2 ($CH_2CH_2CH_2CH_2C(O)O$), 36.1 ($CH_2CH_2CH_2CH_2C(O)O$), 37.3 ($CH_2CH_2CH_2CH_2C(O)O$), 41.6 ($CH_2CH{=}CHAr$), 45.2 ($CCH_2Ar$), 85.0 ($CCH_2Ar$), 123.8 ($CH_2CH{=}CHAr$), 126.2 (Ar*C*H), 126.8 (Ar*C*H), 127.5 (Ar*C*H), 128.2 (Ar*C*H), 128.6 (Ar*C*H), 130.8 (Ar*C*H), 134.2 ($CH_2CH{=}CHAr$), 136.0 (Ar*C*), 136.9 (Ar*C*), 174.5 (*C*(O)O) ppm; IR $\nu_{max}$ (thin film/cm$^{-1}$): 3027, 2934, 2864, 1714 (C=O), 1495, 1449, 1285, 1163, 1016; HRMS cald for $C_{22}H_{24}O_2Na$ $[M+Na]^+$: 343.1667, found 343.1674.

**(*E*)-7-Benzyl-7-(3-(2-fluorophenyl)allyl)oxepan-2-one (1m)**

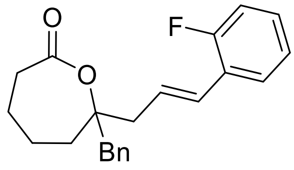


Prepared according to general procedure D using lactone **1k** (0.15 g, 0.60 mmol), 2-fluorostyrene (210 μL, 1.80 mmol) and Hoveyda-Grubbs 2nd generation catalyst (4 mg, 5.94

μmol) in $CH_2Cl_2$ (1.5 mL). The crude mixture was purified by silica gel column chromatography (hexane/EtOAc, 100:0 to 80:20). The title product was obtained as a dark oil (135 mg, 0.402 mmol, 67%). $^1$H-NMR (400 MHz, $CDCl_3$) δ 1.59−2.03 (m, 6 H, $CH_2CH_2CH_2CH_2C(O)O$ + $CH_2CH_2CH_2CH_2C(O)O$ + $CH_2CH_2CH_2CH_2C(O)O$), 2.68 (d, *J* = 7.2 Hz, 2 H, C*H*$_2$CH=CHAr), 2.71–2.77 (m, 2 H, $CH_2CH_2CH_2CH_2C(O)O$), 3.09 (s, 2 H, CC*H*$_2$Ar), 6.34 (dt, *J* = 16.0, 7.2 Hz, 1 H, $CH_2$C*H*=CHAr), 6.63 (d, *J* = 16.0 Hz, 1 H, $CH_2$CH=C*H*Ar), 7.04 (ddd, *J* = 10.8, 8.2, 1.0 Hz, 1 H, Ar*H*), 7.08−7.14 (m, 1 H, Ar*H*), 7.18−7.35 (m, 6 H, Ar*H*), 7.48 (td, *J* = 8.0, 1.2 Hz, 1 H, Ar*H*) ppm; $^{13}$C-NMR (100 MHz, $CDCl_3$) δ 23.0 ($CH_2CH_2CH_2CH_2C(O)O$), 23.2 ($CH_2CH_2CH_2CH_2C(O)O$), 36.2 ($CH_2CH_2CH_2CH_2C(O)O$), 37.3 ($CH_2CH_2CH_2CH_2C(O)O$), 42.0 (*C*H$_2$CH=CHAr), 45.2 (C*C*H$_2$Ar), 84.9 (*C*CH$_2$Ar), 115.7 (*J* = 21.9 Hz, Ar*C*H), 124.1 (*J* = 2.9 Hz, Ar*C*H), 124.7 (*J* = 11.6 Hz, Ar*C*), 126.5 (*J* = 3.6 Hz, $CH_2$CH=*C*HAr), 126.6 (*J* = 4.4 Hz, $CH_2$*C*H=CHAr), 126.9 (Ar*C*H), 127.1 (*J* = 3.7 Hz, Ar*C*H), 128.3 (Ar*C*H), 128.7 (*J* = 8.1 Hz, Ar*C*H), 130.7 (Ar*C*H), 135.9 (Ar*C*), 159.9 (*J* = 247.2 Hz, Ar*C*F), 174.4 (*C*(O)O) ppm; $^{19}$F-NMR (376 MHz, $CDCl_3$) δ −118.7 (m, Ar*F*) ppm; IR $\nu_{max}$ (thin film/cm$^{-1}$): 3029, 2935, 2865, 1713 (C=O), 1486, 1454, 1285, 1227, 1163, 1102; HRMS cald for $C_{22}H_{23}FO_2Na$ [M+Na]$^+$: 361.1596, found 361.1580.

**Allyl 2-oxocyclohexane-1-carboxylate (S11)[4]**

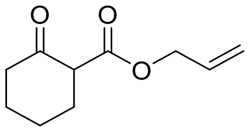

Prepared according to general procedure A using diallyl pimelate[1] (5.00 g, 20.8 mmol) and 60% NaH in mineral oil (0.92 g, 22.9 mmol) in THF (15 mL). The mixture was allowed to cool to room temperature before quenching with saturated aqueous $NH_4Cl$ (75 mL), followed by 1M HCl (40 mL). The crude product was used in the next step without further purification.

**2-Allylcyclohexan-1-one (S12)**[5]

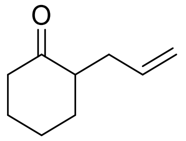

Prepared according to general procedure B using crude ketoester **S11** (20.8 mmol), $Pd_2(dba)_3$ (0.88 g, 0.96 mmol) and $PPh_3$ (1.01 g, 3.80 mmol) in THF (184 mL). The product was distilled from the crude mixture (80 ºC, 7 mmbar) and was obtained as a colourless oil (1.44 g, 10.4 mmol, 50% from diallyl pimelate). $^1$H-NMR (400 MHz, $CDCl_3$) δ 1.29−1.43 (m, 1 H, $CH_2CH_2C\mathit{H}_aH_bCH_2CHC(O)$), 1.57−1.75 (m, 2 H, $CH_2CH_2CH_2C\mathit{H}_aH_bCHC(O)$ + $CH_2C\mathit{H}_aH_bCH_2CH_2CHC(O)$), 1.80−1.93 (m, 1 H, $CH_2CH_2CH_2CH_a\mathit{H}_bCHC(O)$), 1.94−2.20 (m, 3 H, $C\mathit{H}_aH_bCH{=}CH_2$ + $CH_2CH_a\mathit{H}_bCH_2CH_2CHC(O)$ + $CH_2CH_2CH_a\mathit{H}_bCH_2CHC(O)$), 2.25−2.46 (m, 3 H, $C\mathit{H}_2CH_2CH_2CH_2CHC(O)$ + $CH_2CH_2CH_2CH_2C\mathit{H}C(O)$), 2.49−2.60 (m, 1 H, $CH_a\mathit{H}_bCH{=}CH_2$), 4.95−5.10 (m, 2 H, $CH_2CH{=}C\mathit{H}_2$), 5.70−5.86 (m, 1 H, $CH_2C\mathit{H}{=}CH_2$) ppm; $^{13}$C-NMR (100 MHz, $CDCl_3$) δ 24.9 ($CH_2\mathit{C}H_2CH_2CH_2CHC(O)$), 27.9 ($CH_2CH_2CH_2\mathit{C}H_2CHC(O)$), 33.4 ($CH_2CH_2\mathit{C}H_2CH_2CHC(O)$), 33.7 ($\mathit{C}H_2CH{=}CH_2$), 42.1 ($\mathit{C}H_2CH_2CH_2CH_2CHC(O)$), 50.2 ($CH_2CH_2CH_2CH_2\mathit{C}HC(O)$), 116.2 ($CH_2CH{=}\mathit{C}H_2$), 136.5 ($CH_2\mathit{C}H{=}CH_2$), 212.7 ($\mathit{C}(O)$) ppm; IR $\nu_{max}$ (thin film/cm$^{-1}$): 2932, 2860, 1708 (C=O), 1448, 1125, 911; HRMS cald for $C_9H_{14}ONa$ $[M+Na]^+$: 161.0950, found 161.0942.

**7-Allyloxepan-2-one (S13)**

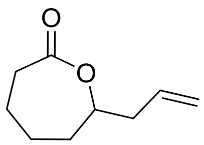

Prepared according to general procedure C using ketone **S12** (1.44 g, 10.4 mmol), *m*CPBA (2.56 g, 11.46 mmol) and $NaHCO_3$ (1.75 g, 20.8 mmol) in $CH_2Cl_2$ (23 mL). The resulting crude mixture was purified by silica gel column chromatography (hexane/EtOAc, 80:20). The title product was obtained as a pale yellow oil (0.85 g, 5.51 mmol, 53%). $^1$H-NMR (400

MHz, $CDCl_3$) δ 1.51−1.67 (m, 3 H, C$H_a$$H_b$$CH_2CH_2CH_2$C(O)O + $CH_2$C$H_a$$H_b$$CH_2CH_2$C(O)O + $CH_2CH_2$C$H_a$$H_b$$CH_2$C(O)O), 1.88−2.03 (m, 3 H, C$H_a$$H_b$$CH_2CH_2CH_2$C(O)O + $CH_2$C$H_a$$H_b$$CH_2CH_2$C(O)O + $CH_2CH_2$C$H_a$$H_b$$CH_2$C(O)O), 2.29−2.38 (m, 1 H, OCHC$H_a$$H_b$CH=$CH_2$), 2.44−2.54 (m, 1 H, OCHC$H_a$$H_b$CH=$CH_2$), 2.55−2.73 (m, 2 H, $CH_2CH_2CH_2$C$H_2$C(O)O), 4.25−4.34 (m, 1 H, OC*H*$CH_2$CH=$CH_2$), 5.08−5.18 (m, 2 H, OCH$CH_2$CH=C$H_2$), 5.77–5.91 (m, 1 H, OCH$CH_2$C*H*=$CH_2$) ppm; $^{13}$C-NMR (100 MHz, $CDCl_3$) δ 22.9 ($CH_2CH_2$*C*$H_2CH_2$C(O)O), 28.2 ($CH_2$*C*$H_2CH_2CH_2$C(O)O), 33.8 ($CH_2CH_2CH_2$*C*$H_2$C(O)O), 34.9 (*C*$H_2CH_2CH_2CH_2$C(O)O), 40.6 (OCH*C*$H_2$CH=$CH_2$), 79.9 (O*C*H$CH_2$CH=$CH_2$), 118.3 (OCH$CH_2$CH=*C*$H_2$), 133.4 (OCH$CH_2$*C*H=$CH_2$), 175.6 (*C*(O)O) ppm; IR $\nu_{max}$ (thin film/cm$^{-1}$): 2934, 2862, 1725 (C=O), 1174, 1013; HRMS calcd for $C_9H_{14}O_2Na$ $[M+Na]^+$: 177.0891, found 177.0891.

**7-Cinnamyloxepan-2-one (1n)**

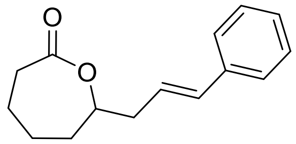

Prepared according to general procedure D using lactone **S13** (0.15 g, 0.97 mmol), styrene (300 μL, 2.92 mmol) and Hoveyda-Grubbs 2$^{nd}$ generation catalyst (6 mg, 9.00 μmol) in $CH_2Cl_2$ (2.4 mL). The crude mixture was purified by silica gel column chromatography (hexane/EtOAc, 100:0 to 80:20). The title product was obtained as a pale yellow oil (85 mg, 0.369 mmol, 38%). $^1$H-NMR (400 MHz, $CDCl_3$) δ 1.54−1.71 (m, 3 H, C$H_a$$H_b$$CH_2CH_2CH_2$C(O)O + $CH_2$C$H_a$$H_b$$CH_2CH_2$C(O)O + $CH_2CH_2$C$H_a$$H_b$$CH_2$C(O)O), 1.90−2.06 (m, 3 H, C$H_a$$H_b$$CH_2CH_2CH_2$C(O)O + $CH_2$C$H_a$$H_b$$CH_2CH_2$C(O)O + $CH_2CH_2$C$H_a$$H_b$$CH_2$C(O)O), 2.46−2.55 (m, 1 H, CHC$H_a$$H_b$CH=CHAr), 2.57−2.74 (m, 3 H, $CH_2CH_2CH_2$C$H_2$C(O)O + CHC$H_a$$H_b$CH=CHAr), 4.34−4.41 (m, 1 H, OC*H*$CH_2$CH=CHAr), 6.22−6.31 (m, 1 H, CH$CH_2$C*H*=CHAr), 6.48 (d, *J* = 15.8, 1 H, CH$CH_2$CH=C*H*Ar), 7.23 (t, *J*

= 7.2 Hz, 1 H, Ar*H*), 7.31 (t, *J* = 7.2 Hz, 2 H, Ar*H*), 7.37 (d, *J* = 7.2 Hz, 2 H, Ar*H*) ppm; $^{13}$C-NMR (100 MHz, $CDCl_3$) δ 22.9 ($CH_2CH_2\mathit{C}H_2CH_2C(O)O$), 28.3 ($CH_2\mathit{C}H_2CH_2CH_2C(O)O$), 33.9 ($\mathit{C}H_2CH_2CH_2CH_2C(O)O$), 34.9 ($CH_2CH_2CH_2\mathit{C}H_2C(O)O$), 39.9 ($CH\mathit{C}H_2CH{=}CHAr$), 80.2 ($O\mathit{C}HCH_2CH{=}CHAr$), 124.9 ($CHCH_2\mathit{C}H{=}CHAr$), 126.1 (Ar*C*H), 127.4 (Ar*C*H), 128.5 (Ar*C*H), 133.3 ($CHCH_2CH{=}\mathit{C}HAr$), 137.0 (Ar*C*), 175.6 (*C*(O)O) ppm; IR $\nu_{max}$ (thin film/cm$^{-1}$): 2933, 2860, 1725 (C=O), 1447, 1281, 1255, 1173, 1013; HRMS cald for $C_{15}H_{18}O_2Na$ $[M+Na]^+$: 253.1193, found 253.1204.

**(*E*)-7-(3-(2-Fluorophenyl)allyl)oxepan-2-one (1o)**

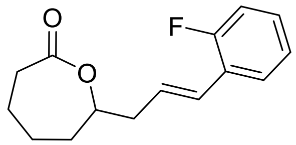

Prepared according to general procedure D using lactone **S13** (0.15 g, 0.97 mmol), 2-fluorostyrene (350 μL, 2.92 mmol) and Hoveyda-Grubbs 2$^{nd}$ generation catalyst (6 mg, 9.00 μmol) in $CH_2Cl_2$ (2.4 mL). The crude mixture was purified by silica gel column chromatography (hexane/EtOAc, 100:0 to 80:20). The title product was obtained as a pale yellow oil (170 mg, 0.669 mmol, 69%). $^1$H-NMR (400 MHz, $CDCl_3$) δ 1.55−1.71 (m, 3 H, $C\mathit{H}_aH_bCH_2CH_2CH_2C(O)O$ + $CH_2C\mathit{H}_aH_bCH_2CH_2C(O)O$ + $CH_2CH_2C\mathit{H}_aH_bCH_2C(O)O$), 1.90−2.06 (m, 3 H, $CH_a\mathit{H}_bCH_2CH_2CH_2C(O)O$ + $CH_2CH_a\mathit{H}_bCH_2CH_2C(O)O$ + $CH_2CH_2CH_a\mathit{H}_bCH_2C(O)O$), 2.49−2.75 (m, 4 H, $CH_2CH_2CH_2C\mathit{H}_2C(O)O$ + $CHC\mathit{H}_2CH{=}CHAr$), 4.35−4.43 (m, 1 H, $C\mathit{H}CH_2CH{=}CHAr$), 6.29−6.39 (m, 1 H, $CHCH_2C\mathit{H}{=}CHAr$), 6.65 (d, *J* = 16.0 Hz, 1 H, $CHCH_2CH{=}C\mathit{H}Ar$), 6.98−7.13 (m, 2 H, Ar*H*), 7.16−7.24 (m, 1 H, Ar*H*), 7.46 (t, *J* = 7.6 Hz, 1 H, Ar*H*) ppm; $^{13}$C-NMR (100 MHz, $CDCl_3$) δ 22.9 ($CH_2CH_2\mathit{C}H_2CH_2C(O)O$), 28.3 ($CH_2\mathit{C}H_2CH_2CH_2C(O)O$), 34.0 ($\mathit{C}H_2CH_2CH_2CH_2C(O)O$), 34.9 ($CH_2CH_2CH_2\mathit{C}H_2C(O)O$), 40.2 ($CH\mathit{C}H_2CH{=}CHAr$), 80.1 ($O\mathit{C}HCH_2CH{=}CHAr$), 115.6 (*J* = 22.6 Hz, Ar*C*H), 124.1 (*J* = 2.9 Hz, Ar*C*H), 124.7 (*J* = 11.7

Hz, Ar*C*), 125.5 ($J$ = 4.4 Hz, CHCH$_2$CH=*C*HAr), 127.1 ($J$ = 3.7 Hz, Ar*C*H), 127.6 ($J$ = 4.4 Hz, CHCH$_2$*C*H=CHAr), 128.6 ($J$ = 8.0 Hz, Ar*C*H), 159.9 ($J$ = 247.2 Hz, Ar*C*F), 175.6 (*C*(O)O) ppm; $^{19}$F-NMR (376 MHz, CDCl$_3$) δ −118.7 (m, Ar*F*) ppm; IR $\nu_{max}$ (thin film/cm$^{-1}$): 2935, 2862, 1727 (C=O), 1486, 1456, 1228, 1013; HRMS cald for $C_{15}H_{17}FO_2Na$ [M+Na]$^+$: 271.1104, found 271.1110.

**General Procedure F: $SmI_2$-$H_2O$-Mediated Radical Cyclization + DMP oxidation**

***rac*-(1*S*,6*R*,8*S*)-8-Benzyl-6-methyl-9-oxabicyclo[4.2.1]nonan-1-ol (3a)**

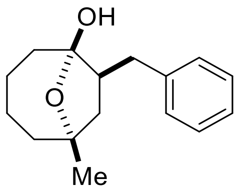


To a flask charged with 0.1 M $SmI_2$ in THF (13.1 mL, 1.31 mmol) was added degassed distilled $H_2O$ (2.4 mL, 131 mmol). The resulting deep red solution was stirred for 5 min before adding dropwise a solution of lactone **1a** (40 mg, 0.164 mmol) in THF (0.4 mL). The mixture was stirred at room temperature until it decolourised. A saturated solution of Rochelle's salt was added and the mixture was extracted with $Et_2O$ (3 × 15 mL) and the combined organic layers were washed with brine, dried ($MgSO_4$) and concentrated under vacuum. The resulting crude mixture was dissolved in dry $CH_2Cl_2$ (1.7 mL) and Dess-Martin periodinane (87 mg, 0.205 mmol) was added in one portion. The resulting mixture was stirred at room temperature for 5 h before being quenched with a mixture of saturated aqueous solution $Na_2S_2O_3$/$NaHCO_3$ (1:1, 20 mL) and extracted with $CH_2Cl_2$ (3 × 10 mL). The combined organic layers were dried ($MgSO_4$) and concentrated under vacuum. The resulting crude mixture was purified by silica gel column chromatography (hexane/EtOAc, 90:10). The title product was obtained as a 75:25 mixture of diastereomers (25 mg, 0.812 mmol, 62%). The major diastereomer could be isolated and was obtained as colorless crystals: mp

($CH_2Cl_2$) 89–92 °C. $^1$H-NMR (400 MHz, $CDCl_3$) δ 1.27 (s, 3 H, C$H_3$), 1.50–1.93 (m, 9 H, $CH_2CH_2CH_2CH_2COH$ + $CH_2CH_2CH_2CH_2COH$ + $CH_2CH_2CH_2CH_2COH$ + $CH_2CH_2CH_2CH_aH_bCOH$ + $CH_2CHCH_2Ar$), 2.12–2.22 (m, 1 H, $CH_2CH_2CH_2CH_aH_bCOH$), 2.27 (qd, $J$ = 11.9, 3.3 Hz, 1 H, $CHCH_2Ar$), 2.56 (t, $J$ = 11.9 Hz, 1 H, $CH_aH_bAr$), 3.01 (dd, $J$ = 11.9, 3.3 Hz, 1 H, $CH_aH_bAr$), 7.17–7.24 (m, 3 H, Ar$H$), 7.25–7.34 (m, 2 H, Ar$H$) ppm; $^{13}$C-NMR (100 MHz, $CDCl_3$) δ 22.4 ($CH_2CH_2CH_2CH_2COH$), 25.0 ($CH_2CH_2CH_2CH_2COH$), 31.1 ($CH_3$), 36.0 ($CH_2Ar$), 37.8 ($CH_2CH_2CH_2CH_2COH$), 41.9 ($CH_2CH_2CH_2CH_2COH$), 42.9 ($CH_2CHCH_2Ar$), 51.9 ($CHCH_2Ar$), 78.6 ($CH_3CO$), 107.7 (O$C$OH), 126.0 (Ar$C$H), 128.4 (Ar-$C$H), 128.6 (Ar$C$H), 140.8 (Ar$C$) ppm; IR $\nu_{max}$ (thin film/cm$^{-1}$): 3382 (O-H), 2926, 2856, 1708, 1453, 1117, 1088, 943; HRMS calcd for $C_{16}H_{21}O_2$ [M–H]$^-$: 245.1542, found 245.1533.

**_rac_-(1_S_,6_R_,8_S_)-8-(2-Chlorobenzyl)-6-methyl-9-oxabicyclo[4.2.1]nonan-1-ol (3b)**

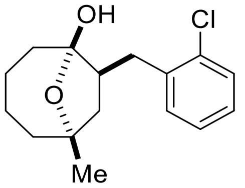


Prepared according to general procedure F using lactone **1b** (44.6 mg, 0.160 mmol), 0.1 M $SmI_2$ in THF (12.8 mL, 1.28 mmol) and $H_2O$ (2.3 mL, 128 mmol). Oxidation of the crude was carried out using Dess-Martin periodinane (102 mg, 0.24 mmol) in dry $CH_2Cl_2$ (2 mL). The crude mixture was purified by silica gel column chromatography (hexane/EtOAc, 90:10 to 50:50). The title product was obtained as a 89:11 mixture of diastereomers (32 mg, 0.114 mmol, 71%). The major diastereomer could be isolated and was obtained as colorless crystals: mp ($CH_2Cl_2$) 105–109 °C. $^1$H-NMR (400 MHz, $CDCl_3$) δ 1.27 (s, 3 H, C$H_3$), 1.51–1.86 (m, 8 H, $CH_2CH_2CH_2CH_2COH$ + $CH_2CH_2CH_2CH_2COH$ + $CH_2CH_2CH_2CH_2COH$ + $CH_2CH_2CH_2CH_aH_bCOH$ + $CH_aH_bCHCH_2Ar$), 1.90 (t, $J$ = 10.0 Hz, 1 H, $CH_aH_bCHCH_2Ar$), 2.15–2.22 (m, 1 H, $CH_2CH_2CH_2CH_aH_bCOH$), 2.29–2.39 (m, 1 H, $CHCH_2Ar$), 2.75 (dd, $J$ =

10.4, 8.8 Hz, 1 H, C$H_a$$H_b$Ar), 3.12 (dd, $J$ = 10.4, 3.0 Hz, 1 H, C$H_a$$H_b$Ar), 7.16 (td, $J$ = 6.0, 1.4 Hz, 1 H, Ar*H*), 7.20 (td, $J$ = 6.0, 1.4 Hz, 1 H, Ar*H*), 7.24 (dd, $J$ = 6.0, 1.4 Hz, 1 H, Ar*H*), 7.35 (dd, $J$ = 6.0, 1.4 Hz, 1 H, Ar*H*) ppm; $^{13}$C-NMR (100 MHz, $CDCl_3$) δ 22.4 ($CH_2CH_2$*C*$H_2CH_2$COH), 25.0 ($CH_2$*C*$H_2CH_2CH_2$COH), 31.1 (*C*$H_3$), 33.1 (*C*$H_2$Ar), 37.5 ($CH_2CH_2CH_2$*C*$H_2$COH), 41.7 (*C*$H_2CH_2CH_2CH_2$COH), 42.2 (*C*$H_2$CH$CH_2$Ar), 50.3 (*C*H$CH_2$Ar), 78.6 ($CH_3$*C*O), 107.7 (O*C*OH), 126.8 (Ar*C*H), 127.6 (Ar*C*H), 129.6 (Ar*C*H), 130.6 (Ar*C*H), 133.8 (Ar*C*Cl), 138.2 (Ar*C*) ppm; IR $\nu_{max}$ (thin film/cm$^{-1}$): 3356 (O-H), 2926, 1707, 1441, 1370, 1274, 1144, 1122, 1108, 1062; HRMS calcd for $C_{16}H_{20}O_2Cl$ $[M–H]^-$: 279.1152, found 279.1152.

**_rac_-(1*S*,6*R*,8*S*)-6-Methyl-8-(4-(trifluoromethyl)benzyl)-9-oxabicyclo[4.2.1]nonan-1-ol (3c)**

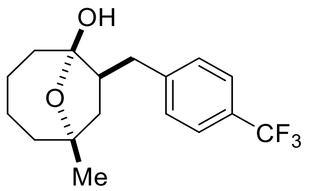


Prepared according to general procedure F using lactone **1c** (50.0 mg, 0.160 mmol), 0.1 M $SmI_2$ in THF (12.8 mL, 1.28 mmol) and $H_2O$ (2.3 mL, 128 mmol). Oxidation of the crude was carried out using Dess-Martin periodinane (102 mg, 0.24 mmol) in dry $CH_2Cl_2$ (2 mL). The crude mixture was purified by silica gel column chromatography (hexane/EtOAc, 90:10 to $CHCl_3$/$Et_2O$ 70:30). The title product was obtained as a 83:17 mixture of diastereomers, colorless crystals (40 mg, 0.128 mmol, 80%). For the mixture of diastereomers: mp ($CH_2Cl_2$) 102–106 ºC. Data for the major diastereomer: $^1$H-NMR (400 MHz, $CDCl_3$) δ 1.28 (s, 3 H, C$H_3$), 1.51–1.89 (m, 9 H, C$H_2CH_2CH_2CH_2$COH + $CH_2$C$H_2CH_2CH_2$COH + $CH_2CH_2$C$H_2CH_2$COH + $CH_2CH_2CH_2$C$H_a$$H_b$COH + C$H_2$CH$CH_2$Ar), 2.10–2.21 (m, 1 H, $CH_2CH_2CH_2$C$H_a$$H_b$COH), 2.22–2.34 (m, 1 H, C*H*$CH_2$Ar), 2.61 (t, $J$ = 13.0 Hz, 1 H,

C$H_a$H$_b$Ar), 3.06 (dd, $J$ = 13.0, 3.4 Hz, 1 H, CH$_a$$H_b$Ar), 7.32 (d, $J$ = 8.0 Hz, 2 H, Ar$H$), 7.55 (d, $J$ = 8.0 Hz, 2 H, Ar$H$) ppm; $^{13}$C-NMR (100 MHz, $CDCl_3$) δ 22.4 ($CH_2CH_2$$C$$H_2CH_2$COH), 25.0 ($CH_2$$C$$H_2CH_2CH_2$COH), 31.1 ($C$$H_3$), 35.8 ($C$$H_2$Ar), 37.9 ($CH_2CH_2CH_2$$C$$H_2$COH), 41.8 ($C$$H_2CH_2CH_2CH_2$COH), 42.7 ($C$$H_2$CH$CH_2$Ar), 51.5 ($C$H$CH_2$Ar), 78.8 ($CH_3$$C$O), 107.6 (O$C$OH), 125.4 (q, $J$ = 3.9 Hz, Ar$C$H), 128.9 (Ar$C$H), 128.8 (q, $J$ = 38.3 Hz, Ar$C$$CF_3$), 144.8 (Ar$C$) ppm, Ar$C$$F_3$ not observed; $^{19}$F-NMR (376 MHz, $CDCl_3$) δ –62.3 (s, C$F_3$) ppm; IR $\nu_{max}$ (thin film/cm$^{-1}$): 3349 (O-H), 2931, 2859, 1709, 1618, 1324, 1160, 1115, 1065; HRMS calcd for $C_{17}H_{20}O_2F_3$ [M–H]$^-$: 313.1415, found 313.1401.

**_rac_-(1_S_,6_R_,8_S_)-8-(4-Bromobenzyl)-6-methyl-9-oxabicyclo[4.2.1]nonan-1-ol (3d)**

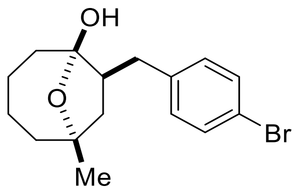


Prepared according to general procedure F using lactone **1d** (51.7 mg, 0.160 mmol), 0.1 M $SmI_2$ in THF (12.8 mL, 1.28 mmol) and $H_2O$ (2.3 mL, 128 mmol). Oxidation of the crude mixture was carried out using Dess-Martin periodinane (102 mg, 0.24 mmol) in dry $CH_2Cl_2$ (2 mL). The crude mixture was purified by silica gel column chromatography (hexane/EtOAc, 85:15 to $CHCl_3$/$Et_2O$ 70:30). The title product was obtained as a 80:20 mixture of diastereomers and as colorless crystals (33 mg, 0.100 mmol, 63%). For the mixture of diastereomers: mp ($CH_2Cl_2$) 109–112 ºC. Data for the major diastereomer: $^{1}$H-NMR (400 MHz, $CDCl_3$) δ 1.27 (s, 3 H, C$H_3$), 1.49–1.91 (m, 9 H, C$H_2$$CH_2CH_2CH_2$COH + $CH_2$C$H_2$$CH_2CH_2$COH + $CH_2CH_2$C$H_2$$CH_2$COH + $CH_2CH_2CH_2$C$H_a$H$_b$COH + C$H_2$CH$CH_2$Ar), 2.07–2.31 (m, 2 H, $CH_2CH_2CH_2$CH$_a$$H_b$COH + C$H$$CH_2$Ar), 2.45–2.56 (m, 1 H, C$H_a$H$_b$Ar), 2.95 (dd, $J$ = 13.2, 3.6 Hz, 1 H, CH$_a$$H_b$Ar), 7.08 (d, $J$ = 8.4 Hz, 2 H, Ar$H$), 7.41 (d, $J$ = 8.4 Hz, 2 H, Ar$H$) ppm; $^{13}$C-NMR (100 MHz, $CDCl_3$) δ 22.4 ($CH_2CH_2$$C$$H_2CH_2$COH), 25.0

($CH_2CH_2CH_2CH_2COH$), 31.1 (*C*$H_3$), 35.4 (*C*$H_2$Ar), 37.8 ($CH_2CH_2CH_2$*C*$H_2$COH), 41.8 (*C*$H_2CH_2CH_2CH_2$COH), 42.8 (*C*$H_2CHCH_2$Ar), 51.6 (*C*$HCH_2$Ar), 78.7 ($CH_3$*C*O), 107.6 (O*C*OH), 119.8 (Ar*C*Br), 130.3 (Ar*C*H), 131.5 (Ar*C*H), 139.7 (Ar*C*) ppm; IR $\nu_{max}$ (thin film/cm$^{-1}$): 3339 (O-H), 2925, 2856, 1707, 1486, 1371, 1285; HRMS calcd for $C_{16}H_{20}O_2Br$ $[M–H]^-$: 323.0647, found 323.0636.

***rac*-(1*S*,6*R*,8*S*)-8-(2-Fluorobenzyl)-6-methyl-9-oxabicyclo[4.2.1]nonan-1-ol (3e)**

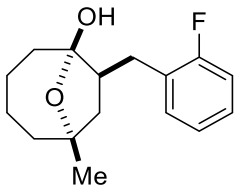


Prepared according to general procedure F using lactone **1e** (80 mg, 0.305 mmol), 0.1 M $SmI_2$ in THF (24.4 mL, 2.44 mmol) and $H_2O$ (4.4 mL, 244 mmol). Oxidation of the crude mixture was carried out using Dess-Martin periodinane (194 mg, 0.458 mmol) in dry $CH_2Cl_2$ (4.4 mL). The crude mixture was purified by silica gel column chromatography (hexane/EtOAc, 90:10 to 85:15). The title product was obtained as a 88:12 mixture of diastereomers (60 mg, 0.226 mmol, 74%). The major diastereomer could be isolated and was obtained as a white solid: mp ($CH_2Cl_2$) 90–92 ºC. $^1$H-NMR (400 MHz, $CDCl_3$) δ 1.27 (s, 3 H, C*H*$_3$), 1.51–1.90 (m, 9 H, C*H*$_2CH_2CH_2CH_2$COH + $CH_2$C*H*$_2CH_2CH_2$COH + $CH_2CH_2$C*H*$_2CH_2$COH + $CH_2CH_2CH_2$C*H*$_a$H$_b$COH + C*H*$_2CHCH_2$Ar), 2.13–2.22 (m, 1 H, $CH_2CH_2CH_2CH_a$*H*$_b$COH), 2.23–2.36 (m, 2 H, $CH_2$C*H*$CH_2$Ar + $CH_2CH_2CH_2CH_2$CO*H*), 2.65–2.73 (m, 1 H, $CH_2CH$C*H*$_a$H$_b$Ar), 2.96 (dd, *J* = 13.2, 3.6 Hz, 1 H, $CH_2CHCH_a$*H*$_b$Ar), 7.00–7.11 (m, 2 H, Ar*H*), 7.12–7.25 (m, 2 H, Ar*H*) ppm; $^{13}$C-NMR (100 MHz, $CDCl_3$) δ 22.3 ($CH_2CH_2$*C*$H_2CH_2$COH), 25.0 ($CH_2$*C*$H_2CH_2CH_2$COH), 28.7 (d, *J* = 1.9 Hz, $CH_2CH$*C*$H_2$Ar), 31.1 (C*H*$_3$), 37.6 ($CH_2CH_2CH_2$*C*$H_2$COH), 41.7 (*C*$H_2CH_2CH_2CH_2$COH), 42.4 (*C*$H_2CHCH_2$Ar), 50.8 ($CH_2$*C*$HCH_2$Ar), 78.5 ($CH_3$*C*O), 107.6 (O*C*OH), 115.3 (d, *J* = 22.4

Hz, Ar*C*H), 124.0 (d, *J* = 3.9 Hz, Ar*C*H), 127.5 (d, *J* = 15.5 Hz, Ar*C*), 127.8 (d, *J* = 8.8 Hz, Ar*C*H), 130.8 (d, *J* = 4.9 Hz, Ar*C*H), 161.0 (d, *J* = 243.1 Hz, Ar*C*F) ppm; $^{19}$F-NMR (376 MHz, $CDCl_3$) δ −118.3 (m, Ar*F*) ppm; IR $\nu_{max}$ (thin film/cm$^{-1}$): 3389 (O-H), 2928, 2860, 1492, 1455, 1229, 1118, 1089; HRMS calcd for $C_{16}H_{21}O_2FNa$ [M+Na]$^+$: 287.1418, found 287.1423.

**_rac_-(1*S*,6*R*,8*S*)-6-Methyl-8-(4-methylbenzyl)-9-oxabicyclo[4.2.1]nonan-1-ol (3f)**

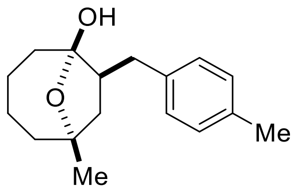


Prepared according to general procedure F using lactone **1f** (41.3 mg, 0.160 mmol), 0.1 M $SmI_2$ in THF (12.8 mL, 1.28 mmol) and $H_2O$ (2.3 mL, 128 mmol). Oxidation of the crude mixture was carried out using Dess-Martin periodinane (102 mg, 0.24 mmol) in dry $CH_2Cl_2$ (2 mL). The crude mixture was purified by silica gel column chromatography (hexane/EtOAc, 90:10). The title product was obtained as a 75:25 mixture of diastereomers (26 mg, 0.099 mmol, 62%). The major diastereomer could be isolated and was obtained as a white solid: mp ($CH_2Cl_2$) 113–116 ºC. $^1$H-NMR (400 MHz, $CDCl_3$) δ 1.27 (s, 3 H, C*H*3), 1.51–1.94 (m, 9 H, C*H*2CH2CH2CH2COH + CH2C*H*2CH2CH2COH + CH2CH2C*H*2CH2COH + CH2CH2CH2C*H*aHbCOH + C*H*2CHCH2Ar), 2.12–2.20 (m, 1 H, CH2CH2CH2CHa*H*bCOH), 2.21–2.29 (m, 1 H, C*H*CH2Ar), 2.33 (s, 3 H, ArC*H*3), 2.51 (t, *J* = 12.6 Hz, 1 H, C*H*aHbAr), 2.96 (dd, *J* = 12.6, 3.6 Hz, 1 H, CHa*H*bAr), 7.11 (bs, 4 H, Ar*H*) ppm; $^{13}$C-NMR (100 MHz, $CDCl_3$) δ 21.0 (Ar*C*H3), 22.4 (CH2CH2*C*H2CH2COH), 25.0 (CH2*C*H2CH2CH2COH), 31.1 (*C*H3CO), 35.5 (*C*H2Ar), 37.8 (CH2CH2CH2*C*H2COH), 41.9 (*C*H2CH2CH2CH2COH), 42.9 (*C*H2CHCH2Ar), 51.9 (*C*HCH2Ar), 78.7 (CH3*C*O), 107.8 (O*C*OH), 128.4 (Ar*C*H), 129.1 (Ar*C*H), 135.5 (Ar*C*CH3), 137.6 (Ar*C*) ppm; IR $\nu_{max}$ (thin film/cm$^{-1}$): 3341 (O-H), 2924,

2852, 1708, 1514, 1455, 1372, 1137, 1116; HRMS calcd for $C_{17}H_{23}O_2$ $[M–H]^-$: 259.1698, found 259.1688.

**rac-(1S,6R,8S)-6-Methyl-8-(3-methylbenzyl)-9-oxabicyclo[4.2.1]nonan-1-ol (3g)**

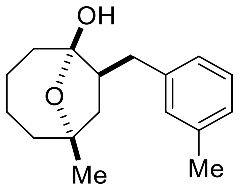


Prepared according to general procedure F using lactone **1g** (41.3 mg, 0.160 mmol), 0.1 M $SmI_2$ in THF (12.8 mL, 1.28 mmol) and $H_2O$ (2.3 mL, 128 mmol). Oxidation of the crude mixture was carried out using Dess-Martin periodinane (102 mg, 0.24 mmol) in dry $CH_2Cl_2$ (2 mL). The crude mixture was purified by silica gel column chromatography (hexane/EtOAc, 90:10). The title product was obtained as a 75:25 mixture of diastereomers (30 mg, 0.115 mmol, 72%). The major diastereomer could be isolated and was obtained as a pale yellow oil. $^1$H-NMR (400 MHz, $CDCl_3$) δ 1.27 (s, 3 H, $CH_3$), 1.50–1.92 (m, 9 H, $CH_2CH_2CH_2CH_2COH$ + $CH_2CH_2CH_2CH_2COH$ + $CH_2CH_2CH_2CH_2COH$ + $CH_2CH_2CH_2CH_aH_bCOH$ + $CH_2CHCH_2Ar$), 2.12–2.20 (m, 1 H, $CH_2CH_2CH_2CH_aH_bCOH$), 2.21–2.38 (s, 4 H, $CHCH_2Ar$ + $ArCH_3$), 2.51 (t, $J$ = 12.7 Hz, 1 H, $CH_aH_bAr$), 2.97 (dd, $J$ = 12.7, 3.6 Hz, 1 H, $CH_aH_bAr$), 6.89–7.08 (m, 3 H, Ar*H*) 7.19 (t, $J$ = 7.6 Hz, 1 H, Ar*H*) ppm; $^{13}$C-NMR (100 MHz, $CDCl_3$) δ 21.4 ($ArCH_3$), 22.4 ($CH_2CH_2CH_2CH_2COH$), 25.0 ($CH_2CH_2CH_2CH_2COH$), 31.1 ($CH_3CO$), 35.9 ($CH_2Ar$), 37.8 ($CH_2CH_2CH_2CH_2COH$), 41.8 ($CH_2CH_2CH_2CH_2COH$), 42.9 ($CH_2CHCH_2Ar$), 51.8 ($CHCH_2Ar$), 78.7 ($CH_3CO$), 107.8 (OCOH), 125.5 (ArCH), 126.8 (ArCH), 128.3 (ArCH), 129.4 (ArCH), 138.0 ($ArCCH_3$), 140.7 (ArC) ppm; IR $\nu_{max}$ (thin film/cm$^{-1}$): 3387 (O-H), 2923, 2855, 1707, 1608, 1448, 1369, 1116, 1087, 1059; HRMS calcd for $C_{17}H_{23}O_2$ $[M–H]^-$: 259.1698, found 259.1691.

***rac*-(1*S*,6*R*,8*S*)-6-Methyl-8-(naphthalen-2-ylmethyl)-9-oxabicyclo[4.2.1]nonan-1-ol (3h)**

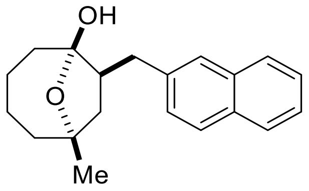


Prepared according to general procedure F using lactone **1h** (47.1 mg, 0.160 mmol), 0.1 M $SmI_2$ in THF (12.8 mL, 1.28 mmol) and $H_2O$ (2.3 mL, 128 mmol). Oxidation of the crude mixture was carried out using Dess-Martin periodinane (102 mg, 0.24 mmol) in dry $CH_2Cl_2$ (2 mL). The crude mixture was purified by silica gel column chromatography (hexane/EtOAc, 90:10). The title product was obtained as a 82:18 mixture of diastereomers (44 mg, 0.145 mmol, 93%). The major diastereomer could be isolated and was obtained as colourless crystals: mp ($CH_2Cl_2$) 105–110 ºC. $^1$H-NMR (400 MHz, $CDCl_3$) δ 1.26 (s, 3 H, C$H_3$), 1.51–1.92 (m, 9 H, C$H_2$CH$_2$CH$_2$CH$_2$COH + CH$_2$C$H_2$CH$_2$CH$_2$COH + CH$_2$CH$_2$C$H_2$CH$_2$COH + CH$_2$CH$_2$CH$_2$C$H_a$H$_b$COH + C$H_2$CHCH$_2$Ar), 2.16–2.28 (m, 1 H, CH$_2$CH$_2$CH$_2$CH$_a$$H_b$COH), 2.29–2.46 (m, 1 H, C*H*CH$_2$Ar), 2.72 (t, *J* = 12.3 Hz, 1 H, C$H_a$H$_b$Ar), 3.17 (dd, *J* = 12.3, 3.4 Hz, 1 H, CH$_a$$H_b$Ar), 7.33–7.51 (m, 3 H, Ar*H*), 7.65 (s, 1 H, Ar*H*), 7.75–7.86 (m, 3 H, Ar*H*) ppm; $^{13}$C-NMR (100 MHz, $CDCl_3$) δ 22.5 (CH$_2$CH$_2$*C*H$_2$CH$_2$COH), 25.0 (CH$_2$*C*H$_2$CH$_2$CH$_2$COH), 31.1 (*C*H$_3$), 36.2 (*C*H$_2$Ar), 37.9 (CH$_2$CH$_2$CH$_2$*C*H$_2$COH), 41.8 (*C*H$_2$CH$_2$CH$_2$CH$_2$COH), 42.9 (*C*H$_2$CHCH$_2$Ar), 51.8 (*C*HCH$_2$Ar), 78.8 (CH$_3$*C*O), 107.8 (O*C*OH), 125.3 (Ar*C*H), 126.0 (Ar*C*H), 126.7 (Ar*C*H), 127.2 (Ar*C*H), 127.4 (Ar*C*H), 127.6 (Ar*C*H), 128.0 (Ar*C*H), 132.0 (Ar*C*), 133.6 (Ar*C*), 138.3 (Ar*C*CH$_2$) ppm; IR $\nu_{max}$ (thin film/cm$^{-1}$): 3313 (O-H), 2919, 2855, 1707, 1372, 1285, 1134; HRMS calcd for $C_{20}H_{24}O_2Na$ [M+Na]$^+$: 319.1674, found 319.1678.

***rac*-(1*S*,6*R*,8*S*)-6,8-Dimethyl-9-oxabicyclo[4.2.1]nonan-1-ol (3i)**

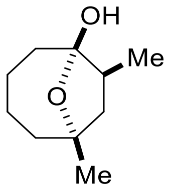


Prepared according to general procedure F using lactone **1i** (100 mg, 0.594 mmol), 0.1 M $SmI_2$ in THF (48 mL, 4.76 mmol) and $H_2O$ (8.6 mL, 476 mmol). Oxidation of the crude mixture was carried out using Dess-Martin periodinane (378 mg, 0.891 mmol) in dry $CH_2Cl_2$ (13 mL). The crude mixture was then purified by silica gel column chromatography (hexane/EtOAc, 90:10 to 80:20). The title product was obtained as a 61:39 mixture of diastereomers (21 mg, 0.123 mmol, 21%). The major diastereomer could be isolated and was obtained as a pale yellow oil. $^1$H-NMR (400 MHz, $CDCl_3$) δ 1.08 (d, *J* = 6.4 Hz, 3 H, $CH_2CHC\mathit{H}_3$), 1.32 (s, 3 H, $C\mathit{H}_3CO$), 1.46–1.83 (m, 8 H, $CH_2CH_2C\mathit{H}_2CH_2COH$ + $CH_2C\mathit{H}_2CH_2CH_2COH$ + $CH_2CH_2CH_2C\mathit{H}_aH_bCOH$ + $C\mathit{H}_2CH_2CH_2CH_2COH$ + $C\mathit{H}_aH_bCHCH_3$), 1.98–2.20 (m, 3 H, $CH_2CH_2CH_2CH_a\mathit{H}_bCOH$ + $CH_2C\mathit{H}CH_3$ + $CH_a\mathit{H}_bCHCH_3$), 2.36 (bs, 1 H, O*H*) ppm; $^{13}$C-NMR (100 MHz, $CDCl_3$) δ 13.5 ($CH_2CH\mathit{C}H_3$), 22.3 ($CH_2CH_2\mathit{C}H_2CH_2COH$), 25.0 ($CH_2\mathit{C}H_2CH_2CH_2COH$), 31.2 ($\mathit{C}H_3CO$), 37.0 ($CH_2CH_2CH_2\mathit{C}H_2COH$), 41.9 ($\mathit{C}H_2CH_2CH_2CH_2COH$), 44.3 ($CH_2\mathit{C}HCH_3$), 44.7 ($\mathit{C}H_2CHCH_3$), 78.3 ($CH_3\mathit{C}O$), 108.2 ($CH_2CH_2CH_2CH_2\mathit{C}OH$) ppm; IR $\nu_{max}$ (thin film/cm$^{-1}$): 3395 (O-H), 2969, 2927, 2858, 1699, 1453, 1372, 1151, 1120, 1091, 1064; HRMS calcd for $C_{10}H_{18}O_2Na$ $[M+Na]^+$: 193.1199, found 193.1204.

***rac*-(6*R*,8*S*)-8-Benzyl-6-hydroxy-2,2,6-trimethylcyclooctan-1-one (3j)**

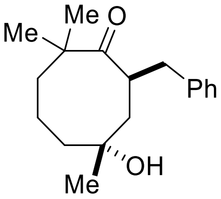

Prepared according to general procedure F using lactone **1j** (50.0 mg, 0.184 mmol), 0.1 M $SmI_2$ in THF (15.0 mL, 1.47 mmol) and $H_2O$ (2.6 mL, 147 mmol). Oxidation of the crude mixture was carried out using Dess-Martin periodinane (117 mg, 0.276 mmol) in dry $CH_2Cl_2$ (2.3 mL). The crude mixture was then purified by silica gel column chromatography (hexane/EtOAc, 95:5 to 80:20). The title product was obtained as a 82:18 mixture of diastereomers (26 mg, 0.094 mmol, 51%). The major diastereomer could be isolated and was obtained as a pale yellow oil. $^1$H-NMR (400 MHz, $CDCl_3$) δ 0.47 (s, 3 H, $(CH_3)_a(CH_3)_bC$), 1.00 (s, 3 H, $(CH_3)_a(CH_3)_bC$), 1.20 (s, 3 H, $CH_3COH$), 1.30–1.41 (m, 2 H, $CH_aH_bCH_2CH_2CC(O)$ + $CH_2CH_2CH_aH_bCC(O)$), 1.44–1.59 (m, 2 H, $CH_2CH_aH_bCH_2CC(O)$ + $CH_aH_bCH_2CH_2CC(O)$), 1.64–1.83 (m, 3 H, $CH_2CH_aH_bCH_2CC(O)$ + $CH_2CHCH_2Ph$), 2.42 (td, $J$ = 14.0, 4.1 Hz, 1 H, $CH_2CH_2CH_aH_bCC(O)$), 2.61 (dd, $J$ = 13.0, 6.2 Hz, 1 H, $CH_2CHCH_aH_bPh$), 2.87 (dd, $J$ = 13.0, 8.6 Hz, 1 H, $CH_2CHCH_aH_bPh$), 3.57–3.67 (m, 1 H, $CH_2CHCH_2Ph$), 7.10–7.20 (m, 3 H, Ar$H$), 7.24 (t, $J$ = 7.2 Hz, 2 H, Ar$H$) ppm; $^{13}$C-NMR (100 MHz, $CDCl_3$) δ 20.7 ($(CH_3)_a(CH_3)_bC$), 21.0 ($CH_2CH_2CH_2CC(O)$), 25.6 ($(CH_3)_a(CH_3)_bC$), 34.6 ($CH_2CH_2CH_2CC(O)$), 35.7 ($CH_3COH$), 37.3 ($CH_2CH_2CH_2CC(O)$), 40.7 ($CH_2CHCH_2Ph$), 43.5 ($CH_2CHCH_2Ph$), 46.9 ($(CH_3)_2C$), 51.2 ($CH_2CHCH_2Ph$), 71.6 ($CH_3COH$), 126.2 (Ar$C$H), 128.2 (Ar$C$H), 129.5 (Ar$C$H), 139.8 (Ar$C$), 222.8 ($C$(O)) ppm; IR $\nu_{max}$ (thin film/cm$^{-1}$): 3494 (O-H), 2966, 2924, 2866, 1686 (C=O), 1455, 1365, 1104, 1048; HRMS calcd for $C_{18}H_{26}O_2Na$ $[M+Na]^+$: 297.1825, found 297.1822.

***rac*-(1*S*,6*R*,8*S*)-6-Benzyl-8-methyl-9-oxabicyclo[4.2.1]nonan-1-ol (3k)**

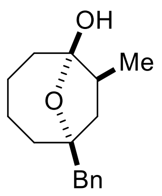

Prepared according to general procedure F using lactone **1k** (100 mg, 0.43 mmol), 0.1 M $SmI_2$ in THF (32 mL, 3.2 mmol) and $H_2O$ (5.9 mL, 320 mmol). Oxidation of the crude mixture was carried out using Dess-Martin periodinane (270 mg, 0.65 mmol) in dry $CH_2Cl_2$ (5.4 mL). The crude mixture was then purified by silica gel column chromatography (hexane/EtOAc, 90:10 to 70:30). The title product was obtained as a 61:39 mixture of diastereomers (30 mg, 0.120 mmol, 28%). $^1$H-NMR (400 MHz, $CDCl_3$) δ 0.74 (d, $J$ = 6.8 Hz, 3 H, C$H_3$ minor diastereomer), 0.91 (d, $J$ = 6.8 Hz, 3 H, C$H_3$ major diastereomer), 1.04–2.23 (m, 11 H for each diastereomer, C$H_2$CH$_2$CH$_2$CH$_2$COH + CH$_2$C$H_2$CH$_2$CH$_2$COH + CH$_2$CH$_2$C$H_2$CH$_2$COH + CH$_2$CH$_2$CH$_2$C$H_2$COH + C$H_2$CHCH$_3$ + CH$_2$C$H$CH$_3$ for both diastereomers), 2.66 (d, $J$ = 13.6 Hz, 1 H, C$H_a$H$_b$Ar minor diastereomer), 2.79 (d, $J$ = 13.6 Hz, 1 H, C$H_a$H$_b$Ar major diastereomer), 2.87–2.96 (m, 1 H for each diastereomer, CH$_a$$H_b$Ar for both diastereomers), 7.11–7.36 (m, 5 H for each diastereomer, ArC$H$ for both diastereomers) ppm; $^{13}$C-NMR (100 MHz, $CDCl_3$) δ 13.5 (CH$_2$CH$C$H$_3$, major diastereomer), 15.5 (CH$_2$CH$C$H$_3$, minor diastereomer), 22.2 (CH$_2$CH$_2$$C$H$_2$CH$_2$COH, major diastereomer), 23.6 (CH$_2$CH$_2$$C$H$_2$CH$_2$COH, minor diastereomer), 24.0 (CH$_2$$C$H$_2$CH$_2$CH$_2$COH, minor diastereomer), 24.9 (CH$_2$$C$H$_2$CH$_2$CH$_2$COH, major diastereomer), 36.5 (CH$_2$CH$_2$CH$_2$$C$H$_2$COH, major diastereomer), 39.5 (CH$_2$$C$HCH$_3$, minor diastereomer), 40.8 (CH$_2$CH$_2$CH$_2$$C$H$_2$COH, minor diastereomer), 41.1 ($C$H$_2$CH$_2$CH$_2$CH$_2$COH, minor diastereomer), 41.5 ($C$H$_2$CHCH$_3$, major diastereomer), 41.5 ($C$H$_2$CH$_2$CH$_2$CH$_2$COH, major diastereomer), 44.3 ($C$H$_2$CHCH$_3$, minor diastereomer), 44.7 (CH$_2$$C$HCH$_3$, major diastereomer), 48.0 (Ar$C$H$_2$, minor diastereomer), 48.0 (Ar$C$H$_2$, major diastereomer), 80.6 (ArCH$_2$$C$O, major diastereomer), 80.9 (ArCH$_2$$C$O, minor diastereomer), 107.5 (O$C$OH, minor diastereomer), 108.3 (O$C$OH, major diastereomer), 125.7 (Ar$C$H, minor diastereomer), 126.2 (Ar$C$H, major diastereomer), 127.7 (Ar$C$H, major diastereomer), 127.8 (Ar$C$H, minor diastereomer), 130.7 (Ar$C$H, minor diastereomer), 130.8 (Ar$C$H, major diastereomer), 137.8

(Ar*C*, major diastereomer), 138.0 (Ar*C*, minor diastereomer) ppm; IR $\nu_{max}$ (thin film/cm$^{-1}$): 3413 (O–H), 3026, 2929, 1710, 1495, 1453, 1086; HRMS calcd for $C_{16}H_{22}O_2Na$ [M+Na]$^+$: 269.1517, found 269.1510.

**rac-(1*S*,6*R*,8*S*)-6,8-Dibenzyl-9-oxabicyclo[4.2.1]nonan-1-ol (3l)**

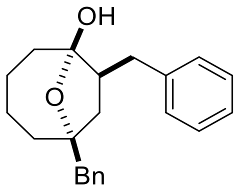

Prepared according to general procedure F using lactone **1l** (65 mg, 0.20 mmol), 0.1 M $SmI_2$ in THF (16 mL, 1.60 mmol) and $H_2O$ (2.9 mL, 160 mmol). Oxidation of the crude mixture was carried out using Dess-Martin periodinane (0.13 g, 0.30 mmol) in dry $CH_2Cl_2$ (2.5 mL). The crude mixture was then purified by silica gel column chromatography (hexane/EtOAc, 90:10 to 70:30). The title product was obtained as a 80:20 mixture of diastereomers (43 mg, 0.132 mmol, 66%). Spectroscopic data of major diastereomer: $^1$H-NMR (400 MHz, $CDCl_3$) δ 1.32–1.44 (m, 1 H, $CH_2C\mathit{H}CH_2Ar$), 1.46–2.02 (m, 9 H, $C\mathit{H}_2CH_2CH_2CH_2COH$ + $CH_2C\mathit{H}_2CH_2CH_2COH$ + $CH_2CH_2C\mathit{H}_2CH_2COH$ + $CH_2CH_2CH_2C\mathit{H}_aH_bCOH$ + $C\mathit{H}_2CHCH_2Ar$), 2.04–2.19 (m, 1 H, $CH_2CH_2CH_2CH_a\mathit{H}_bCOH$), 2.35–2.48 (m, 1 H, $CH_2CHC\mathit{H}_aH_bAr$), 2.62 (d, *J* = 13.2 Hz, 1 H, $CC\mathit{H}_aH_bAr$), 2.79–2.91 (m, 2 H, $CH_2CHCH_a\mathit{H}_bAr$ + $CCH_a\mathit{H}_bAr$), 6.96 (d, *J* = 8.0 Hz, 2 H, Ar*H*), 7.09–7.36 (m, 8 H, Ar*H*) ppm; $^{13}$C-NMR (100 MHz, $CDCl_3$) δ 22.4 ($CH_2CH_2\mathit{C}H_2CH_2COH$), 24.9 ($CH_2\mathit{C}H_2CH_2CH_2COH$), 35.2 ($CH_2CH\mathit{C}H_2Ar$), 37.1 ($CH_2CH_2CH_2\mathit{C}H_2COH$), 39.7 ($\mathit{C}H_2CHCH_2Ar$), 40.8 ($\mathit{C}H_2CH_2CH_2CH_2COH$), 48.0 ($C\mathit{C}H_2Ar$), 51.4 ($CH_2\mathit{C}HCH_2Ar$), 80.6 ($ArCH_2\mathit{C}O$), 107.6 (O*C*OH), 125.9 (Ar*C*H), 126.3 (Ar*C*H), 127.7 (Ar*C*H), 128.2 (Ar*C*H), 128.3 (Ar*C*H), 130.8 (Ar*C*H), 137.6 (Ar*C*), 140.2 (Ar*C*),  ppm; IR $\nu_{max}$ (thin film/cm$^{-1}$): 3400

(O−H), 3025, 2929, 2857, 1708, 1495, 1452, 1081; HRMS cald for $C_{22}H_{26}O_2Na$ $[M+Na]^+$: 345.1838, found 345.1831.

***rac*-(1*S*,6*R*,8*S*)-6-Benzyl-8-(2-fluorobenzyl)-9-oxabicyclo[4.2.1]nonan-1-ol (3m)**

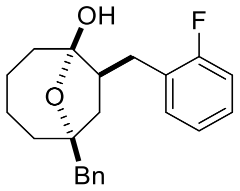


Prepared according to general procedure F using lactone **1m** (68 mg, 0.20 mmol), 0.1 M $SmI_2$ in THF (16 mL, 1.60 mmol) and $H_2O$ (2.9 mL, 160 mmol). Oxidation of the crude mixture was carried out using Dess-Martin periodinane (0.13 g, 0.30 mmol) in dry $CH_2Cl_2$ (2.5 mL). The crude mixture was then purified by silica gel column chromatography (hexane/EtOAc, 90:10 to 70:30). The title product was obtained as a 83:17 mixture of diastereomers (62 mg, 0.182 mmol, 91%). Spectroscopic data of major diastereomer: $^1$H-NMR (400 MHz, $CDCl_3$) δ 1.31–1.44 (m, 1 H, $CH_2C\textit{H}CH_2Ar$), 1.50–2.00 (m, 9 H, $C\textit{H}_2CH_2CH_2CH_2COH$ + $CH_2C\textit{H}_2CH_2CH_2COH$ + $CH_2CH_2C\textit{H}_2CH_2COH$ + $CH_2CH_2CH_2C\textit{H}_aH_bCOH$ + $C\textit{H}_2CHCH_2Ar$), 2.06–2.16 (m, 1 H, $CH_2CH_2CH_2CH_a\textit{H}_bCOH$), 2.56 (dd, *J* = 13.8, 11.0 Hz, 1 H, $CH_2CHC\textit{H}_aH_bAr$), 2.63 (d, *J* = 13.4 Hz, 1 H, $CC\textit{H}_aH_bAr$), 2.75 (dd, *J* = 13.8, 4.2 Hz, 1 H, $CH_2CHCH_a\textit{H}_bAr$), 2.86 (d, *J* = 13.4 Hz, 1 H, $CCH_a\textit{H}_bAr$), 6.90 (td, *J* = 7.4, 1.2 Hz, 1 H, Ar*H*), 6.94–7.08 (m, 2 H, Ar*H*), 7.09–7.35 (m, 6 H, Ar*H*) ppm; $^{13}$C-NMR (100 MHz, $CDCl_3$) δ 22.4 ($CH_2CH_2\textit{C}H_2CH_2COH$), 24.9 ($CH_2\textit{C}H_2CH_2CH_2COH$), 28.0 ($CH_2CH\textit{C}H_2Ar$), 37.0 ($CH_2CH_2CH_2\textit{C}H_2COH$), 39.3 ($\textit{C}H_2CHCH_2Ar$), 40.7 ($\textit{C}H_2CH_2CH_2CH_2COH$), 48.0 ($C\textit{C}H_2Ar$), 50.5 ($CH_2\textit{C}HCH_2Ar$), 80.6 ($ArCH_2\textit{C}O$), 107.5 (O*C*OH), 115.1 (*J* = 22.6 Hz, Ar*C*H), 123.9 (*J* = 3.7 Hz, Ar*C*H), 126.3 (Ar*C*H), 127.1 (*J* = 15.3 Hz, Ar*C*), 127.6 (*J* = 8.0 Hz, Ar*C*H), 127.7 (Ar*C*H), 130.1 (*J* = 4.4 Hz, Ar*C*H), 130.7 (Ar*C*H), 137.6 (Ar*C*), 160.9 ( *J* = 242.8 Hz, Ar*C*F), ppm; $^{19}$F-NMR (376 MHz, $CDCl_3$) δ −118.4 (m, Ar*F*) ppm; IR $\nu_{max}$ (thin

film/cm$^{-1}$): 3399 (O−H), 3027, 2929, 2858, 1708, 1584, 1491, 1453, 1228; HRMS cald for $C_{22}H_{25}FO_2Na$ [M+Na]$^+$: 363.1737, found 363.1736.

**General Procedure G: $SmI_2$-$H_2O$-Mediated Radical Cyclization**

***rac*-(2*S*,4*R*)-2-Benzylcyclooctane-1,4-diol (2n)**

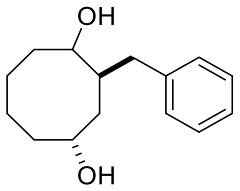

Prepared according to general procedure G using lactone **1n** (69 mg, 0.30 mmol), 0.1 M $SmI_2$ in THF (24 mL, 2.40 mmol) and $H_2O$ (4.3 mL, 240 mmol). The crude mixture was purified by preparative TLC (EtOAc). The title product was obtained as a 50:50 mixture of diastereomers (39 mg, 0.171 mmol, 57%). $^1$H-NMR (400 MHz, $CDCl_3$) δ 1.36–1.94 (m, 19 H, C$H_2$ × 8 + C$H_a$H$_b$ × 2 + HOCHCH$_2$C*H* for diastereomer 1), 1.99–2.12 (m, 3 H, CH$_a$$H_b$ × 2 + HOCHCH$_2$C*H* for diastereomer 2), 2.25–2.36 (m, 2 H, C$H_a$H$_b$Ph for both diastereomers), 3.17–3.29 (m, 2 H, CH$_a$$H_b$Ph for both diastereomers), 3.35–3.45 (m, 1 H, HOC*H*CH$_2$CH for diastereomer 1), 3.57–3.69 (m, 2 H, CH$_2$CH$_2$CH$_2$CH$_2$C*H*OH for both diastereomers), 3.83–3.91 (m, 1 H, HOC*H*CH$_2$CH for diastereomer 2), 7.17–7.35 (m, 10 H, Ar*H* for both diastereomers) ppm; $^{13}$C-NMR (100 MHz, $CDCl_3$) δ 21.2 (*C*H$_2$), 21.5 (*C*H$_2$), 21.6 (*C*H$_2$), 22.8 (*C*H$_2$), 31.5 (*C*H$_2$), 32.8 (*C*H$_2$), 33.7 (*C*H$_2$), 33.8 (*C*H$_2$), 35.1 (HOCH*C*H$_2$CH, diastereomer 2), 37.2 (*C*H$_2$), 40.4 (*C*H$_2$Ph, diastereomer 2), 41.2 (HOCHCH$_2$*C*H, diastereomer 2), 41.7 (*C*H$_2$Ph, diastereomer 1), 42.5 (HOCHCH$_2$*C*H, diastereomer 1), 68.7 (HO*C*HCH$_2$CH, diastereomer 2), 72.8 (HO*C*HCH$_2$CH, diastereomer 1), 75.5 (CH$_2$CH$_2$CH$_2$CH$_2$*C*HOH, diastereomer 2), 75.8 (CH$_2$CH$_2$CH$_2$CH$_2$*C*HOH, diastereomer 1), 126.1 (Ar*C*H), 126.2 (Ar*C*H), 128.4 (Ar*C*H), 128.5 (Ar*C*H), 129.3 (Ar*C*H), 129.4 (Ar*C*H),

140.6 (Ar*C*), 140.8 (Ar*C*) ppm; IR $\nu_{max}$ (thin film/cm$^{-1}$): 3350 (O–H), 2926, 2858, 1707, 1452, 1022; HRMS cald for $C_{15}H_{22}O_2Na$ $[M+Na]^+$: 257.1527, found 257.1517.

***rac*-(2*S*,4*R*)-2-(2-Fluorobenzyl)cyclooctane-1,4-diol (2o)**

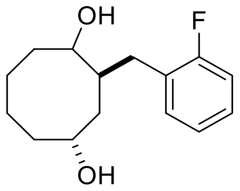


Prepared according to general procedure G using lactone **1o** (75 mg, 0.30 mmol), 0.1 M $SmI_2$ in THF (24 mL, 2.40 mmol) and $H_2O$ (4.3 mL, 240 mmol). The crude mixture was purified by preparative TLC (EtOAc). The title product was obtained as a 62:21:17 mixture of diastereomers (27 mg, 0.126 mmol, 42%). The major diastereomer could be isolated and was obtained as a white powder. $^1$H-NMR (400 MHz, $CDCl_3$) δ 1.29–1.54 (m, 3 H, C$H_a$H$_b$ × 2 + HOCHC$H_a$H$_b$CH), 1.62–1.86 (m, 6 H, CH$_a$$H_b$ × 2 + C$H_2$ × 2), 2.14–2.29 (m, 2 H, HOCHCH$_2$C*H* + HOCHCH$_a$$H_b$CH), 2.67 (dd, *J* = 13.3, 7.4 Hz, C$H_a$H$_b$Ar), 2.85 (dd, *J* = 13.3, 7.2 Hz, CH$_a$$H_b$Ar), 3.70–3.77 (m, 1 H, CH$_2$CH$_2$CH$_2$CH$_2$C*H*OH), 3.92–4.02 (m, 1 H, HOC*H*CH$_2$CH), 7.00–7.12 (m, 2 H, Ar*H*), 7.16–7.26 (m, 2 H, Ar*H*) ppm; $^{13}$C-NMR (100 MHz, $CDCl_3$) δ 20.2 (*C*H$_2$), 23.2 (*C*H$_2$), 32.8 (*C*H$_2$), 32.9 (*C*H$_2$), 34.4 (HOCH*C*H$_2$CH), 34.6 (*C*H$_2$Ar), 36.8 (HOCHCH$_2$*C*H), 69.7 (HO*C*HCH$_2$CH), 71.4 (CH$_2$CH$_2$CH$_2$CH$_2$*C*HOH), 115.4 (d, *J* = 22.4 Hz, Ar*C*H), 124.9 (d, *J* = 3.0 Hz, Ar*C*H), 127.7 (d, *J* = 15.5 Hz, Ar*C*), 127.9 (d, *J* = 7.8 Hz, Ar*C*H), 131.7 (d, *J* = 4.9 Hz, Ar*C*H), 161.5 (d, *J* = 243.0 Hz, Ar*C*) ppm; $^{19}$F-NMR (376 MHz, $CDCl_3$) δ −118.0 (m, Ar*F*) ppm; IR $\nu_{max}$ (thin film/cm$^{-1}$): 3351 (O–H), 2928, 1491, 1453, 1227, 1026; HRMS cald for $C_{15}H_{21}FO_2Na$ $[M+Na]^+$: 275.1432, found 275.1423.

***rac*-(1*S*,6*R*,8*S*)-6-Methyl-8-((*S*)-phenylmethyl-d)-9-oxabicyclo[4.2.1]nonan-1-ol (*d*-3a)**

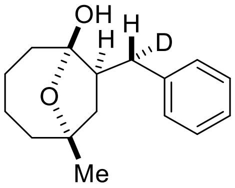


Prepared according to general procedure F using lactone **1a** (50 mg, 0.205 mmol), 0.1 M $SmI_2$ in THF (16.4 mL, 1.64 mmol) and $D_2O$ (3.0 mL, 164 mmol). Oxidation of the crude mixture was carried out using Dess-Martin periodinane (131 mg, 0.308 mmol) in dry $CH_2Cl_2$ (2.6 mL). The crude mixture was then purified by silica gel column chromatography (hexane/EtOAc, 90:10). The title product was obtained as a 75:25 mixture of diastereomers (28 mg, 0.113 mmol, 55%). The major cyclization diastereomer could be isolated as a 90:10 mixture of deuteration diastereomers; it was obtained as colorless crystals: mp ($CH_2Cl_2$) 88–91 °C. $^1$H-NMR (400 MHz, $CDCl_3$) δ 1.27 (s, 3 H, $CH_3$), 1.50–1.93 (m, 9 H, $CH_2CH_2CH_2CH_2COH$ + $CH_2CH_2CH_2CH_2COH$ + $CH_2CH_2CH_2CH_2COH$ + $CH_2CH_2CH_2CH_aH_bCOH$ + $CH_2CHCH_2Ar$), 2.12–2.34 (m, 2 H, $CH_2CH_2CH_2CH_aH_bCOH$ + $CHCH_2Ar$), 2.56 (d, *J* = 10.4 Hz, 1 H, $CH_aH_bAr$), 7.18–7.25 (m, 3 H, Ar*H*), 7.28–7.33 (m, 2 H, Ar*H*) ppm; $^{13}$C-NMR (100 MHz, $CDCl_3$) δ 22.4 ($CH_2CH_2CH_2CH_2COH$), 25.0 ($CH_2CH_2CH_2CH_2COH$), 31.1 ($CH_3$), 35.6 (t, *J* = 19.3 Hz, *C*HDAr), 37.8 ($CH_2CH_2CH_2CH_2COH$), 41.9 ($CH_2CH_2CH_2CH_2COH$), 42.9 ($CH_2CHCHDAr$), 51.8 (*C*HCHDAr), 78.7 ($CH_3$*C*O), 107.7 (O*C*OH), 126.0 (Ar*C*H), 128.4 (Ar-*C*H), 128.5 (Ar*C*H), 140.7 (Ar*C*) ppm; IR $\nu_{max}$ (thin film/cm$^{-1}$): 3386 (O-H), 3025, 2925, 2857, 1451, 1117, 1088, 946; HRMS calcd for $C_{16}H_{21}DO_2Na$ $[M+Na]^+$: 270.1575, found 270.1577.

# 4. X-ray structure of 3a

**CCDC: 1480731**

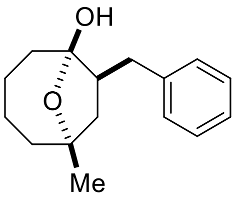


**3a**

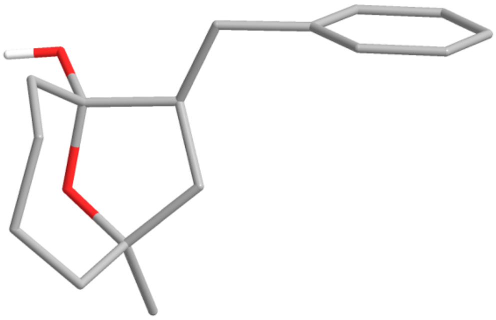

**Table S1. Crystal data and structure refinement for 3a**

| | |
|---|---|
| Empirical formula | $C_{16}H_{22}O_2$ |
| Formula weight | 246.34 |
| Temperature | 180(2) K |
| Wavelength | 1.54178 Å |
| Crystal system, space group | Monoclinic, $P2_1/c$ |
| Unit cell dimensions | a = 7.0377(3) Å alpha = 90º<br>b = 7.5716(3) Å beta = 94.249(2)º<br>c = 26.0367(9) Å gamma = 90º |
| Volume | 1383.60(9) $Å^3$ |
| Z, Calculated density | 4, 1.183 $Mg/m^3$ |
| Absorption coefficient | 0.596 $mm^{-1}$ |
| F(000) | 536 |
| Crystal size | 0.29 × 0.19 × 0.09 mm |
| Theta range for data collection | 6.09º to 72.43º |
| Limiting indices | -8<=h<=8, -8<=k<=9, -32<=l<=31 |
| Reflections collected / unique | 11660 / 2724 [R(int) = 0.0223] |
| Completeness to theta = 67.00º | 99.6% |
| Absorption correction | Semi-empirical from equivalents |
| Max. and min. transmission | 0.9483 and 0.814914 |

| | |
|---|---|
| Refinement method | Full-matrix least-squares on $F^2$ |
| Data / restraints / parameters | 2724 / 0 / 165 |
| Goodness-of-fit on $F^2$ | 1.037 |
| Final R indices [I>2sigma(I)] | R1 = 0.0359, wR2 = 0.0875 |
| R indices (all data) | R1 = 0.0384, wR2 = 0.0895 |
| Largest diff. peak and hole | 0.285 and -0.220 e.$A_{-3}$ |

## 5. X-ray structure of 3b

**CCDC: 1480730**

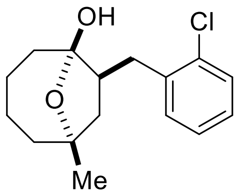


**3b**

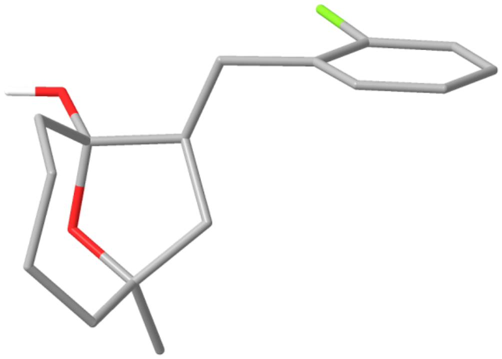

**Table S2. Crystal data and structure refinement for 3b**

| | |
|---|---|
| Empirical formula | $C_{16}H_{21}ClO_2$ |
| Formula weight | 280.78 |
| Temperature | 100(2) K |
| Wavelength | 1.5418 Å |
| Crystal system, space group | Triclinic, P -1 |
| Unit cell dimensions | a = 7.0537(4) Å alpha = 102.881(4)º<br>b = 7.6340(4) Å beta = 92.540(4)º<br>c = 14.9459(8) Å gamma = 113.534(5)º |
| Volume | 711.25(6) $Å^3$ |
| Z, Calculated density Mg/$m^3$ | 2, 1.311 |
| Absorption coefficient | 2.334 $mm^{-1}$ |
| F(000) | 300 |
| Crystal size | 0.28 × 0.17 × 0.05 mm |
| Theta range for data collection | 3.07º to 74.65º |
| Limiting indices | -8<=h<=8, -7<=k<=9, -18<=l<=15 |
| Reflections collected / unique | 4920 / 2688 [R(int) = 0.0179] |
| Completeness to theta = 67.00º | 96.0% |
| Absorption correction | Semi-empirical from equivalents |
| Max. and min. transmission | 1.00000 and 0.79811 |

| | |
|---|---|
| Refinement method | Full-matrix least-squares on $F^2$ |
| Data / restraints / parameters | 2688 / 0 / 174 |
| Goodness-of-fit on $F^2$ | 1.011 |
| Final R indices [I>2sigma(I)] | R1 = 0.0313, wR2 = 0.0852 |
| R indices (all data) | R1 = 0.0336, wR2 = 0.0869 |
| Largest diff. peak and hole | 0.327 and -0.246 e. Å$^{-3}$ |

## 6. X-ray structure of 3c

**CCDC: 1480729**

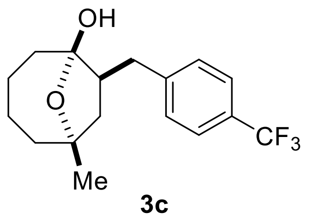


3c

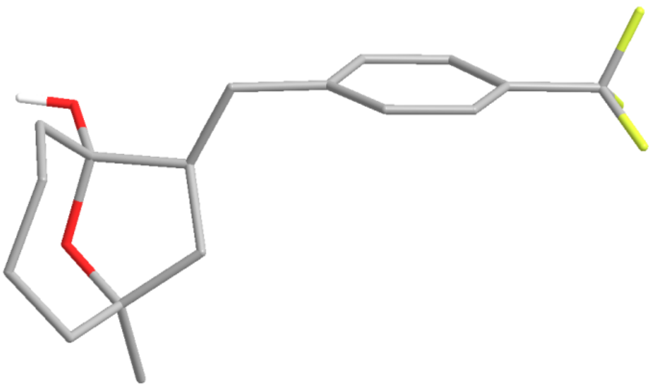

**Table S3. Crystal data and structure refinement for 3c**

| | |
|---|---|
| Empirical formula | $C_{17}H_{21}F_3O_2$ |
| Formula weight | 314.34 |
| Temperature | 100(2) K |
| Wavelength | 1.54178 Å |
| Crystal system, space group | Triclinic, P-1 |
| Unit cell dimensions | a = 6.8804(2) Å alpha = 79.6980(10)°<br>b = 7.4956(2) Å beta = 84.9710(10)°<br>c = 15.7412(4) Å gamma = 72.6250(10)° |
| Volume | 761.78(4) Å$^3$ |
| Z, Calculated density | 2, 1.370 Mg/m$^3$ |
| Absorption coefficient | 0.957 mm$^{-1}$ |
| F(000) | 332 |
| Crystal size | 0.30 × 0.28 × 0.19 mm |
| Theta range for data collection | 2.85° to 72.20° |
| Limiting indices | -8<=h<=8, -8<=k<=9, -19<=l<=19 |
| Reflections collected / unique | 7248 / 2879 [R(int) = 0.0161] |
| Completeness to theta = 67.00° | 96.3% |
| Absorption correction | Semi-empirical from equivalents |

| | |
|---|---|
| Max. and min. transmission | 0.8391 and 0.755146 |
| Refinement method | Full-matrix least-squares on $F^2$ |
| Data / restraints / parameters | 2879 / 0 / 201 |
| Goodness-of-fit on $F^2$ | 1.045 |
| Final R indices [I>2sigma(I)] | R1 = 0.0412, wR2 = 0.1055 |
| R indices (all data) | R1 = 0.0424, wR2 = 0.1064 |
| Largest diff. peak and hole | 0.338 and -0.279 e. Å $^{-3}$ |

# 7. X-ray structure of 3d

**CCDC: 1480728**

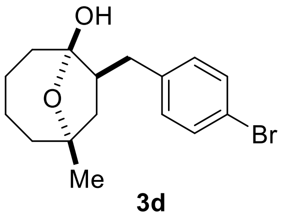


**3d**

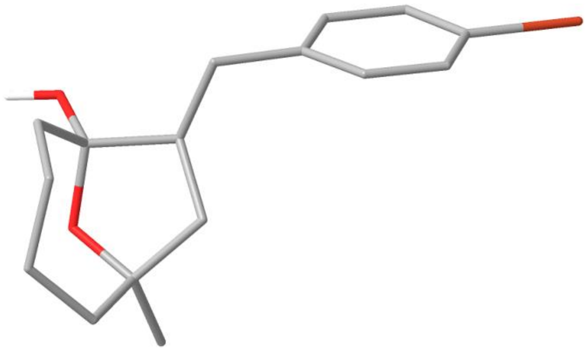

**Table S4. Crystal data and structure refinement for 3d**

| | |
|---|---|
| Empirical formula | $C_{16}H_{2}1BrO_{2}$ |
| Formula weight | 325.24 |
| Temperature | 100(2) K |
| Wavelength | 1.54178 Å |
| Crystal system, space group | Triclinic, P-1 |
| Unit cell dimensions | a = 6.8944(2) Å alpha = 77.0980(10)º |
| | b = 7.4598(2) Å beta = 87.1650(10)º |
| | c = 15.3176(4) Å gamma = 70.8460(10)º |
| Volume | 725.15(3) $Å^3$ |
| Z, Calculated density | 2, 1.490 $Mg/m^3$ |
| Absorption coefficient | 3.823 $mm^{-1}$ |
| F(000) | 336 |
| Crystal size | 0.25 × 0.23 × 0.08 mm |
| Theta range for data collection | 5.93º to 72.46º |
| Limiting indices | -8<=h<=8, -9<=k<=9, -18<=l<=18 |
| Reflections collected / unique | 7509 / 2747 [R(int) = 0.0162] |
| Completeness to theta = 67.00º | 96.3% |
| Absorption correction | Semi-empirical from equivalents |
| Max. and min. transmission | 0.7496 and 0.662763 |

| | |
|---|---|
| Refinement method | Full-matrix least-squares on $F^2$ |
| Data / restraints / parameters | 2747 / 0 / 174 |
| Goodness-of-fit on $F^2$ | 1.093 |
| Final R indices [I>2sigma(I)] | R1 = 0.0270, wR2 = 0.0680 |
| R indices (all data) | R1 = 0.0275, wR2 = 0.0685 |
| Largest diff. peak and hole | 0.675 and -0.542 e.Å$^{-3}$ |

## 8. Neutron diffraction studies on *d*-3a

A single crystal of the title compound was mounted on an Aluminium pin with adhesive tape and cooled to 100K on a closed cycle refrigerator. Data were collected at five fixed orientations using the single crystal time-of-flight Laue diffractometer SXD installed at the ISIS pulsed neutron source (Oxfordshire, UK).[6] Structure factors were obtained using the local SXD2001 software and corrected for absorption.[7]

**CCDC: 1480732**

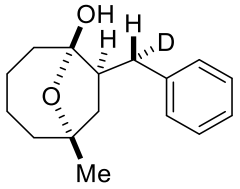


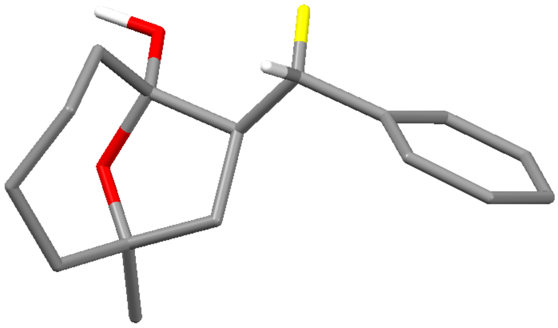

**Table S5. Experimental details for the TOF single-crystal neutron diffraction studies**

| | |
|---|---|
| Chemical formula | $C_{16}H_{21}D_1O_2$ |
| Formula weight | 247.36 |
| Temperature | 100(2) K |
| Wavelenght range λ | 0.23-8.80 Å |
| Crystal system, space group | Monoclinic, $P2_1/c$ |
| Unit cell dimensions | a = 6.994(2) Å<br>b = 7.523(2) Å beta = 94.18(2)º<br>c = 25.944(8) Å |
| Volume | 1361.4(7) $Å^3$ |

[6] Keen, D. A.; Gutmann, M. J.; Wilson, C. C. *J. Appl. Cryst.* **2006**, *39*, 714.
[7] Gutmann, M. J. (2005). SXD2001. ISIS Facility, Rutherford Appleton Laboratory, Oxfordshire, England.

| | |
|---|---|
| Z, Calculated density | 4, 1.2068 $Mg/m^3$ |
| Absorption coefficient | 5.3821 + 0.0115λ $cm^{-1}$ |
| F(000) | 184.3761 |
| Crystal size | 1 × 1 × 5 mm |
| Theta range for data collection | 8.40º to 82.29º |
| Limiting indices | -15<=h<=10, -16<=k<=20, -56<=l<=56 |
| Reflections collected / unique | 8923 / 4193 [R(int) = 0.1630] |
| Absorption correction | Numerical Gauss integration |
| Max. and min. transmission | 0.6578 and 0.2867 |
| Refinement method | Full-matrix least-squares on $F^2$ |
| Data / restraints / parameters | 8918 / 0 / 366 |
| Goodness-of-fit *S* | 2.43 (2.71) |
| Final R indices [I>2sigma(I)] | R1 = 0.0907, wR2 = 0.1702 |
| R indices (all data) | R1 = 0.1038, wR2 = 0.1738 |
| Largest diff. peak and hole | 4.96 and -3.43 fm.$Å^{-3}$ |

$R = \Sigma (|F_o| - |F_c|)/\Sigma (F_o)$    $R_w = \{\Sigma(w(F_o - F_c)^2) /\Sigma(w(F_o)^2)\}^{1/2}$

$R_w^2 = \{\Sigma(w(F_o^2 - F_c^2)^2) /\Sigma(w(F_o^2)^2)\}^{1/2}$    $R_\sigma = \Sigma [\sigma(F_o^2)] / \Sigma [F_o^2]$

$R_{int} = \Sigma \{n/(n-1\}^{1/2} | F_o^2 - F_o^2(mean) | / \Sigma F_o^2$ (summation is carried out only where more than one symmetry equivalent is averaged)

## 9. NMR spectra

400 MHz, $CDCl_3$

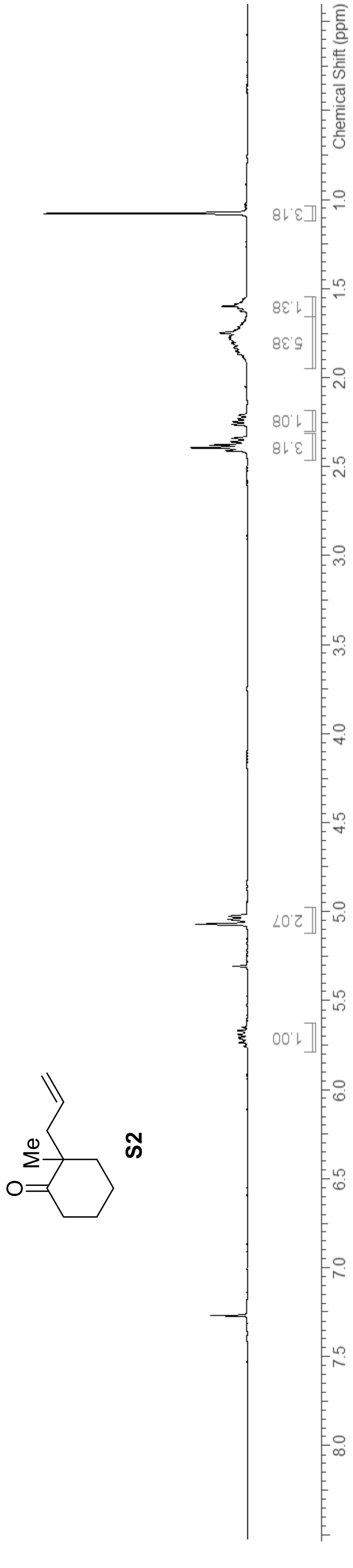


100 MHz, $CDCl_3$

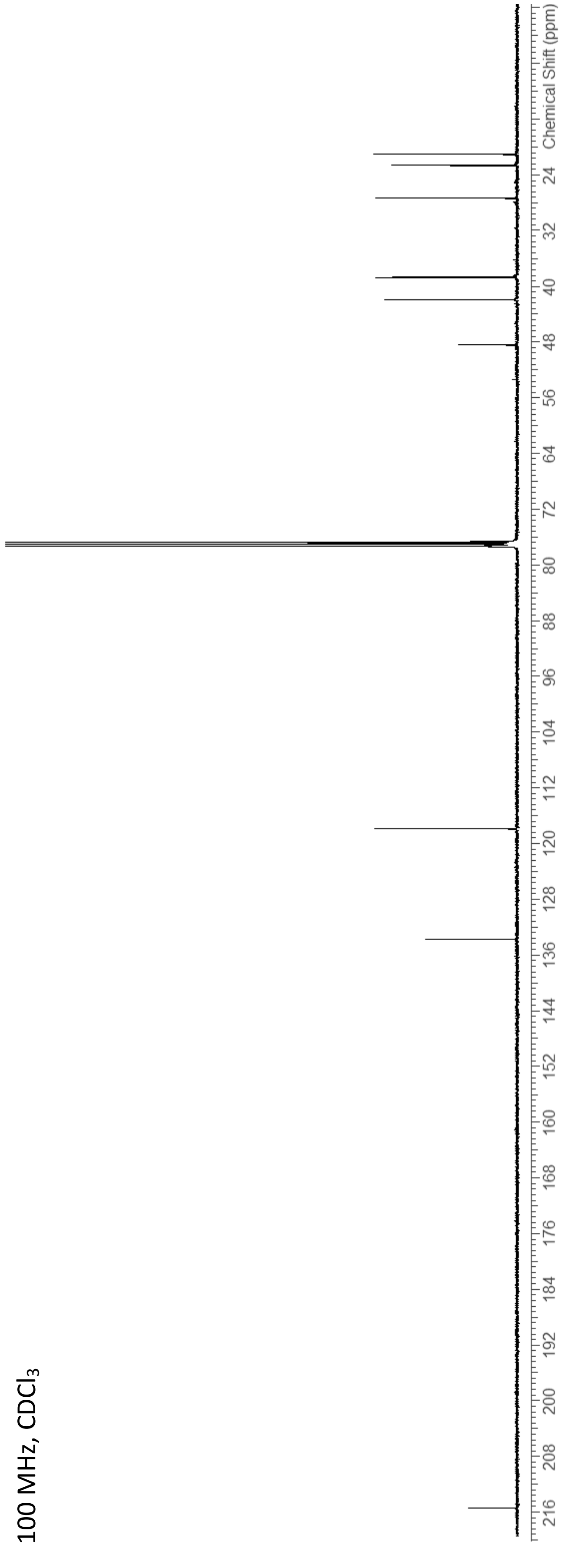

400 MHz, $CDCl_3$

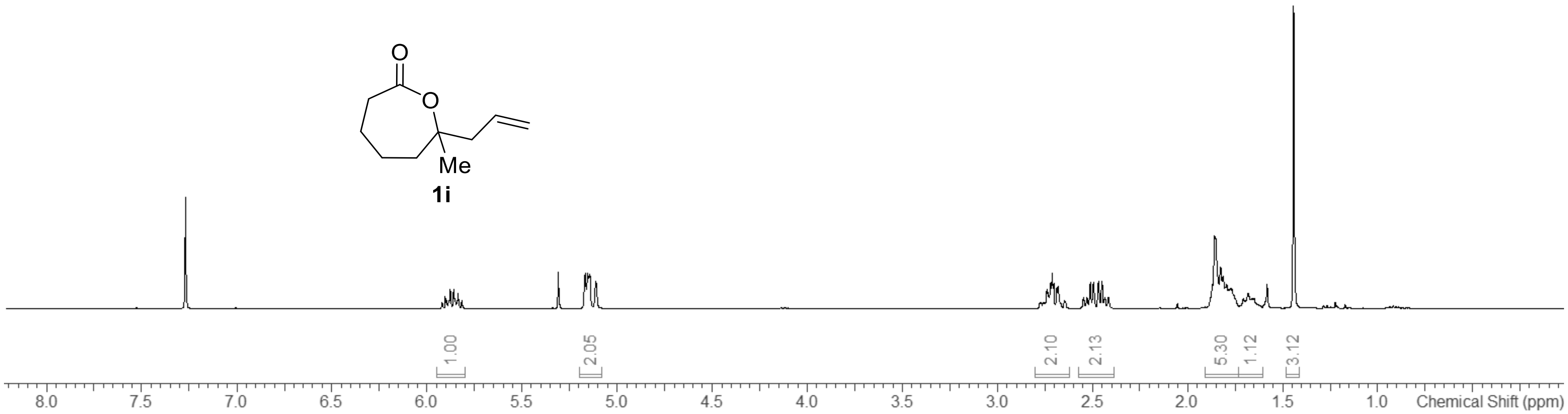


100 MHz, $CDCl_3$

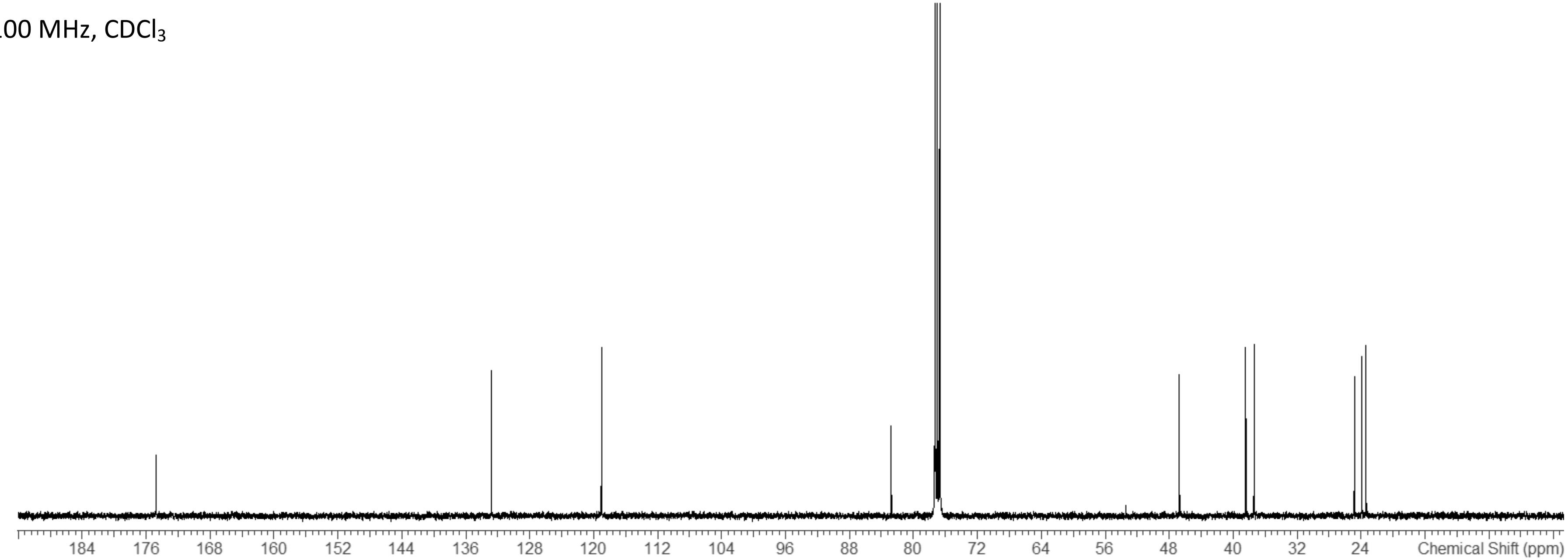

400 MHz, $CDCl_3$

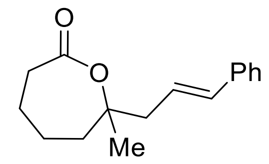
1a

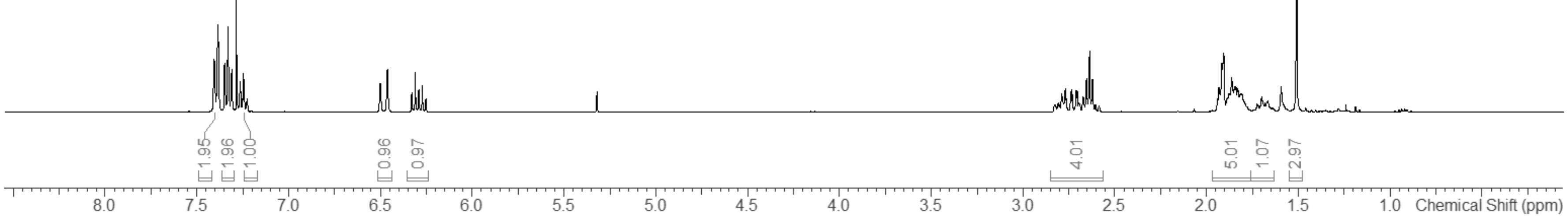


100 MHz, $CDCl_3$

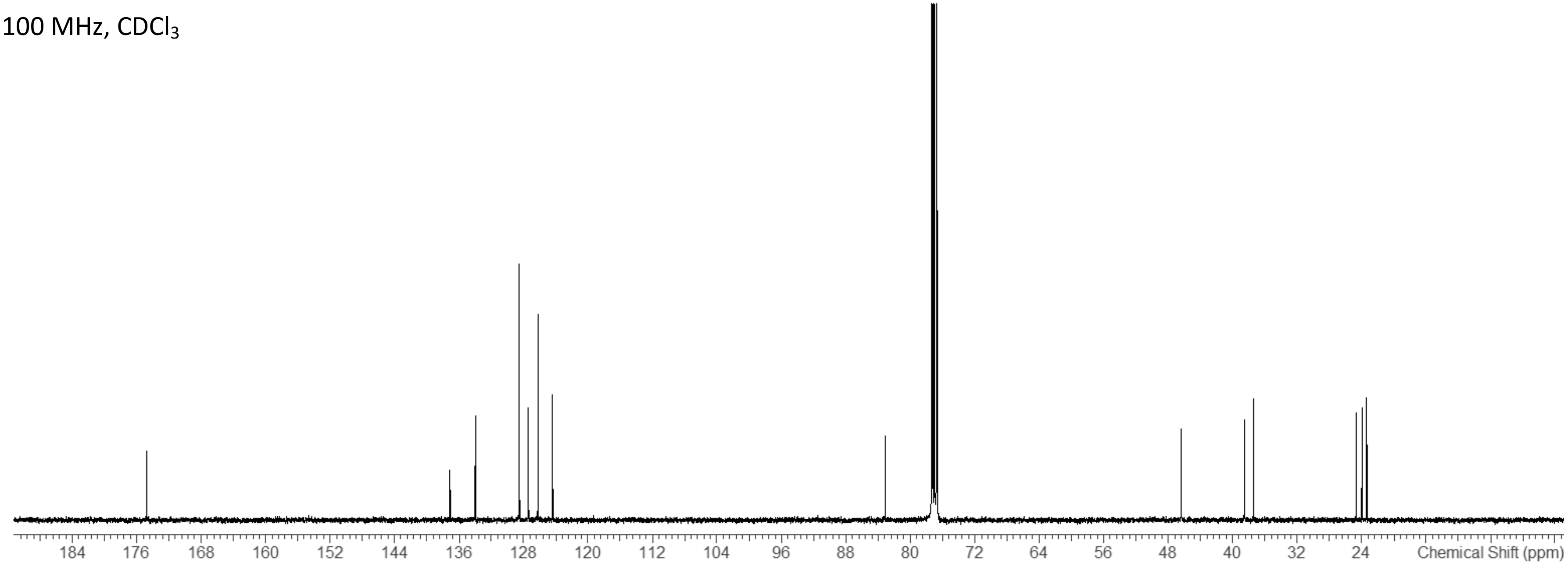

400 MHz, $CDCl_3$

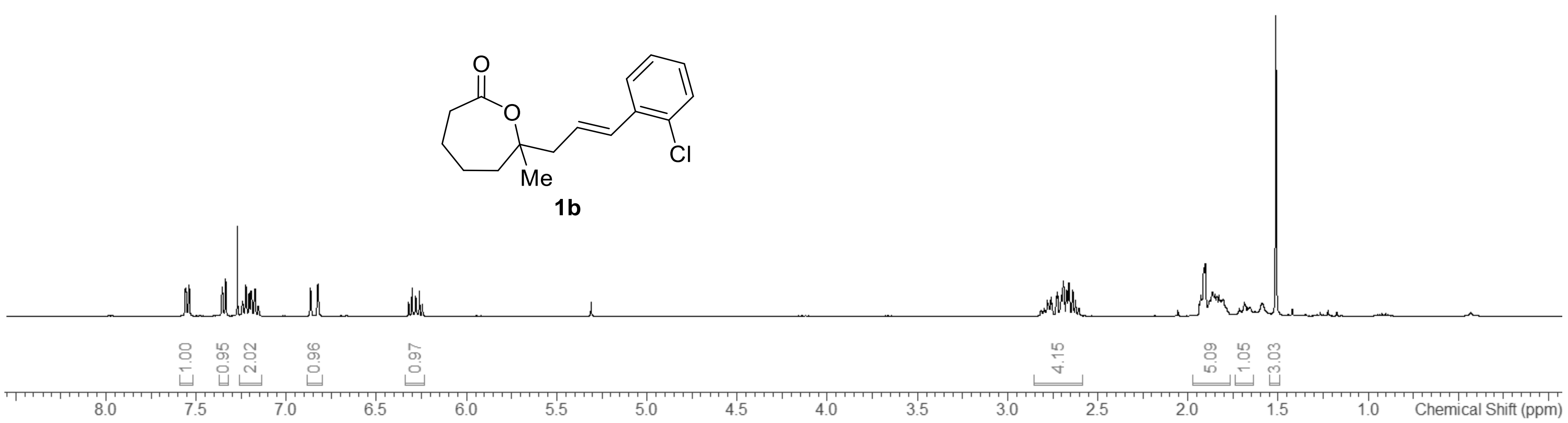


100 MHz, $CDCl_3$

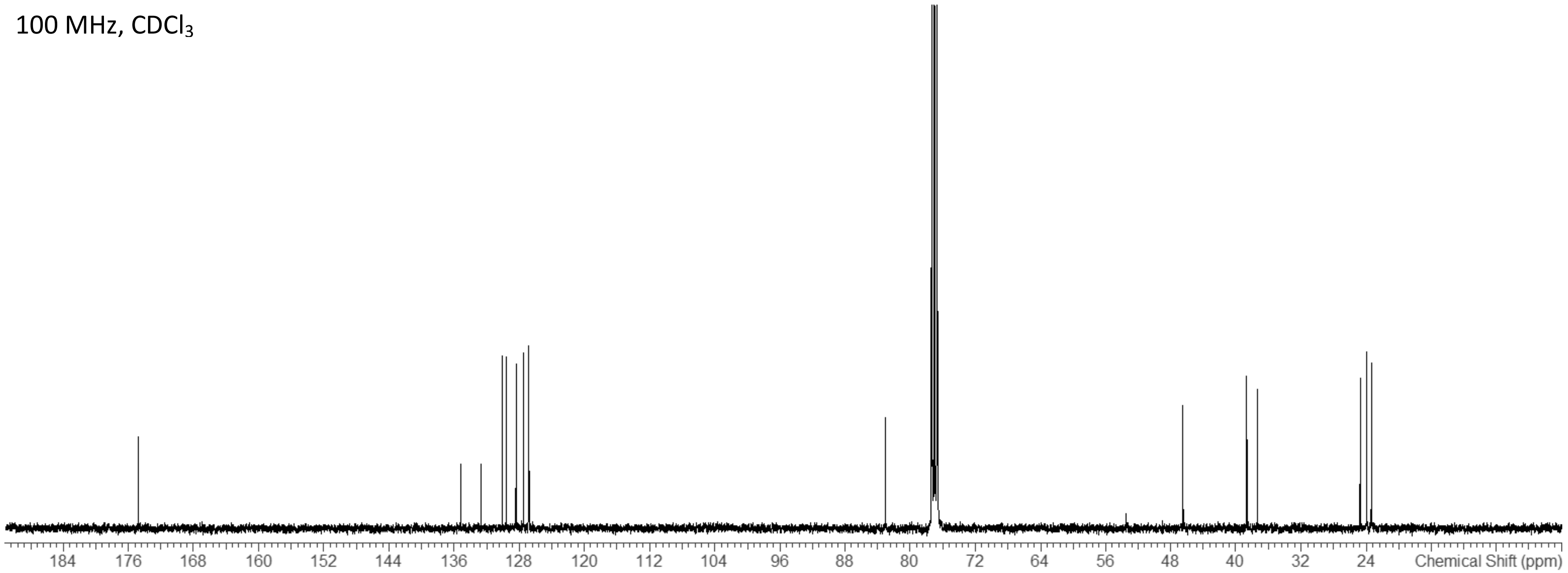

400 MHz, $CDCl_3$

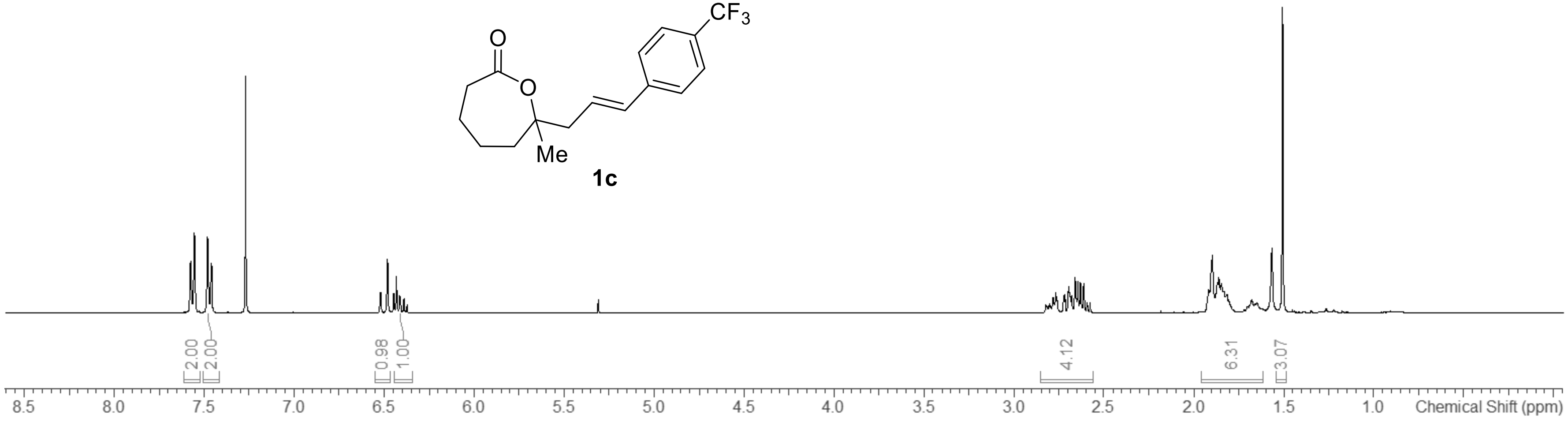


100 MHz, $CDCl_3$

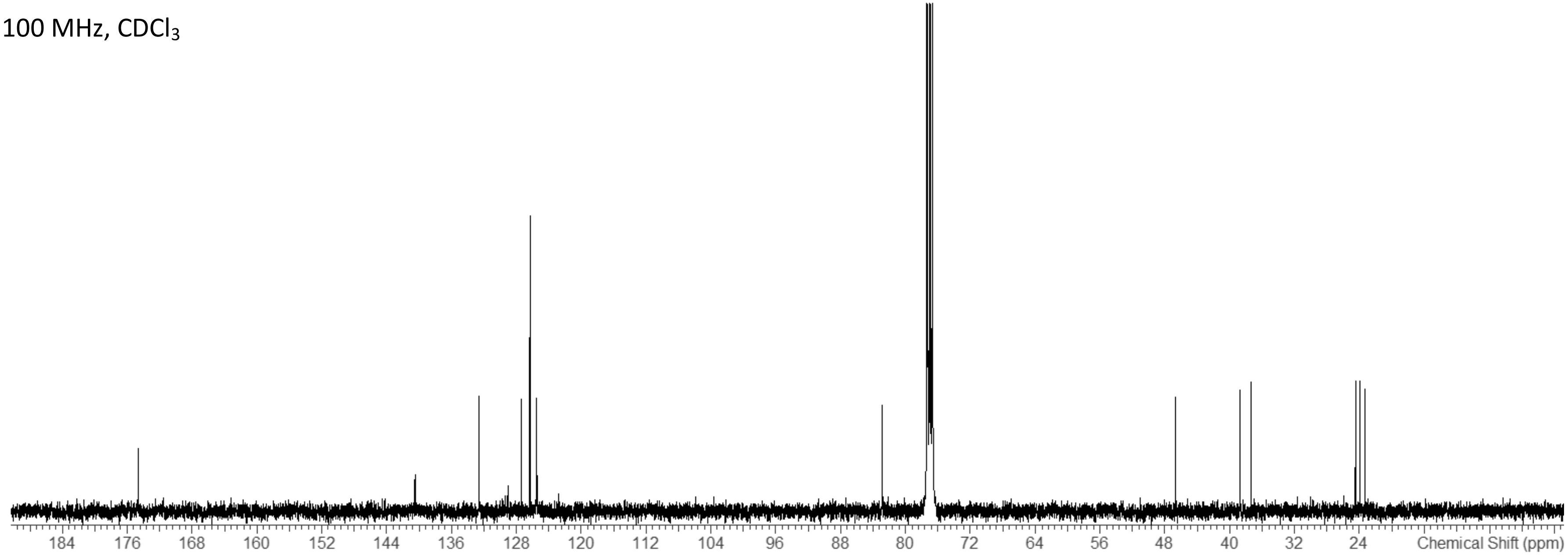

400 MHz, $CDCl_3$

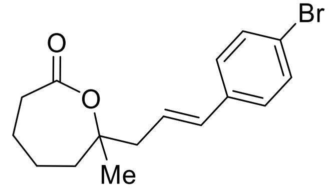

**1d**

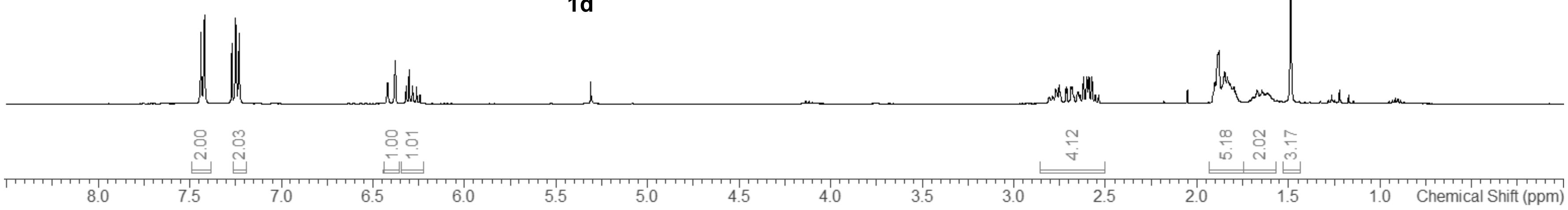


100 MHz, $CDCl_3$

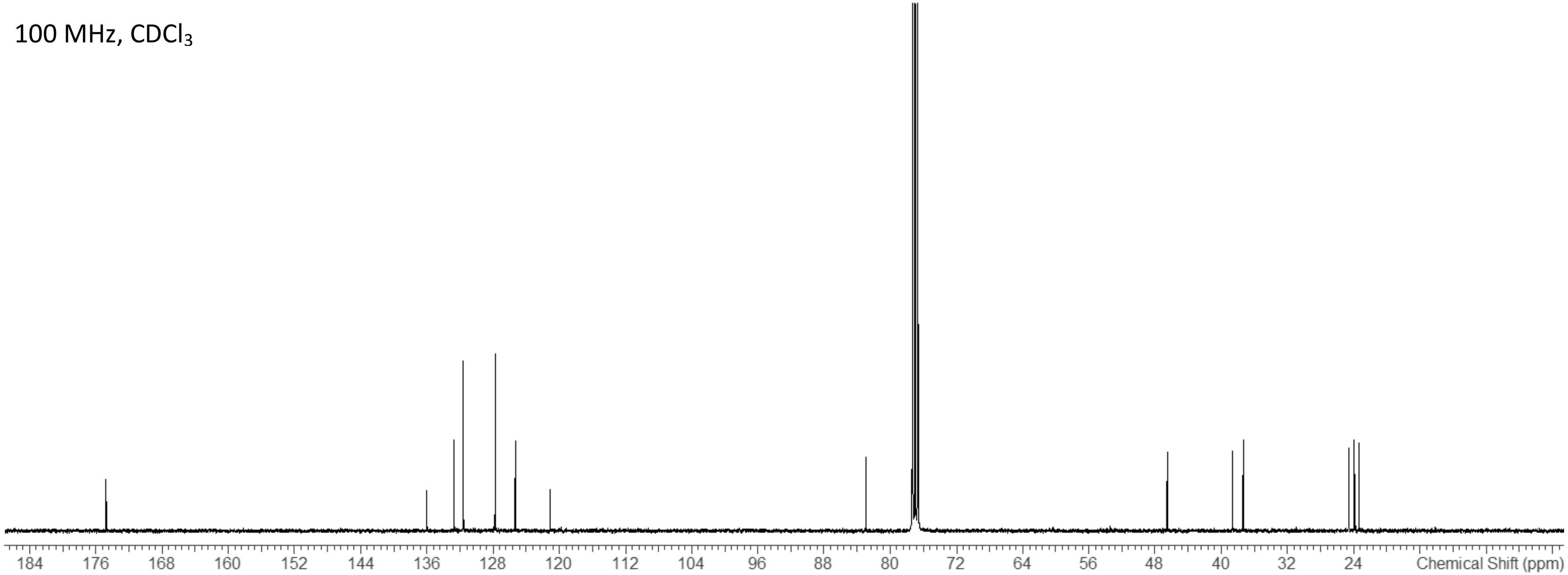

400 MHz, $CDCl_3$

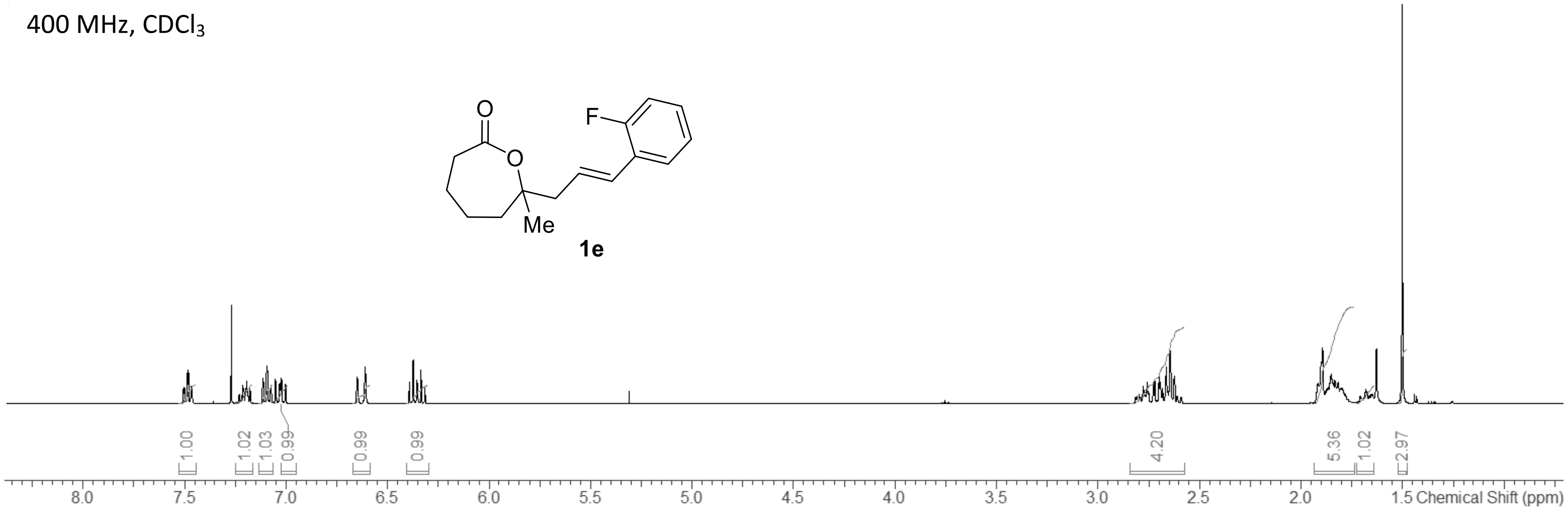


100 MHz, $CDCl_3$

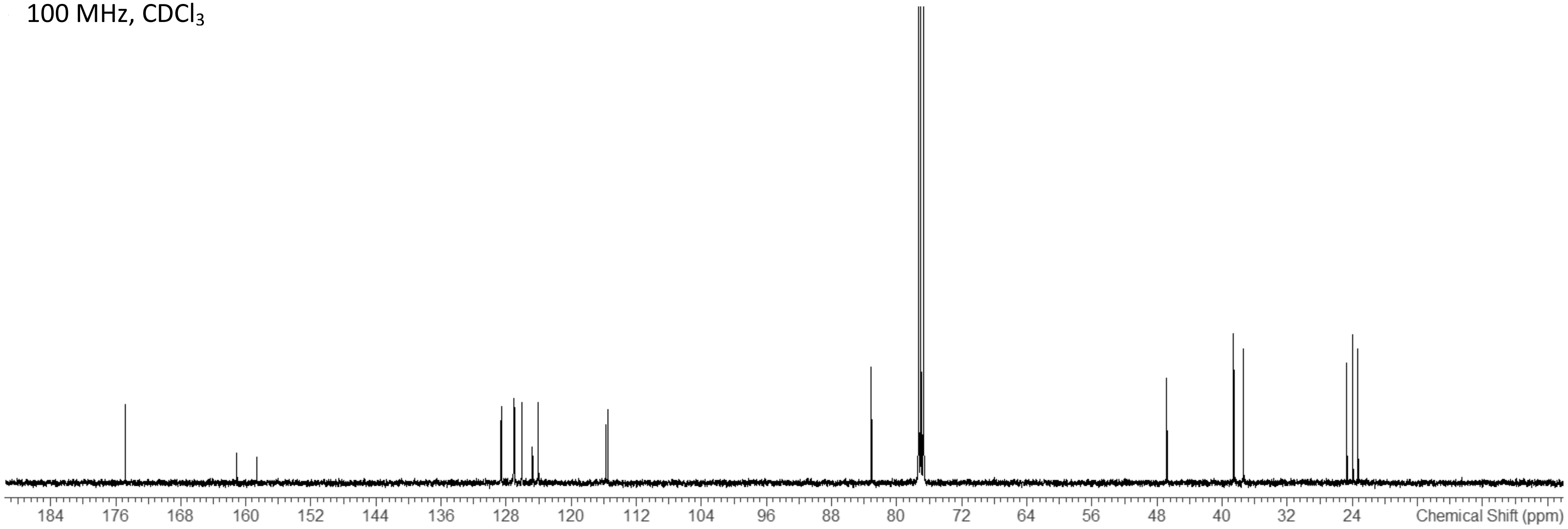

400 MHz, $CDCl_3$

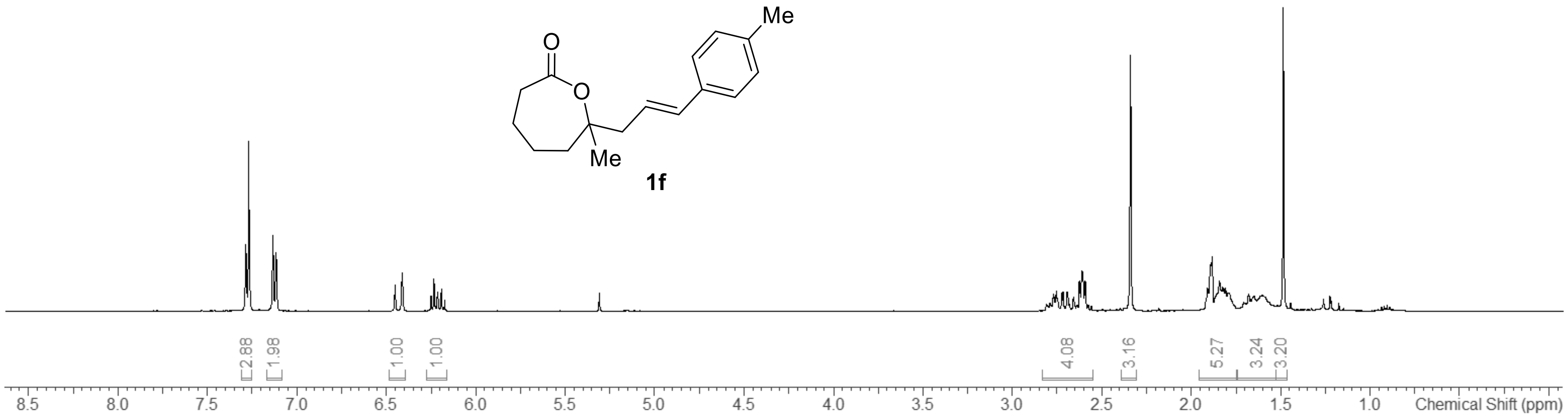


100 MHz, $CDCl_3$

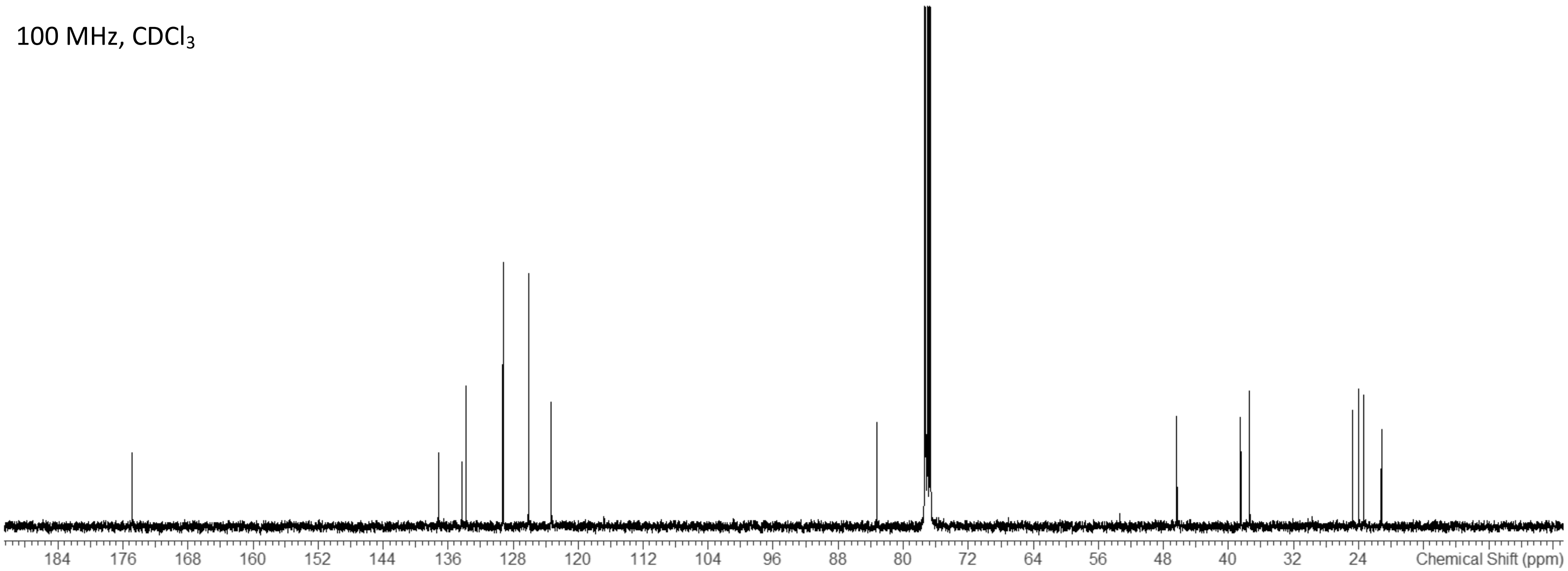

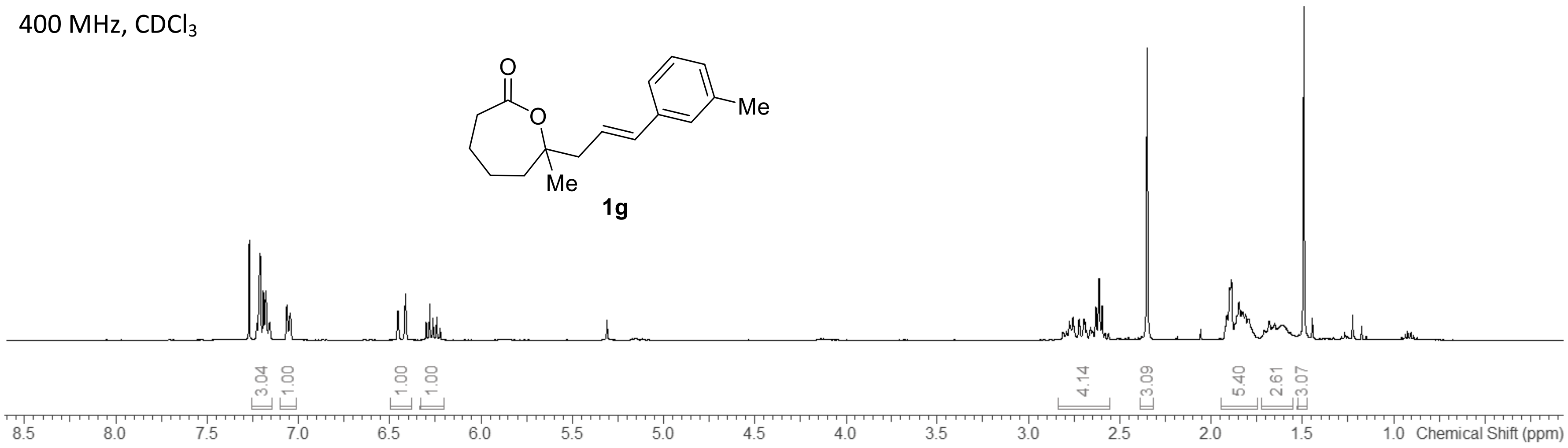
400 MHz, CDCl3
O
O
Me
Me
1g
3.04
1.00
1.00
1.00
4.14
3.09
5.40
2.61
3.07
8.5
8.0
7.5
7.0
6.5
6.0
5.5
5.0
4.5
4.0
3.5
3.0
2.5
2.0
1.5
1.0
Chemical Shift (ppm)

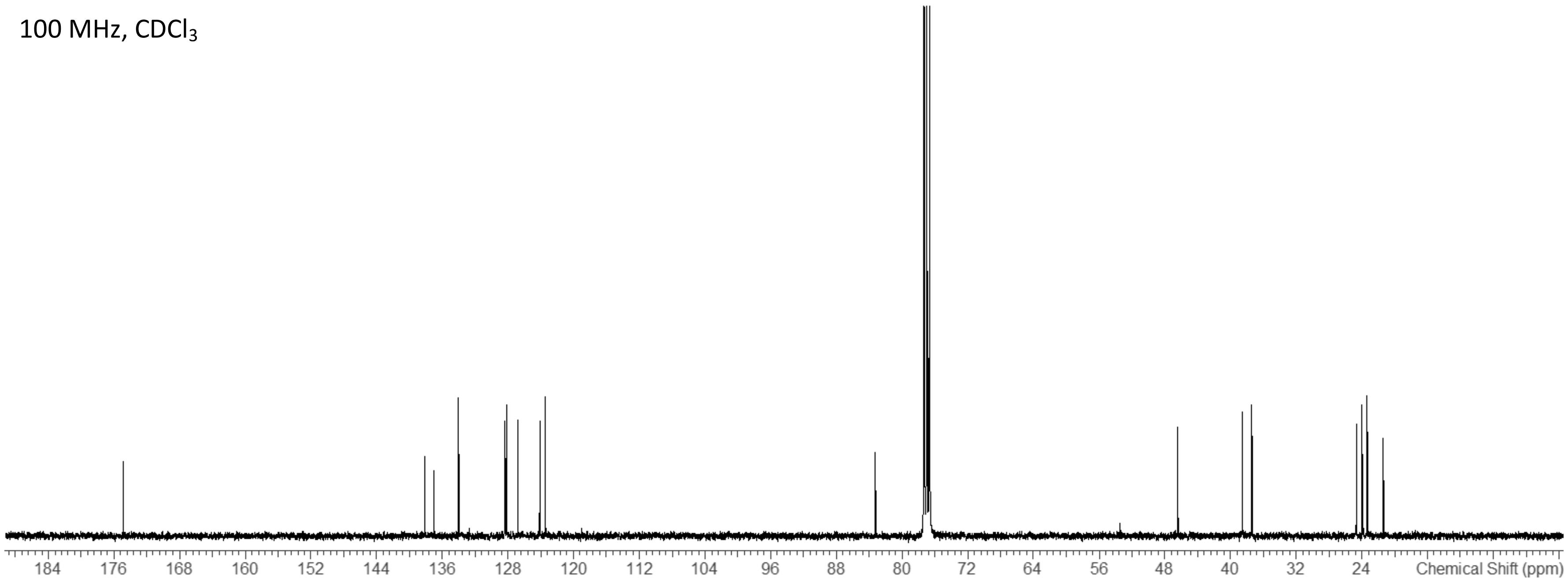
100 MHz, CDCl3
184
176
168
160
152
144
136
128
120
112
104
96
88
80
72
64
56
48
40
32
24
Chemical Shift (ppm)

400 MHz, $CDCl_3$

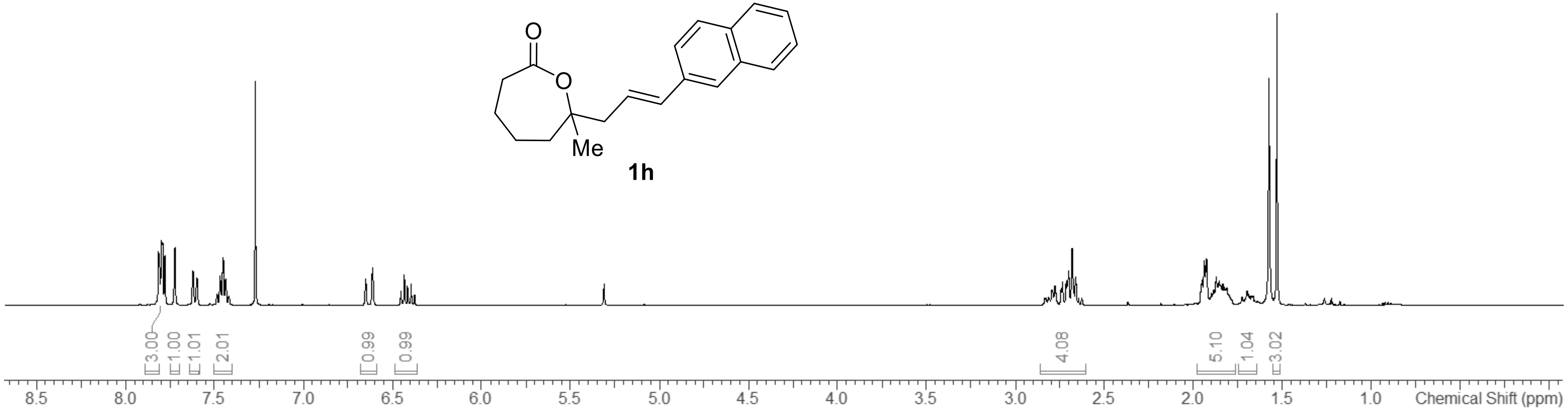

**1h**


100 MHz, $CDCl_3$

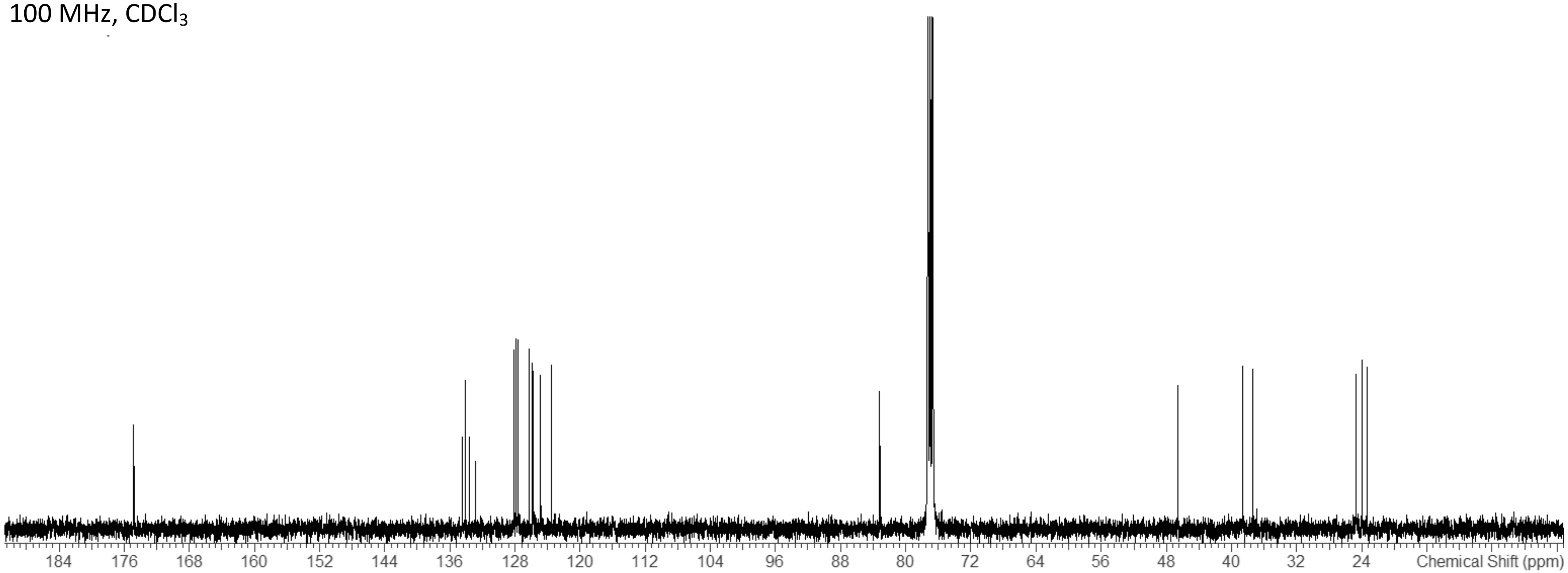

400 MHz, $CDCl_3$

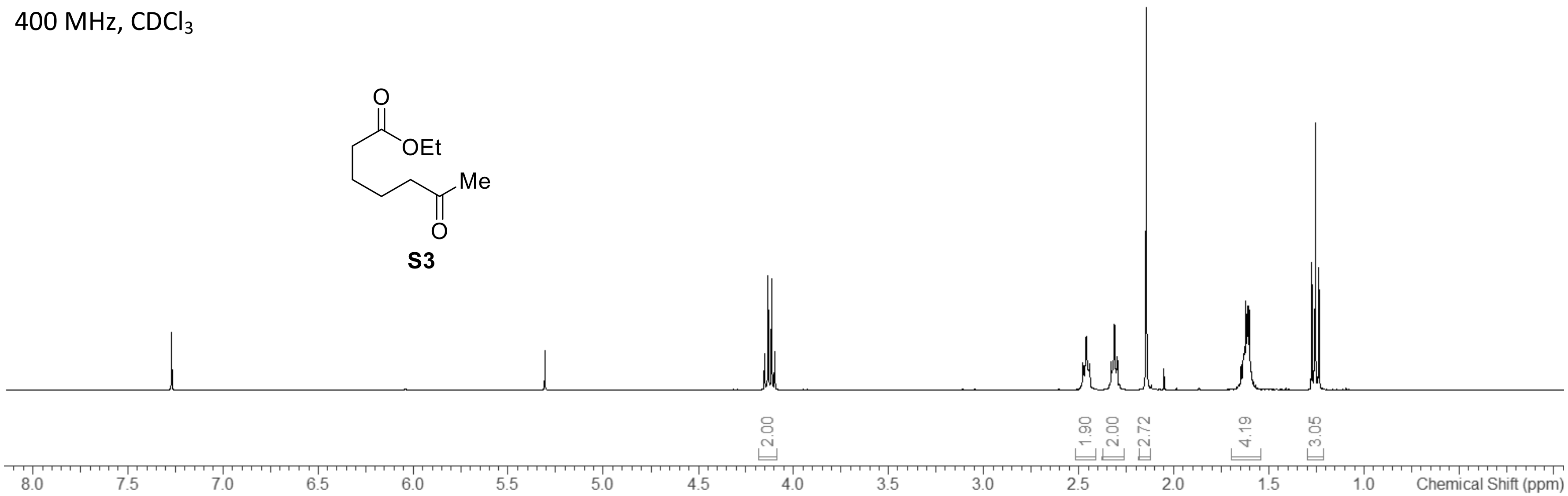


100 MHz, $CDCl_3$

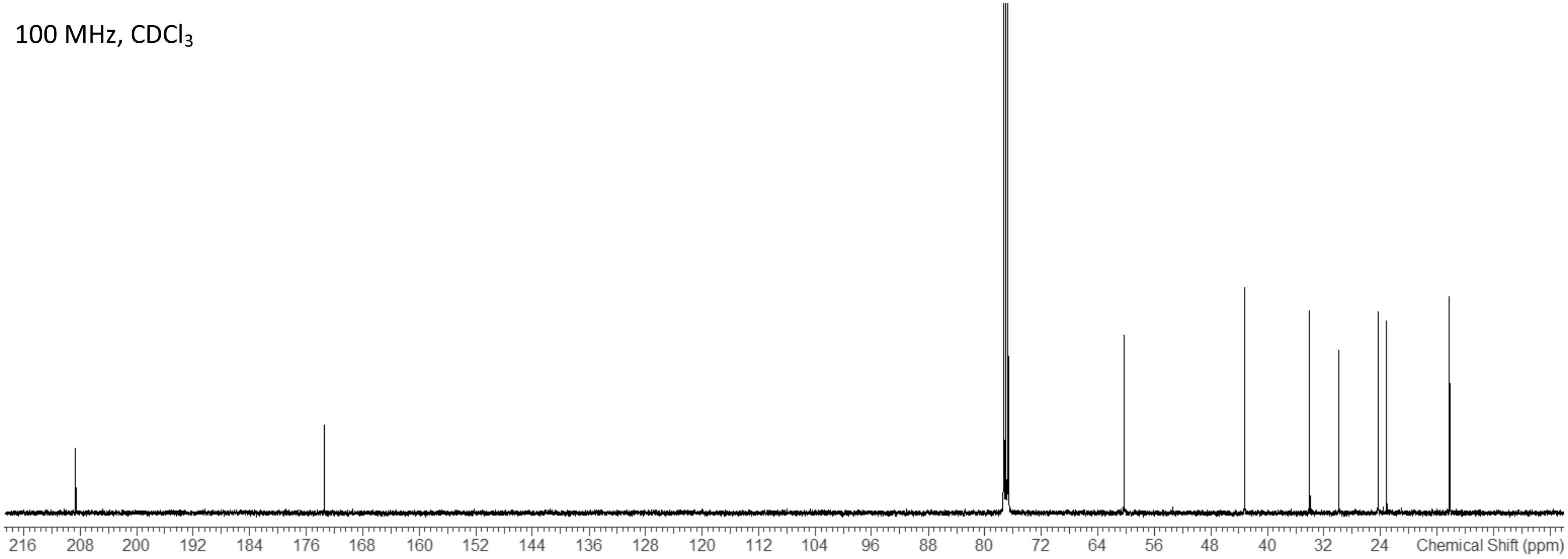

400 MHz, $CDCl_3$

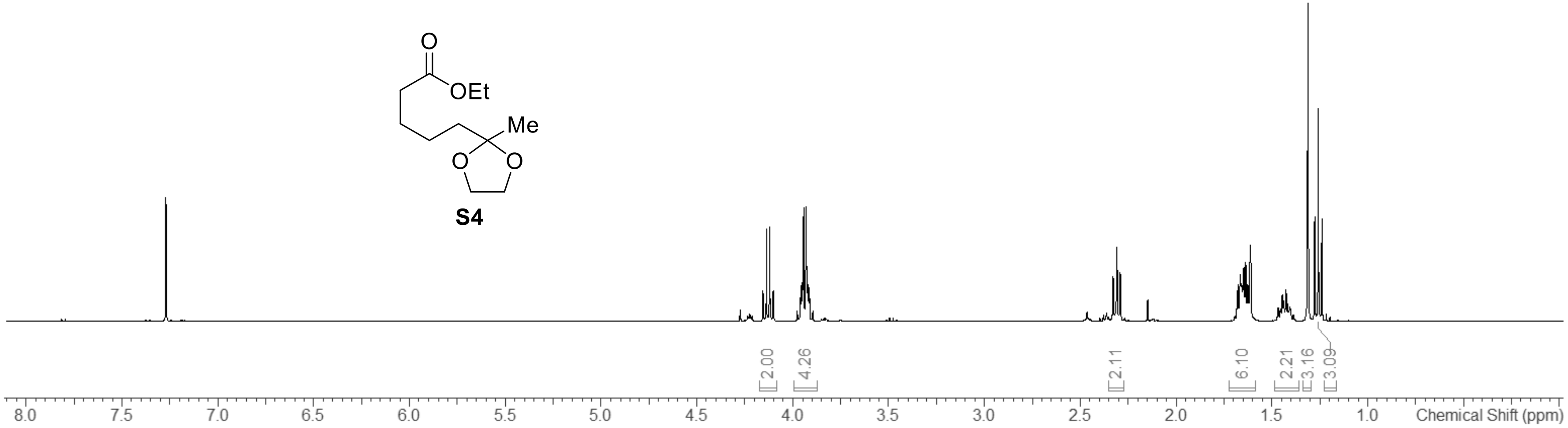


100 MHz, $CDCl_3$

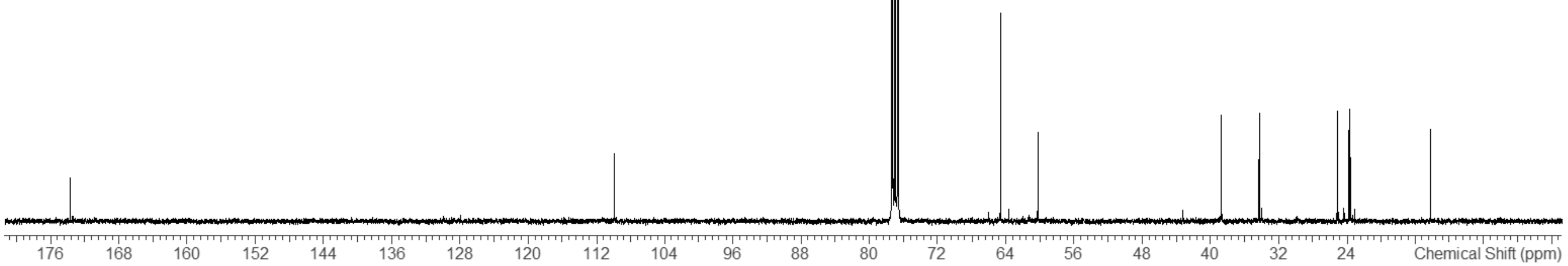

400 MHz, $CDCl_3$

**S5**

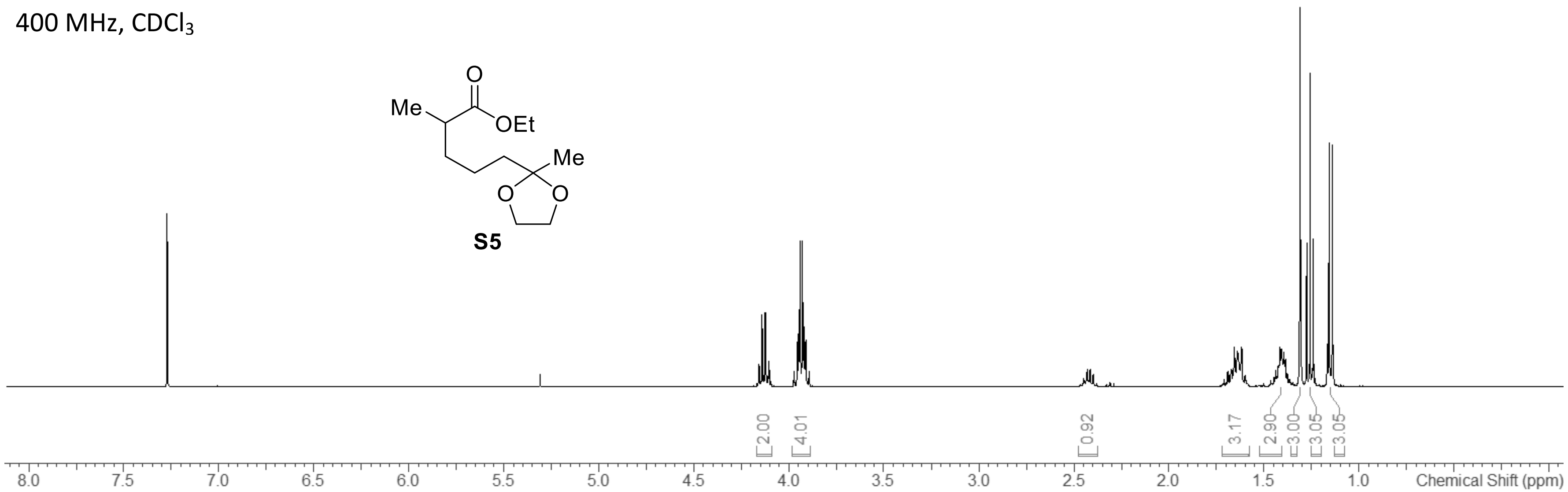


100 MHz, $CDCl_3$

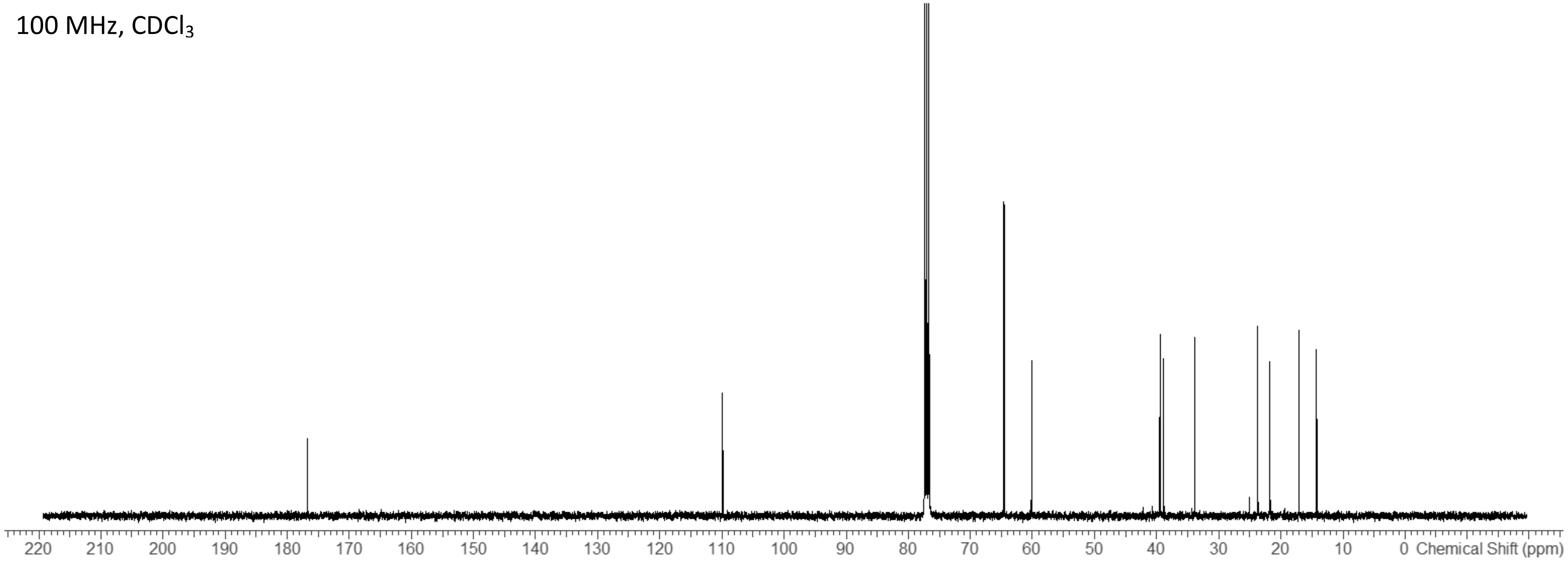

400 MHz, $CDCl_3$

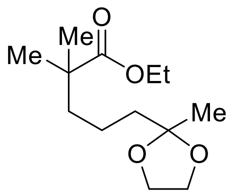


**S6**

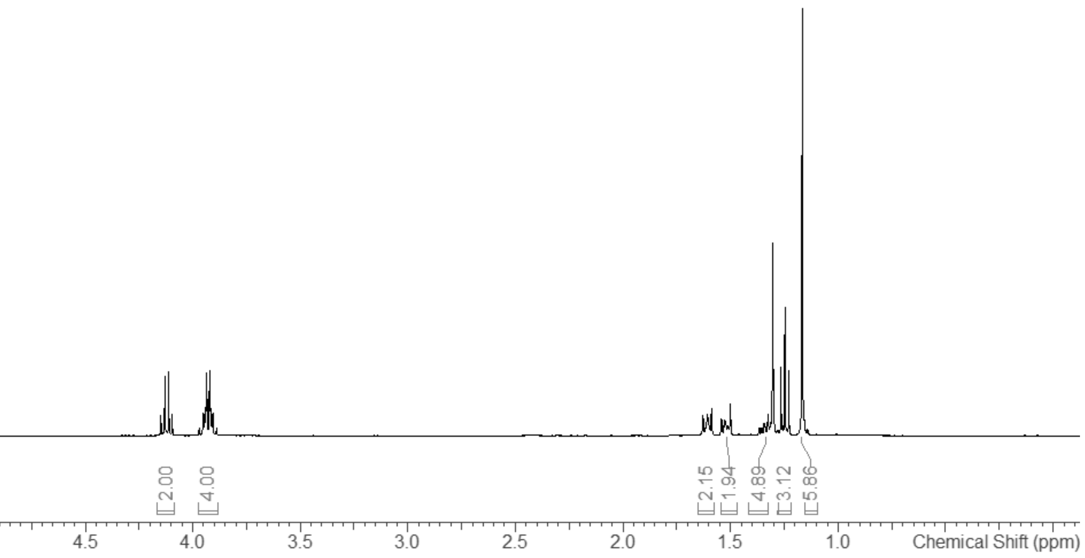


100 MHz, $CDCl_3$

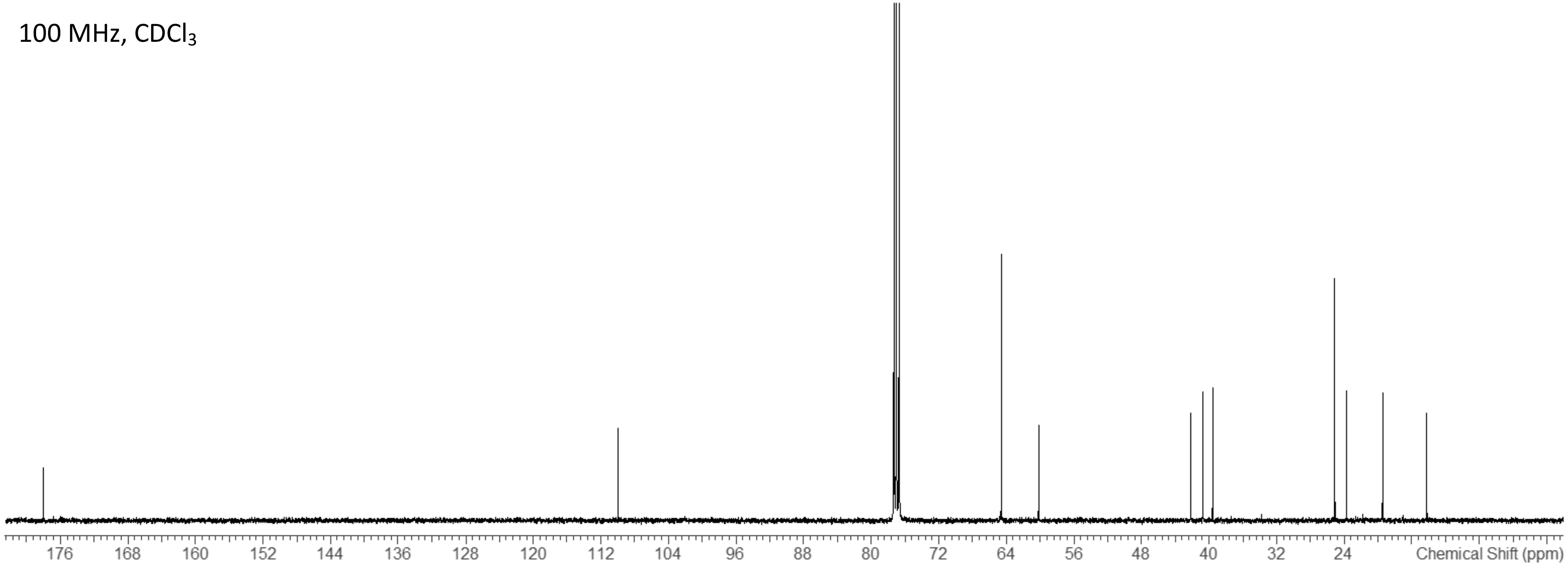

400 MHz, $CDCl_3$

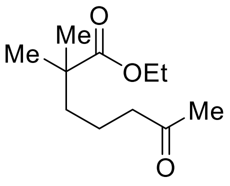


**S7**

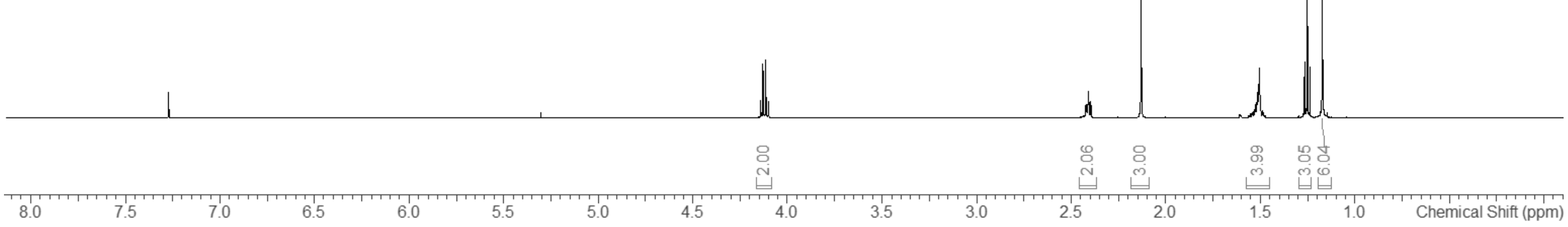


100 MHz, $CDCl_3$

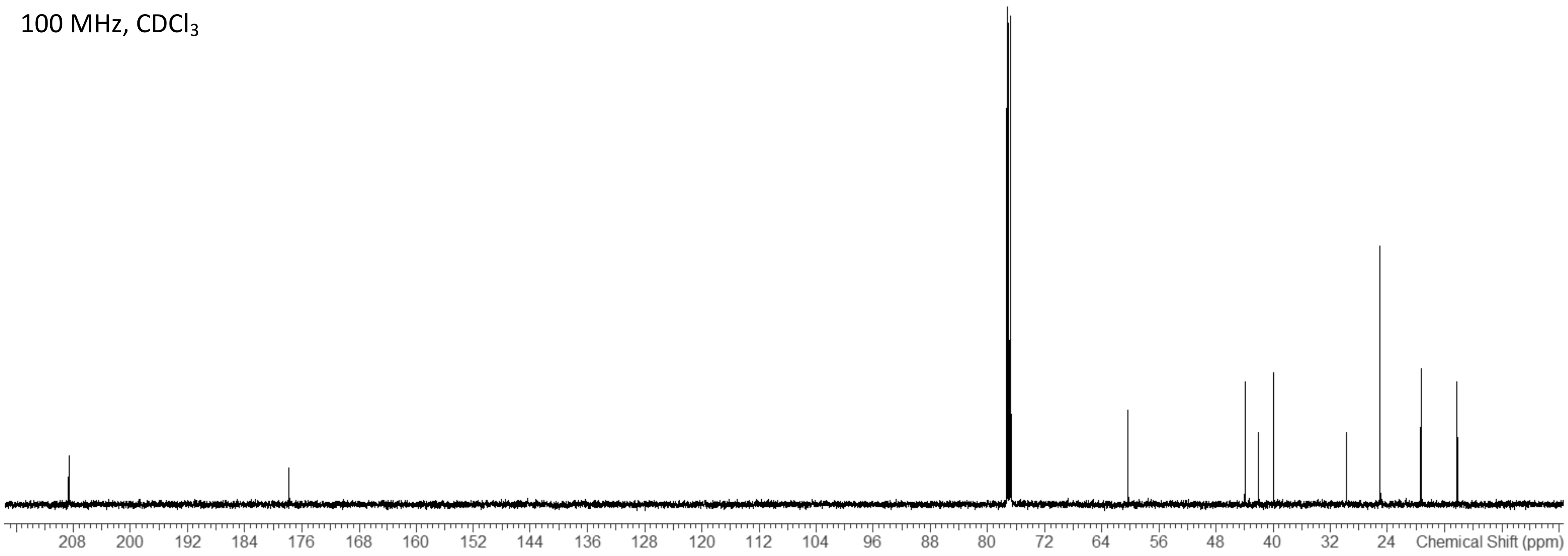

400 MHz, $CDCl_3$

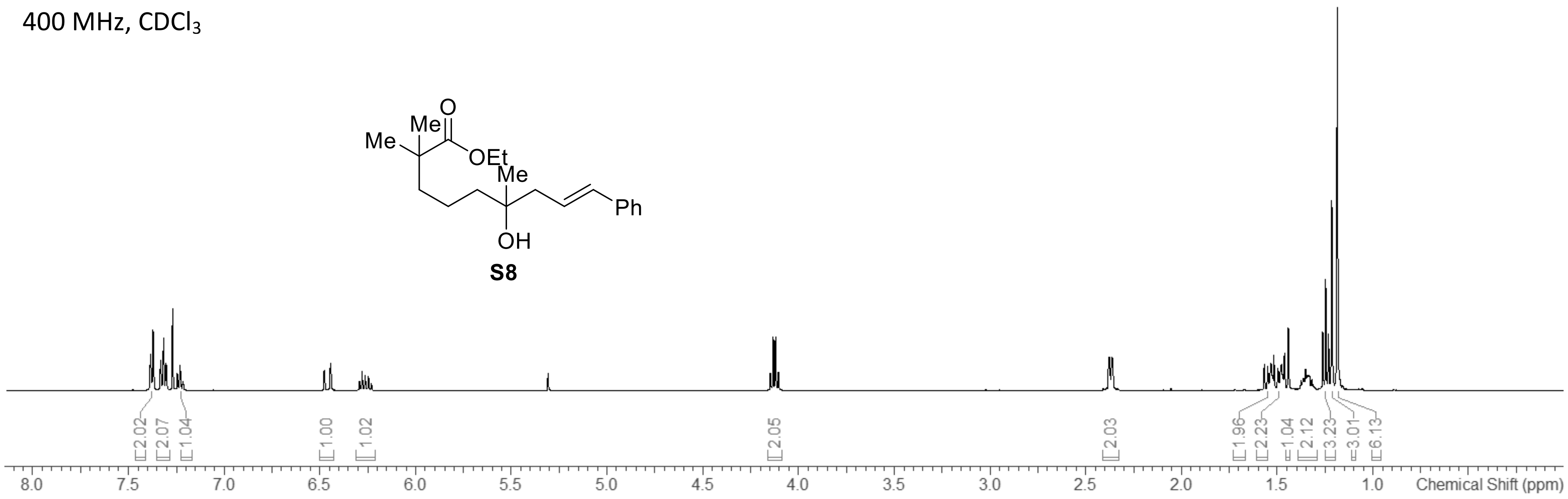


**S8**

100 MHz, $CDCl_3$

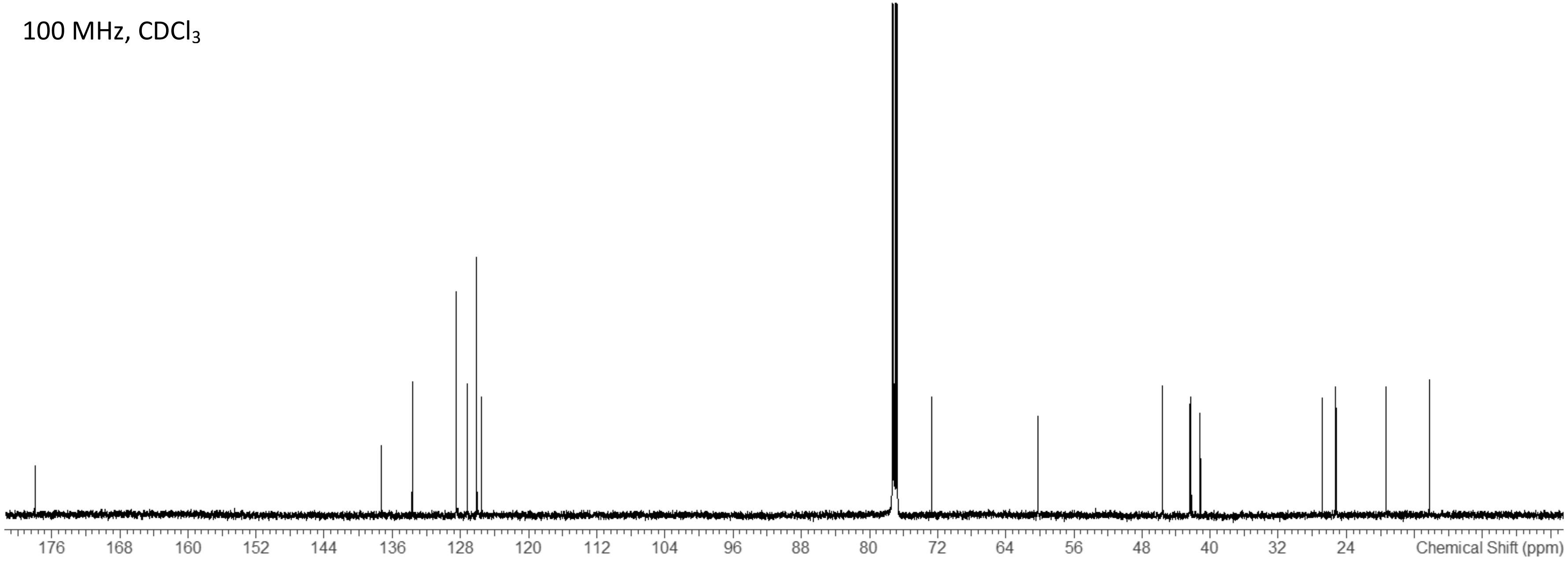

400 MHz, $CDCl_3$

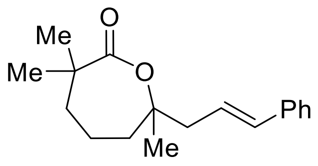

**1i**

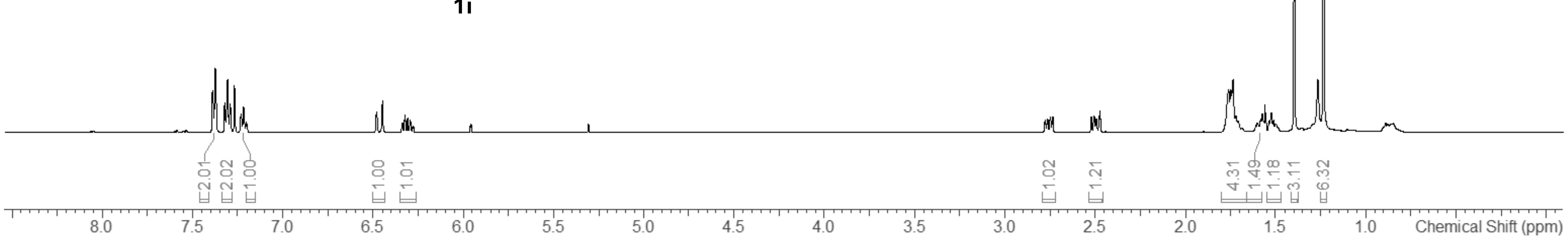


100 MHz, $CDCl_3$

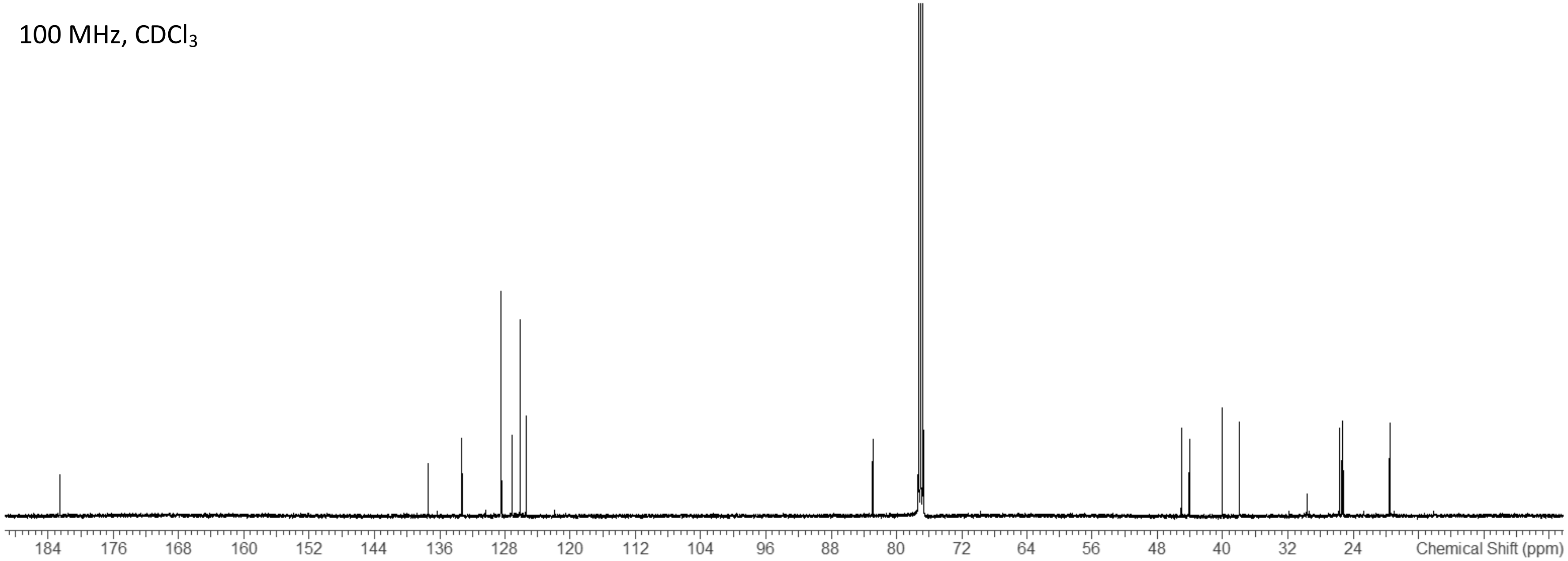

400 MHz, $CDCl_3$

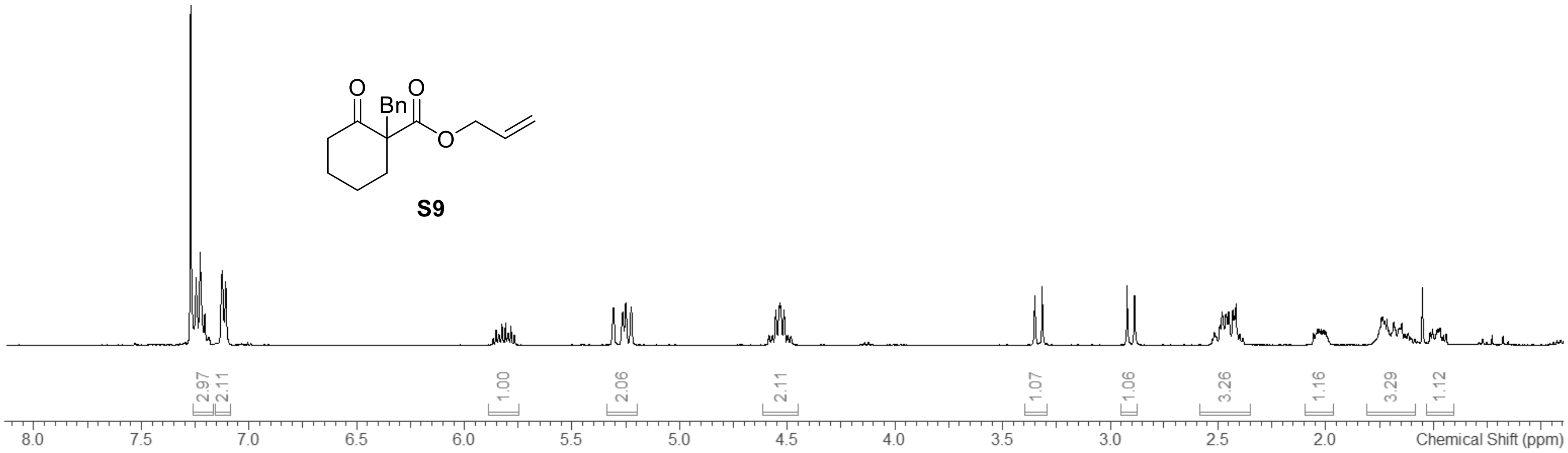


**S9**

100 MHz, $CDCl_3$

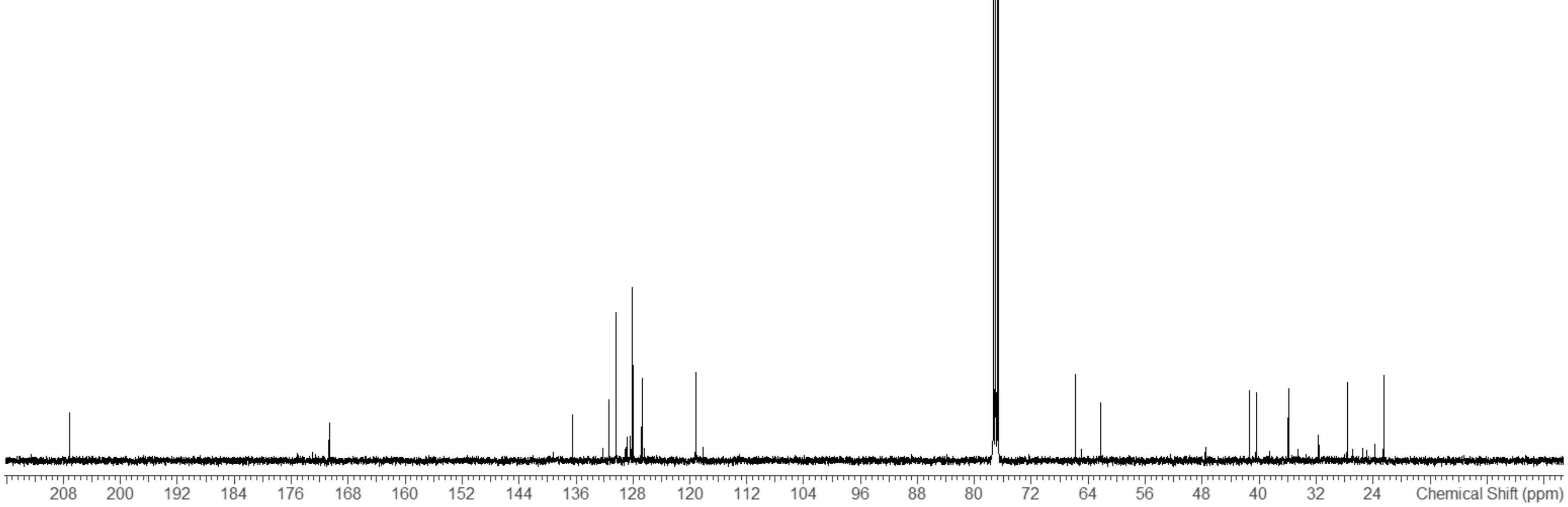

400 MHz, $CDCl_3$

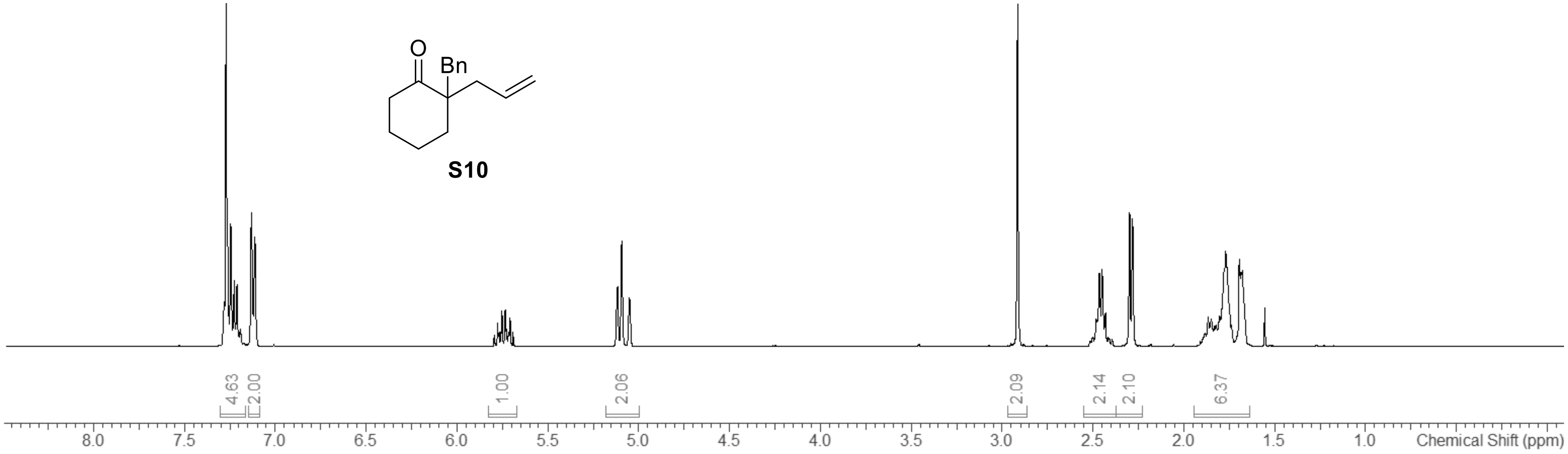
S10


100 MHz, $CDCl_3$

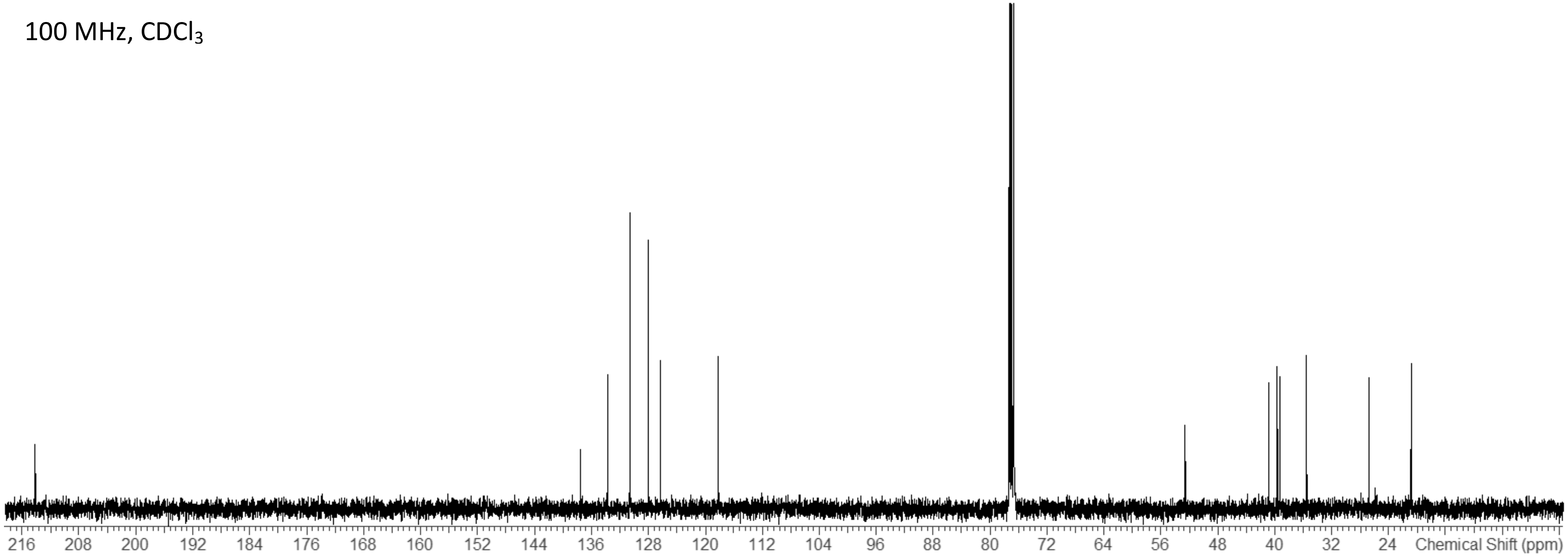

400 MHz, $CDCl_3$

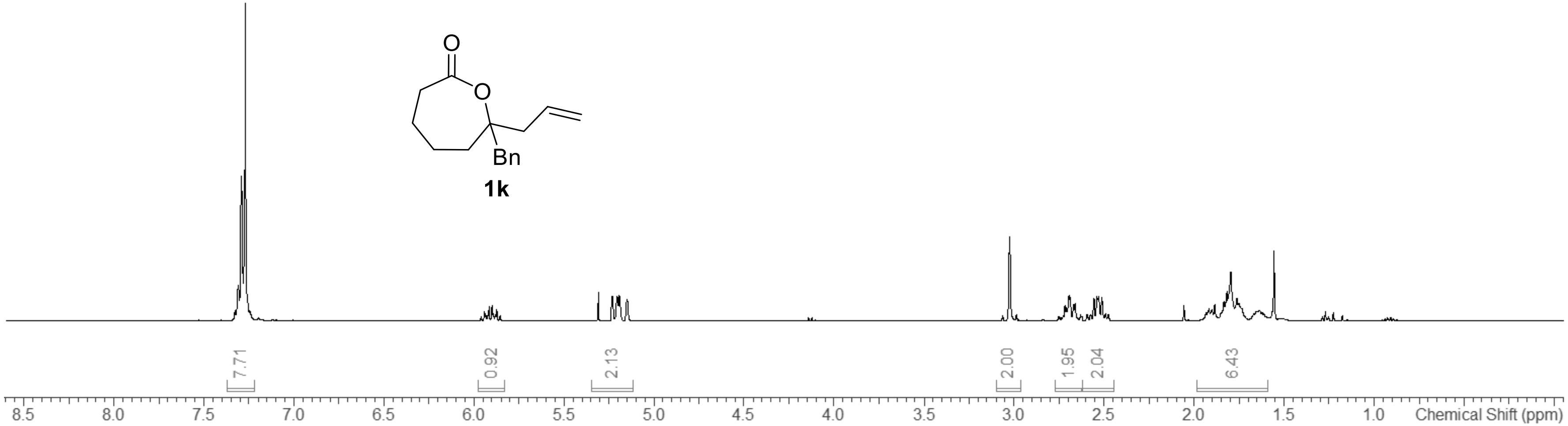


100 MHz, $CDCl_3$

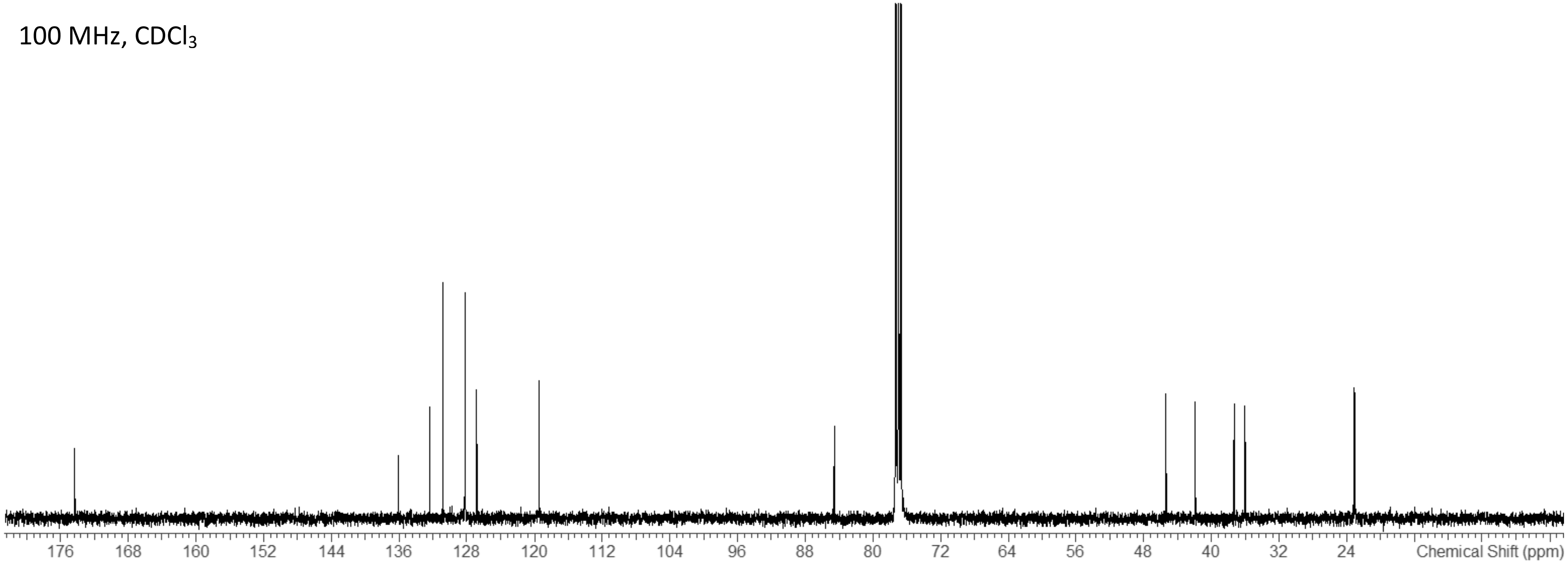

400 MHz, $CDCl_3$

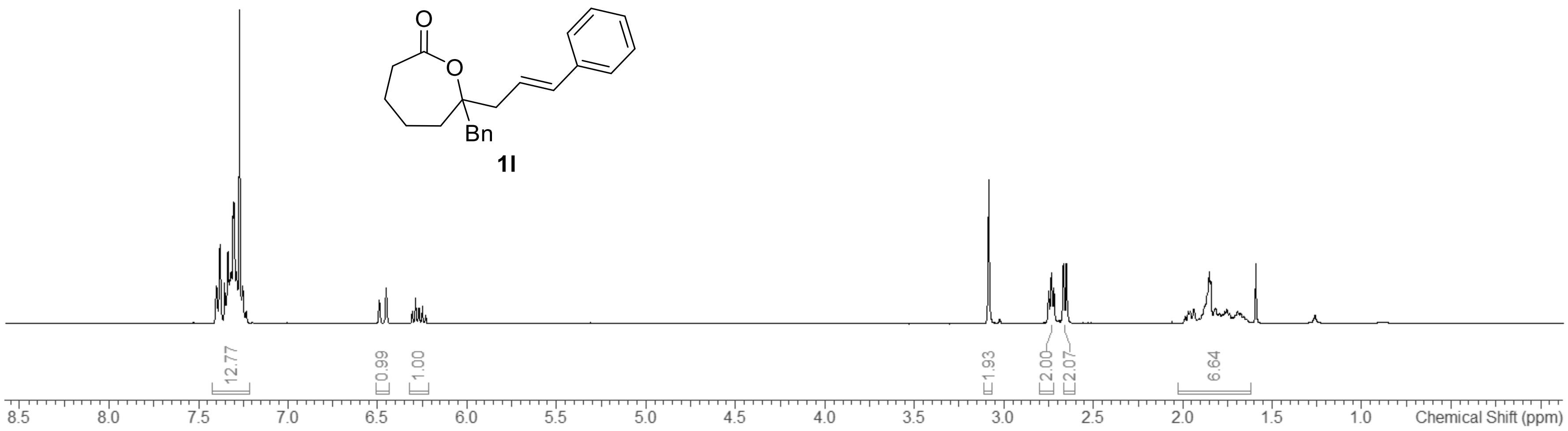


100 MHz, $CDCl_3$

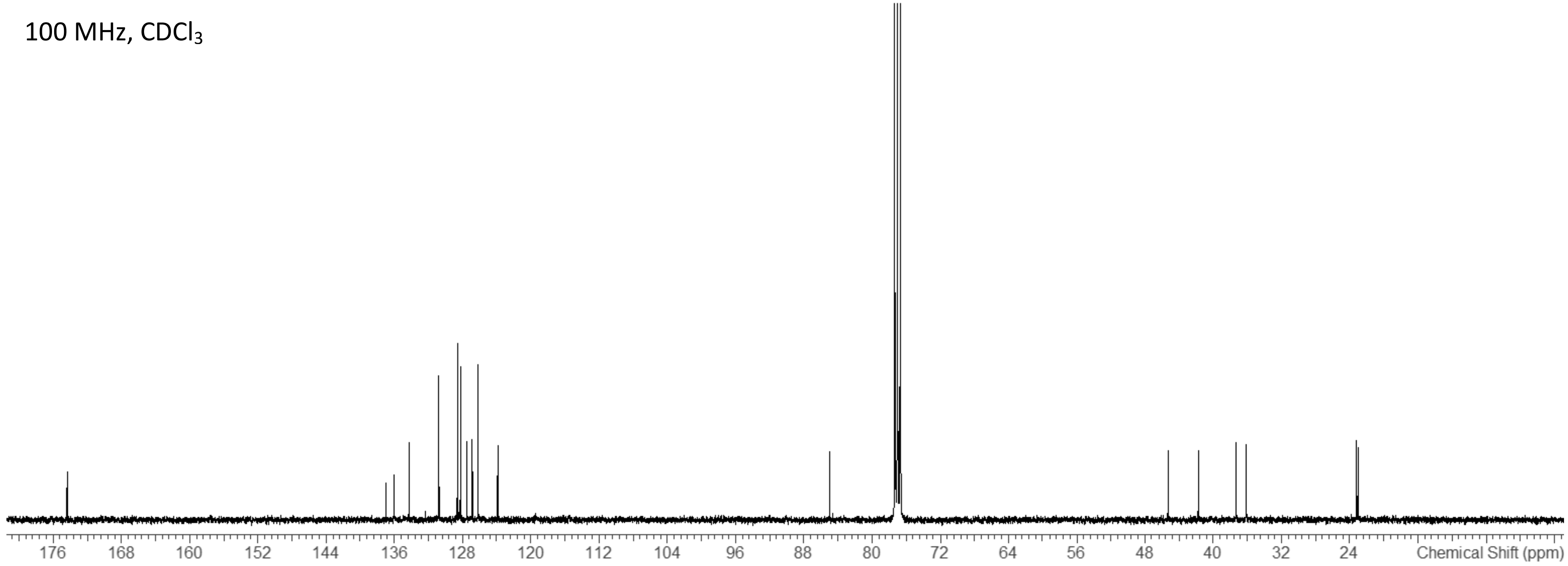

400 MHz, $CDCl_3$

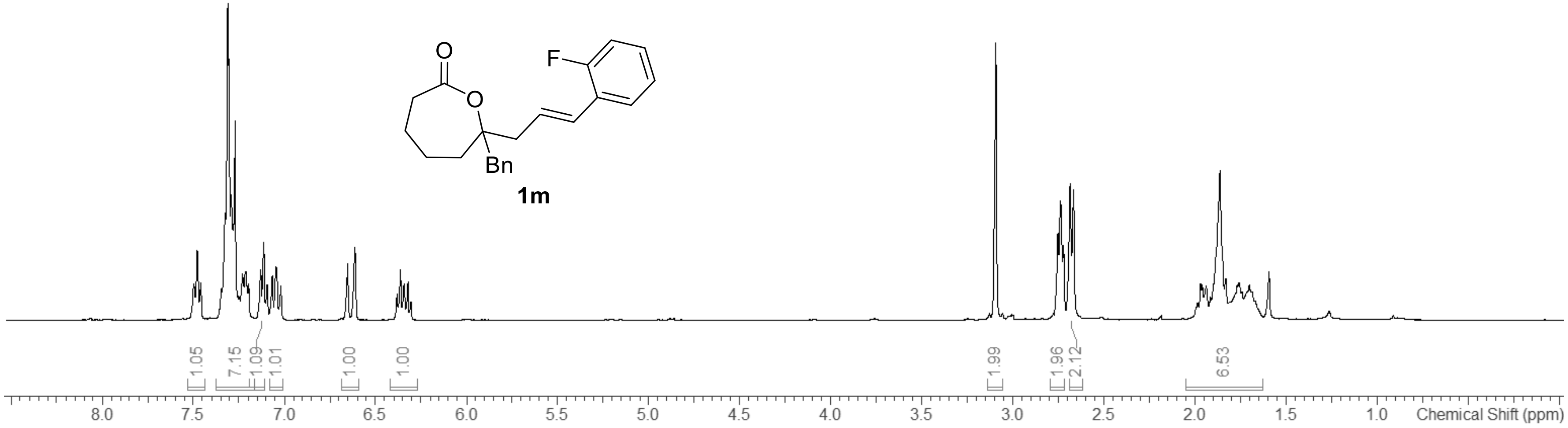


100 MHz, $CDCl_3$

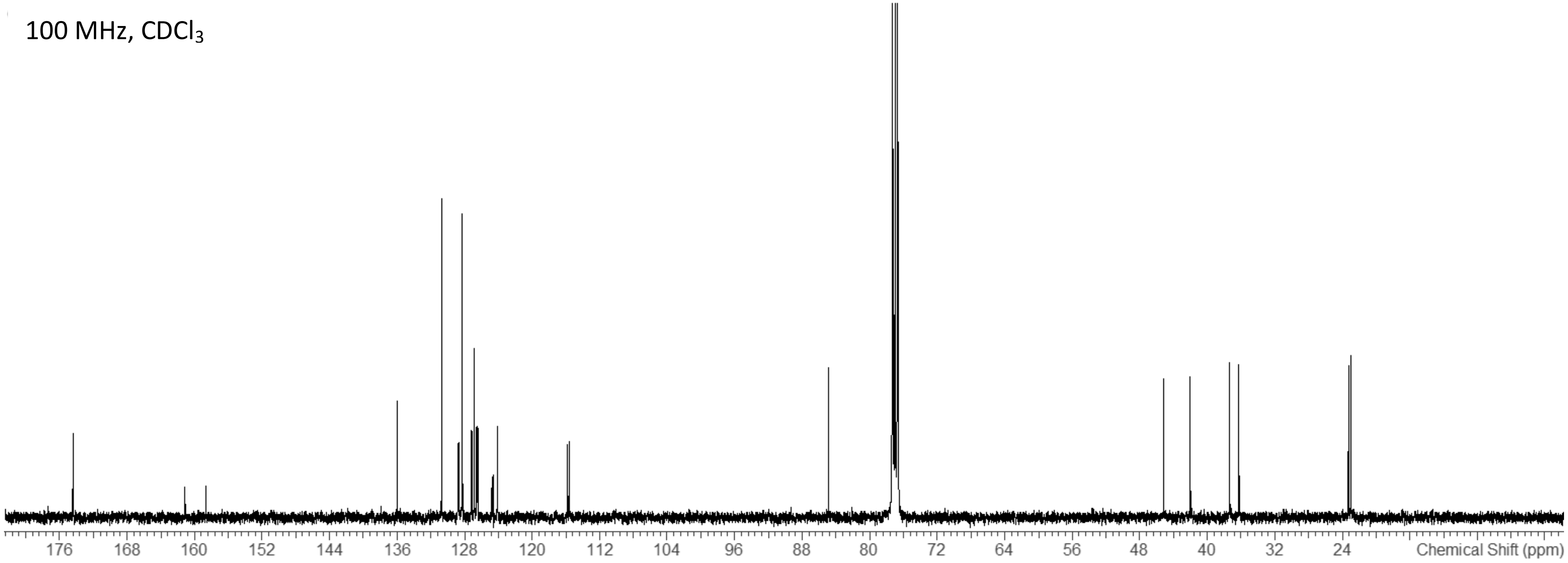

400 MHz, $CDCl_3$

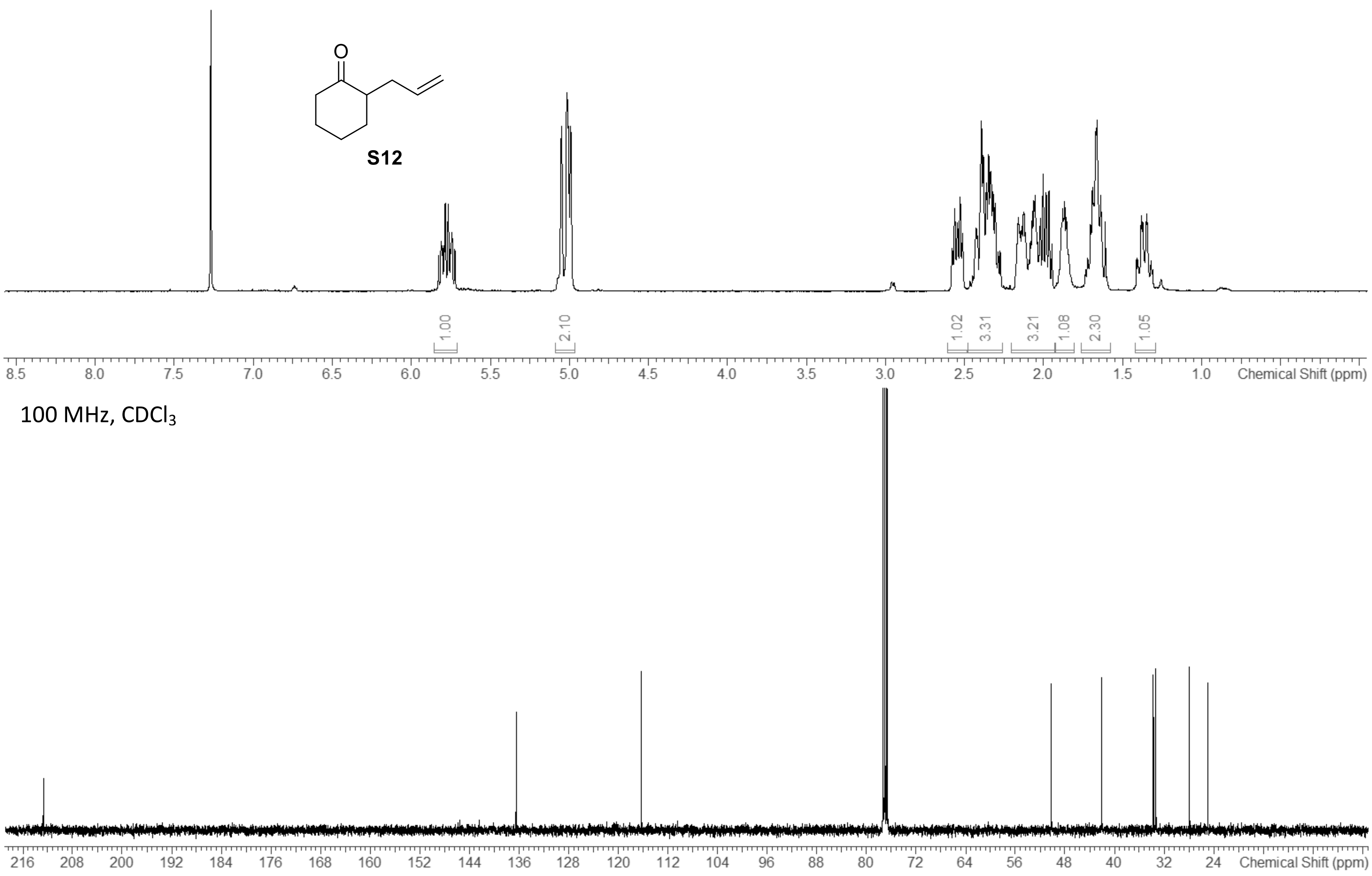

400 MHz, $CDCl_3$

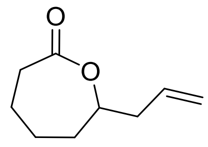

**S13**

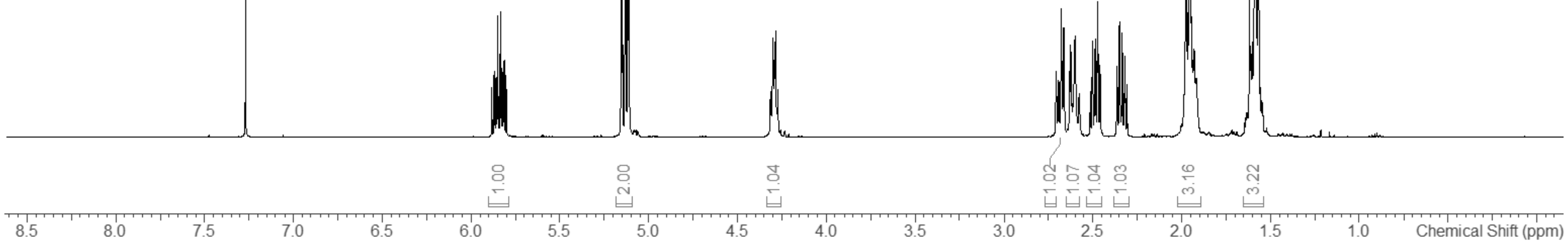


100 MHz, $CDCl_3$

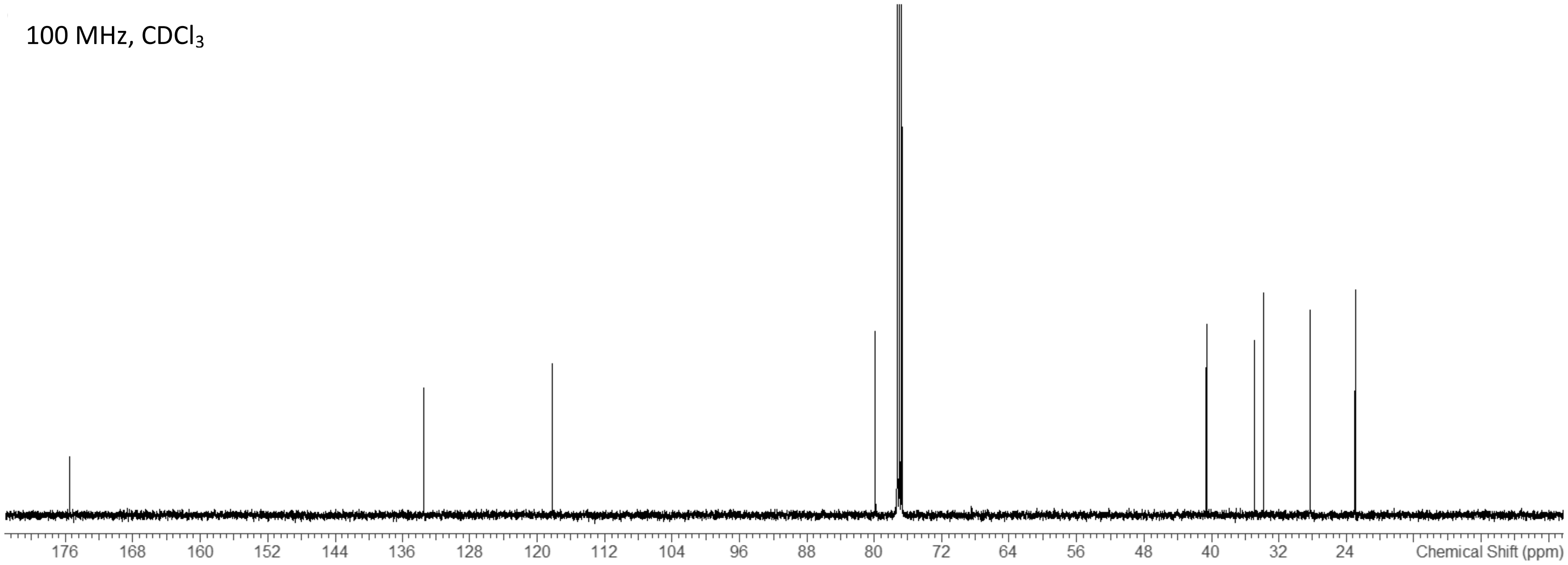

400 MHz, $CDCl_3$

**1n**

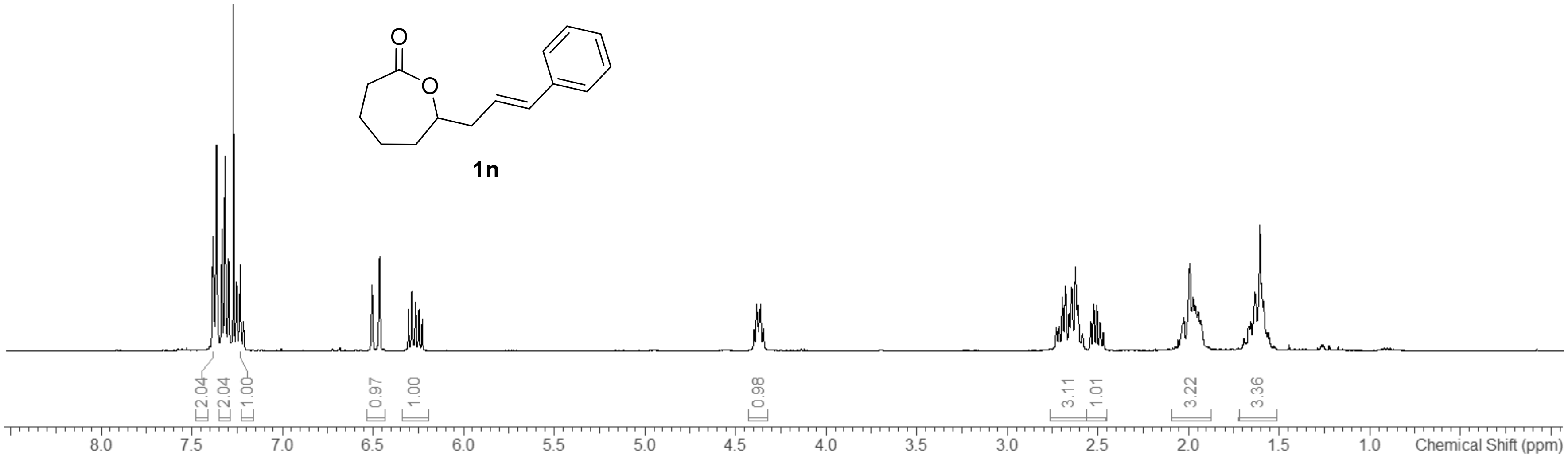


100 MHz, $CDCl_3$

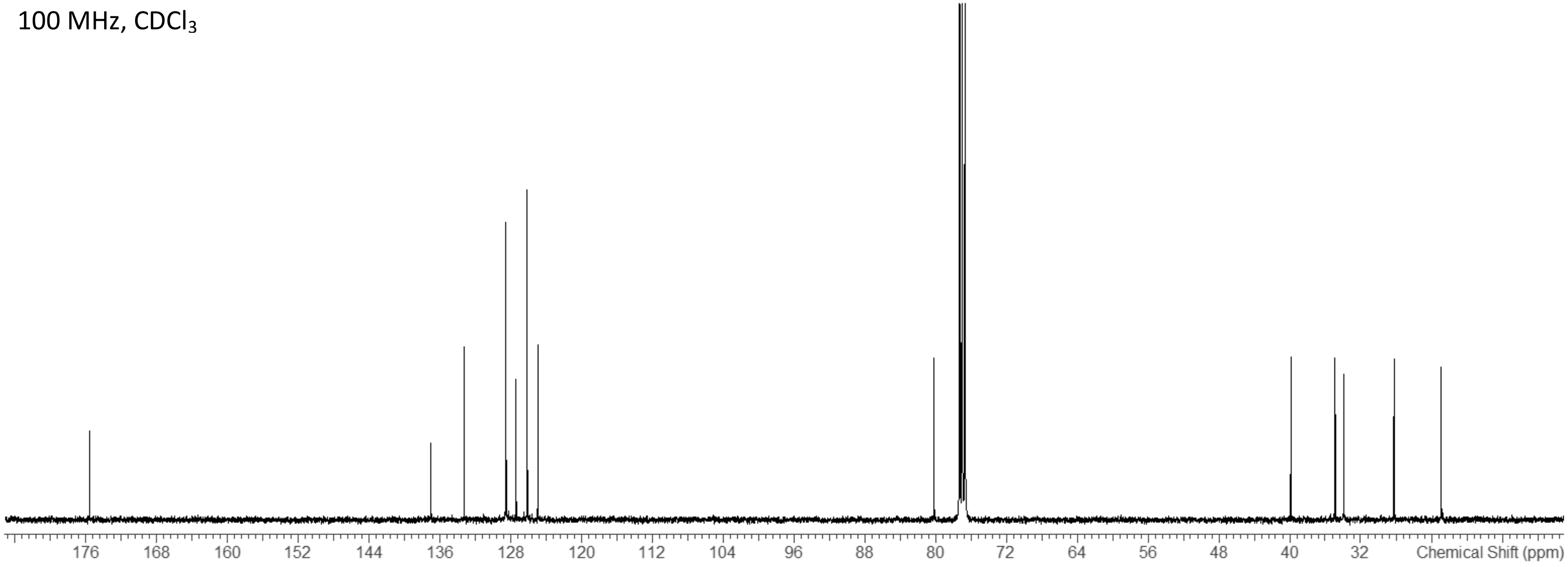

400 MHz, $CDCl_3$

**1o**

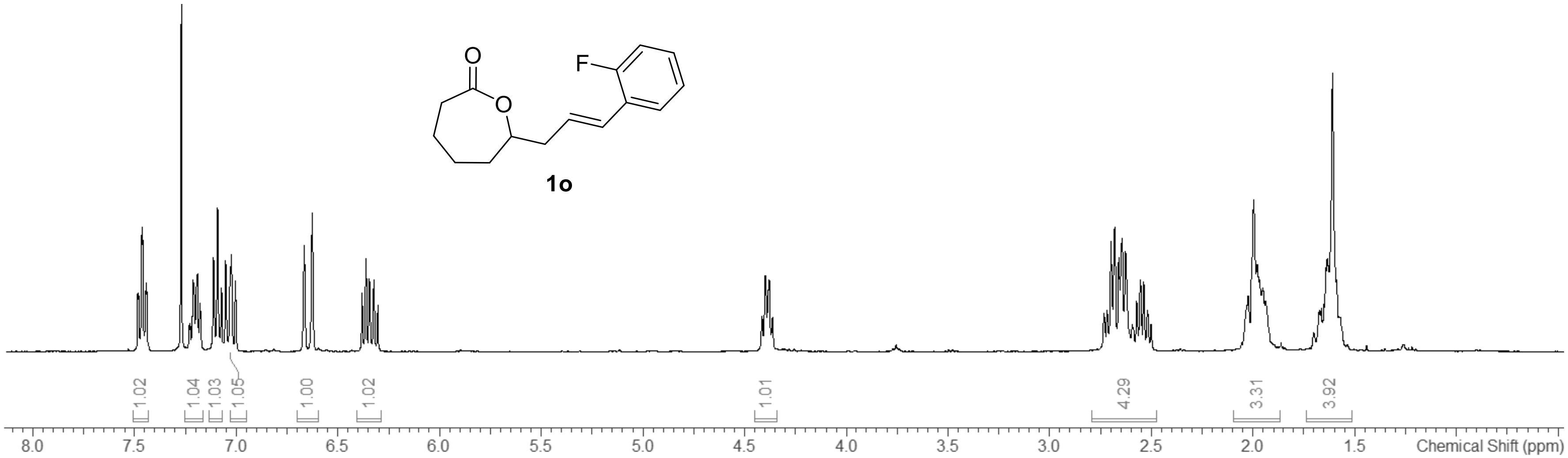


100 MHz, $CDCl_3$

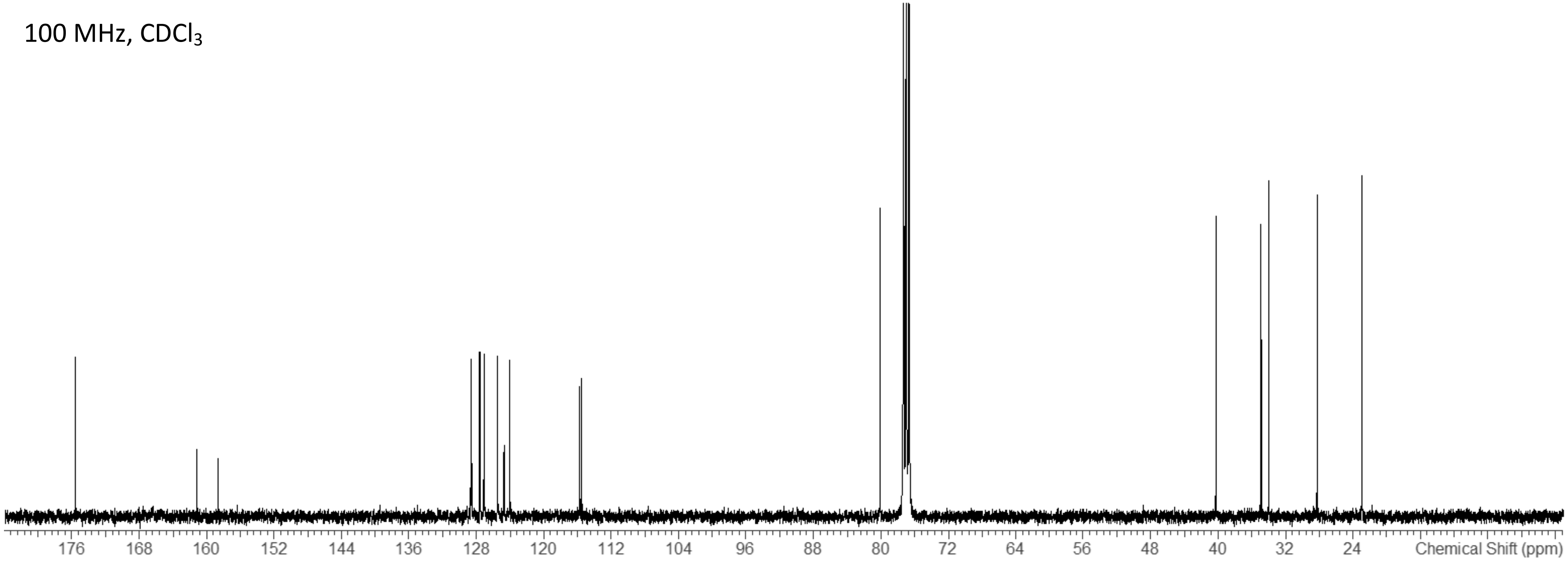

400 MHz, $CDCl_3$

**3a**

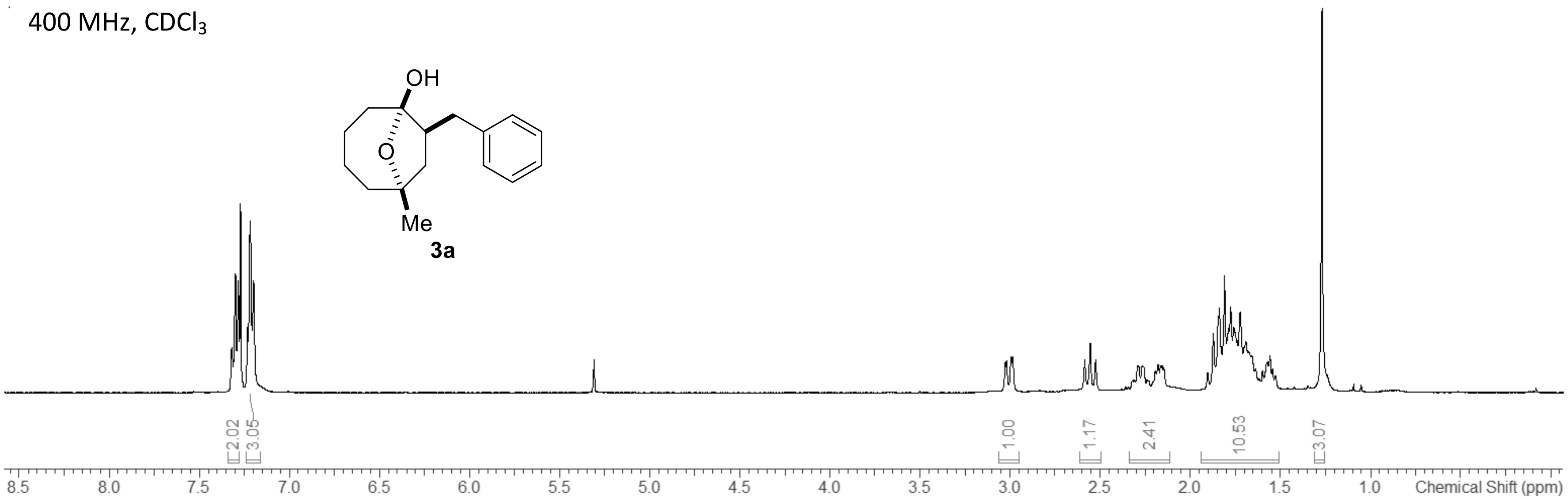


100 MHz, $CDCl_3$

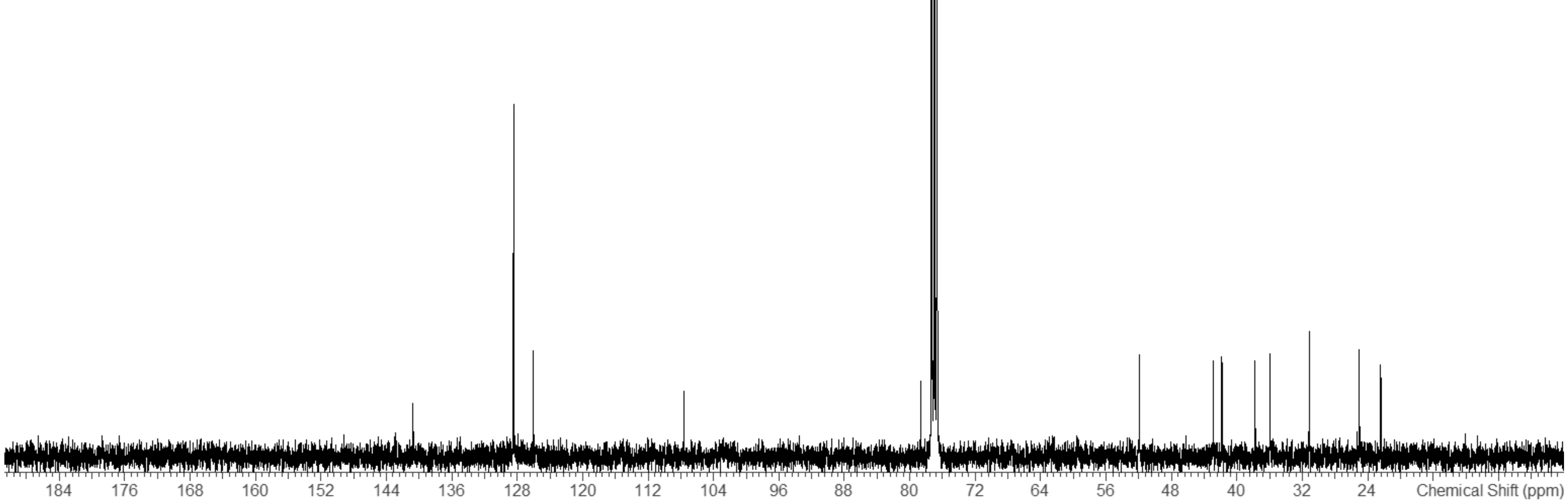

400 MHz, $CDCl_3$

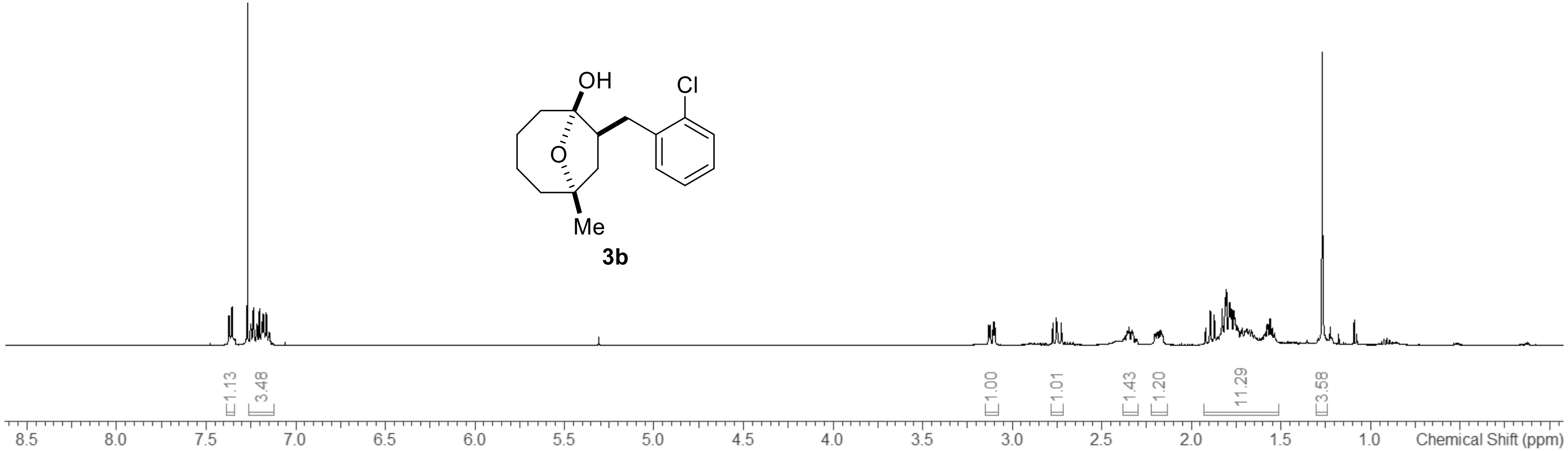


100 MHz, $CDCl_3$

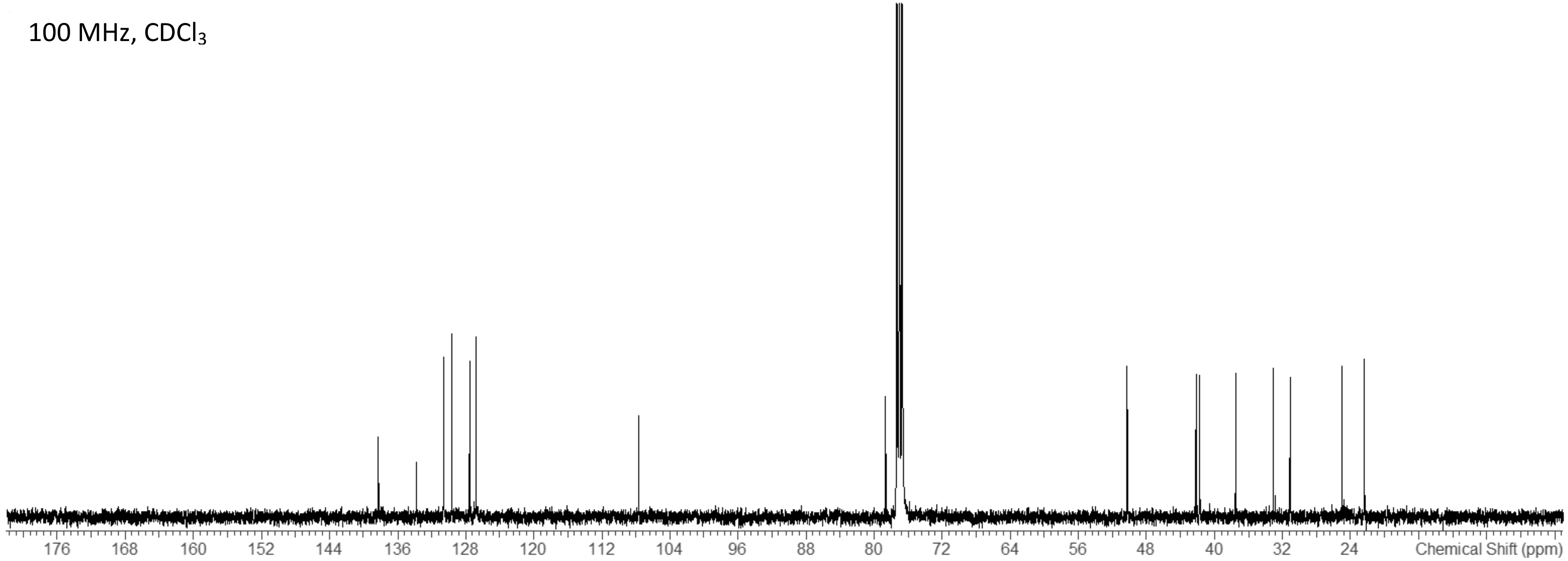

400 MHz, $CDCl_3$

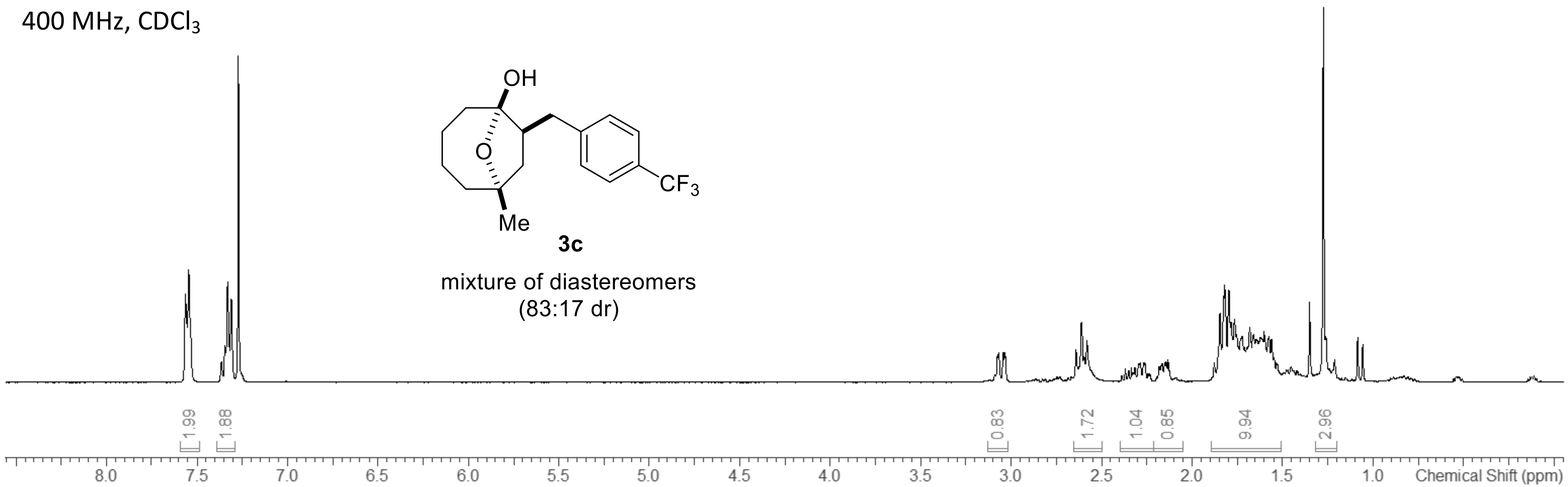


**3c**

mixture of diastereomers
(83:17 dr)

100 MHz, $CDCl_3$

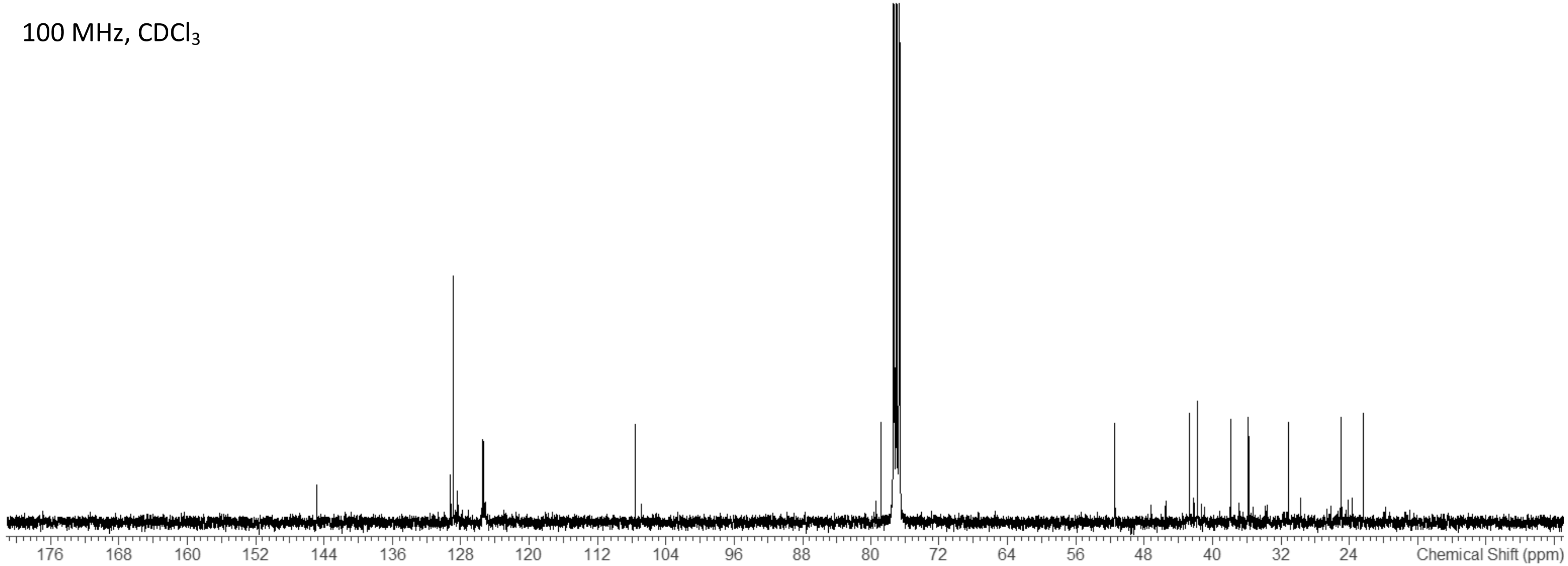

400 MHz, $CDCl_3$

**3d**

mixture of diastereomers
(80:20 dr)

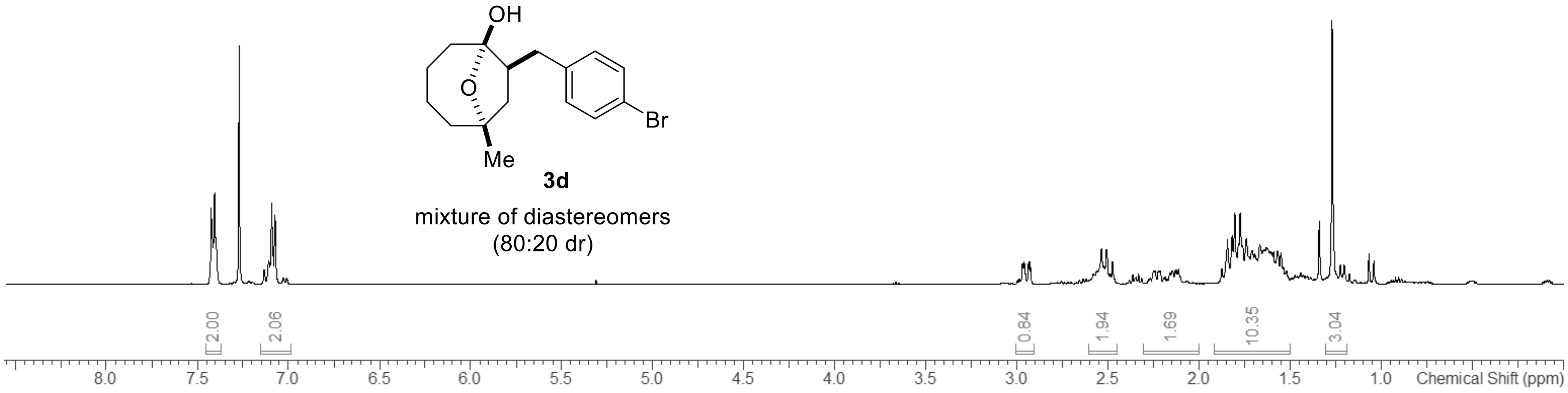


100 MHz, $CDCl_3$

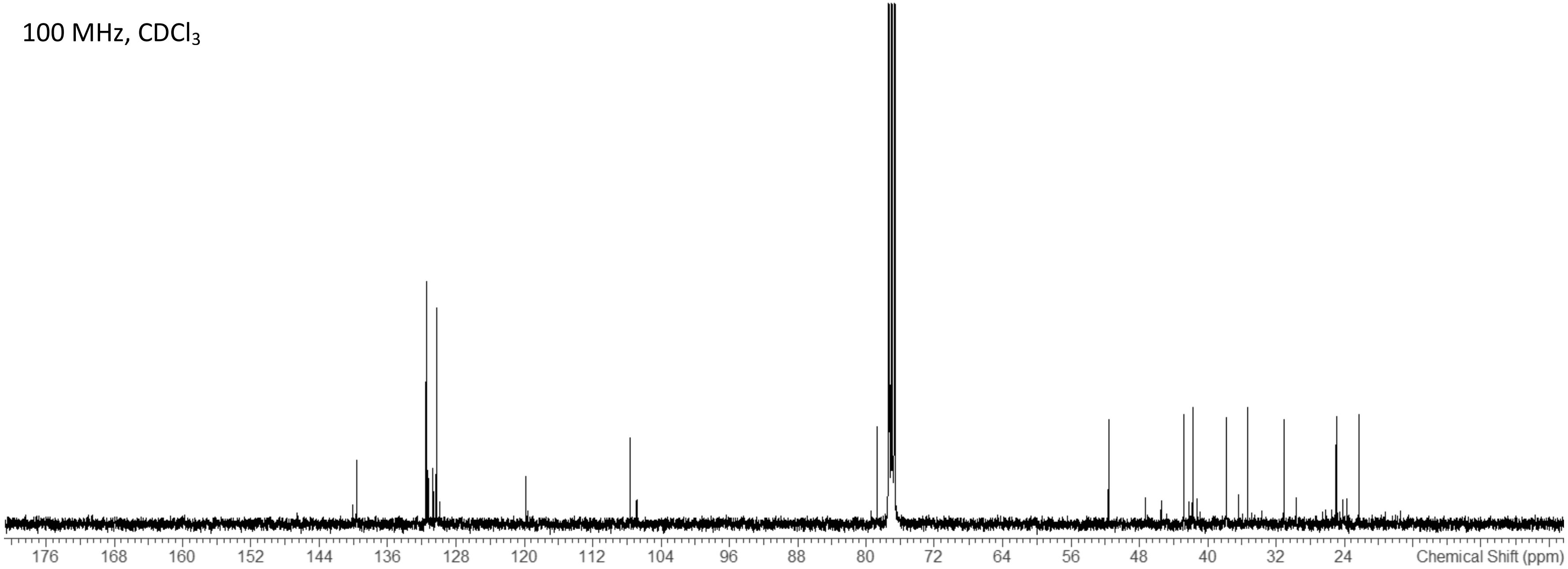

400 MHz, $CDCl_3$

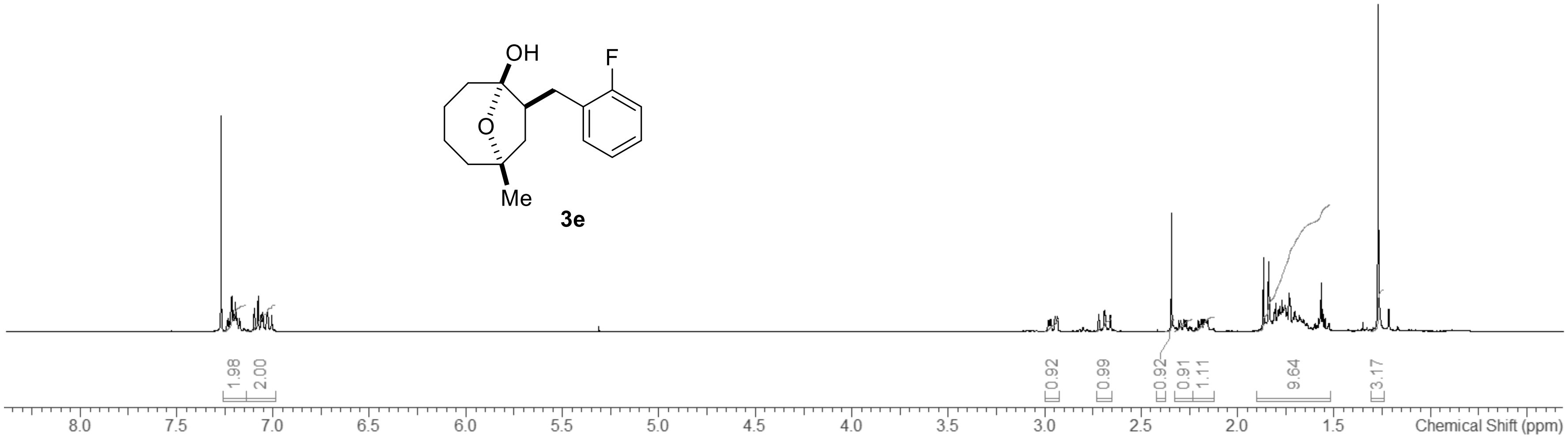


**3e**

100 MHz, $CDCl_3$

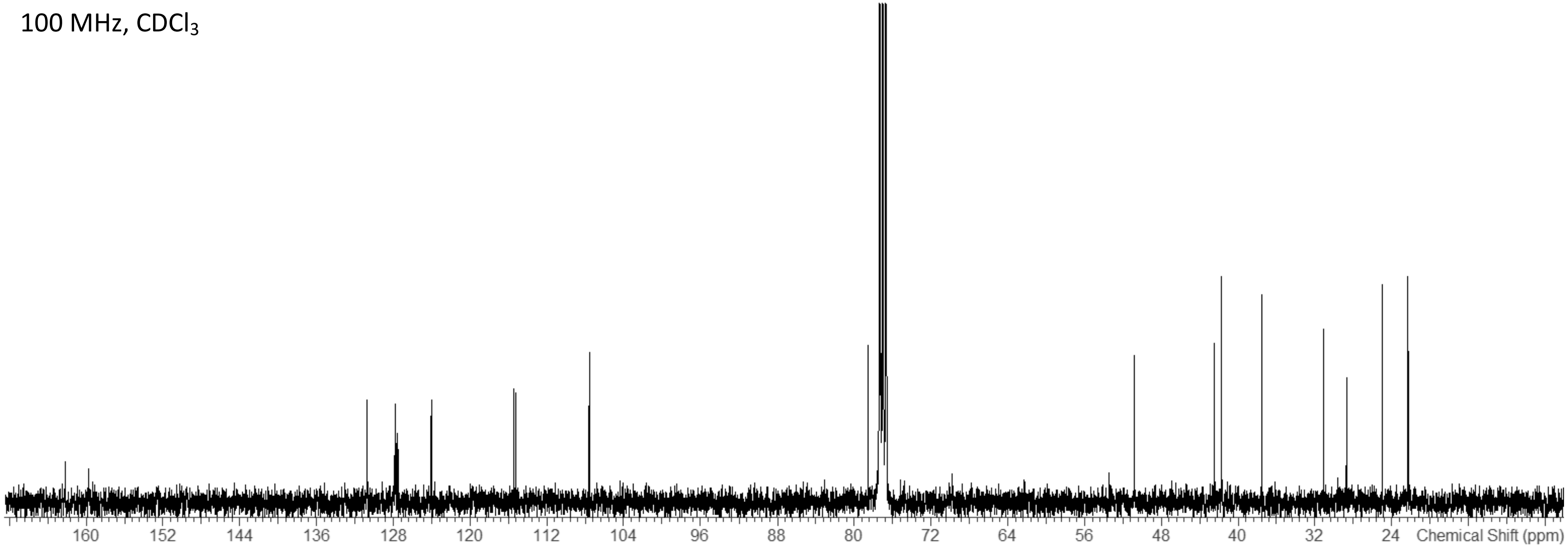

400 MHz, $CDCl_3$

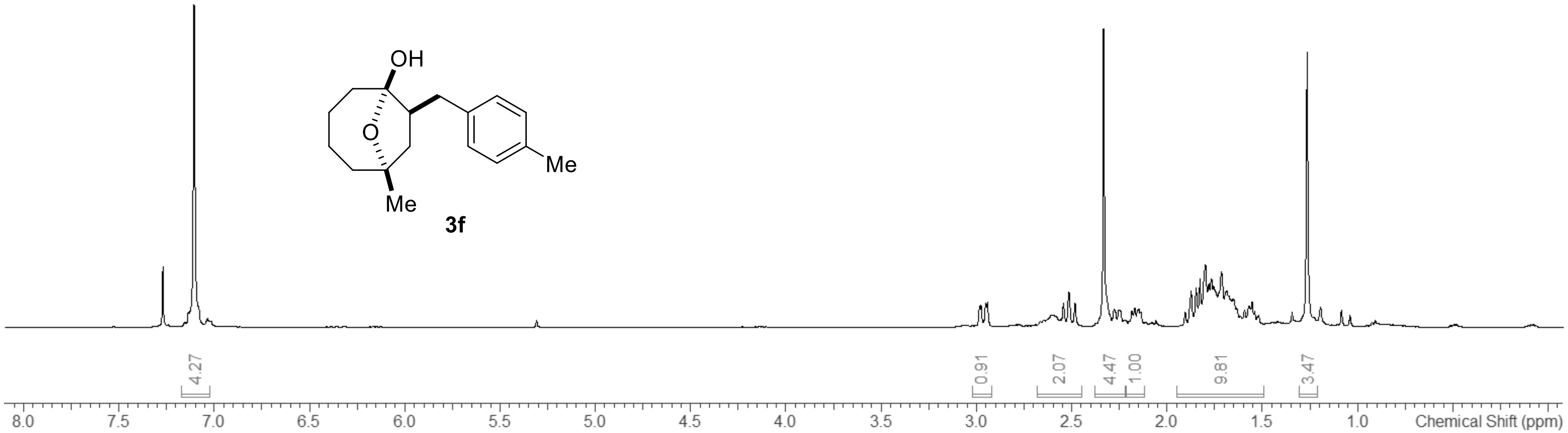


100 MHz, $CDCl_3$

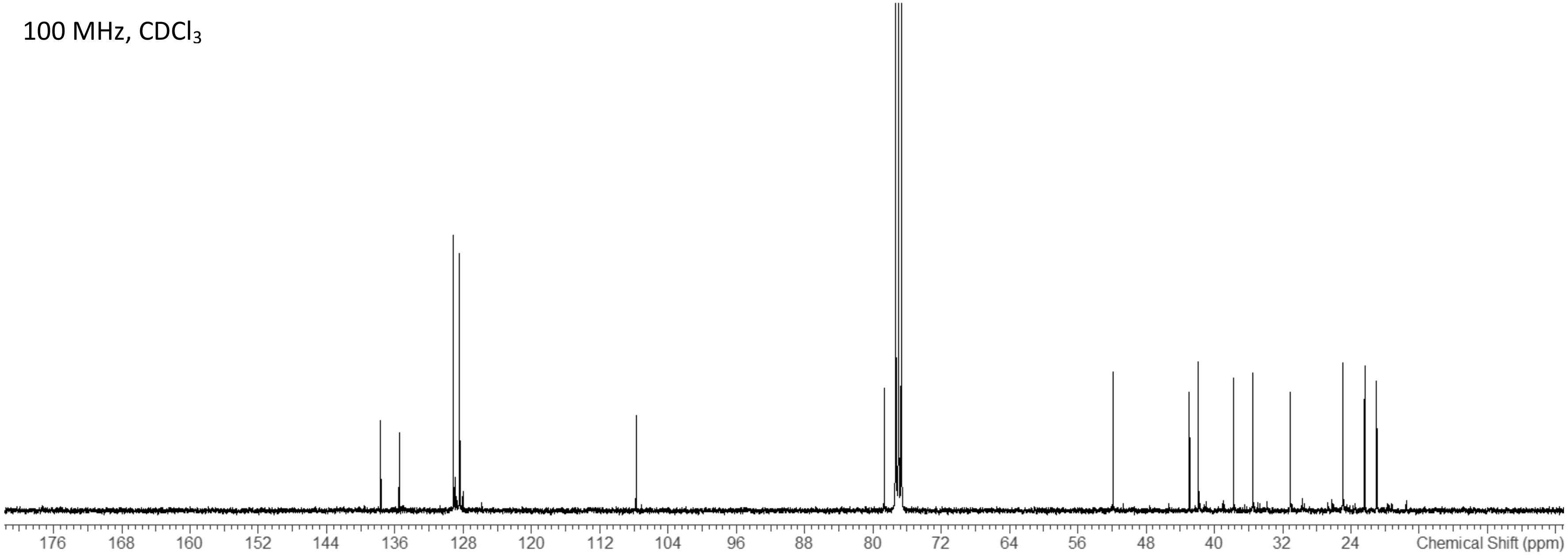

400 MHz, $CDCl_3$

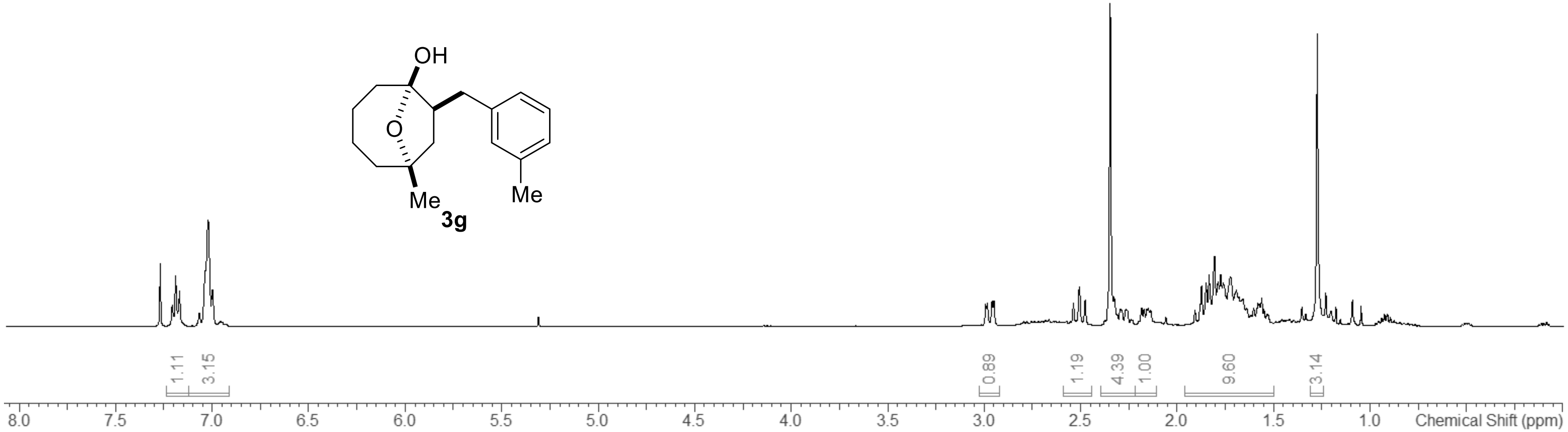


100 MHz, $CDCl_3$

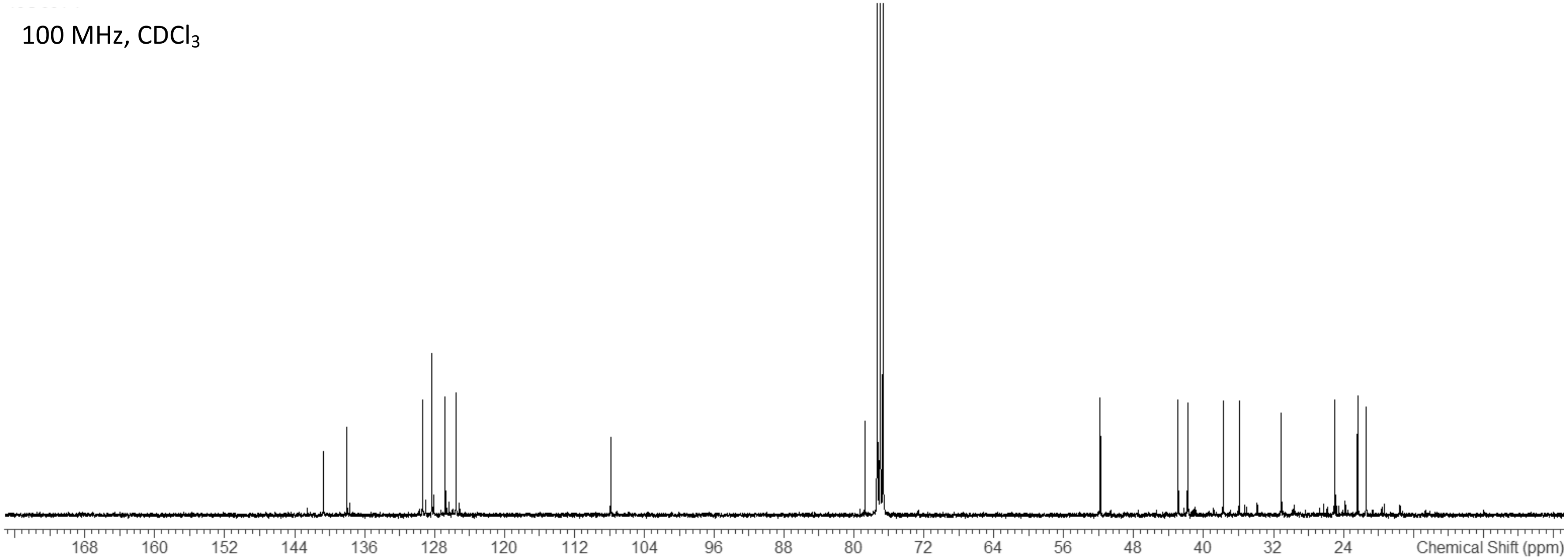

400 MHz, $CDCl_3$

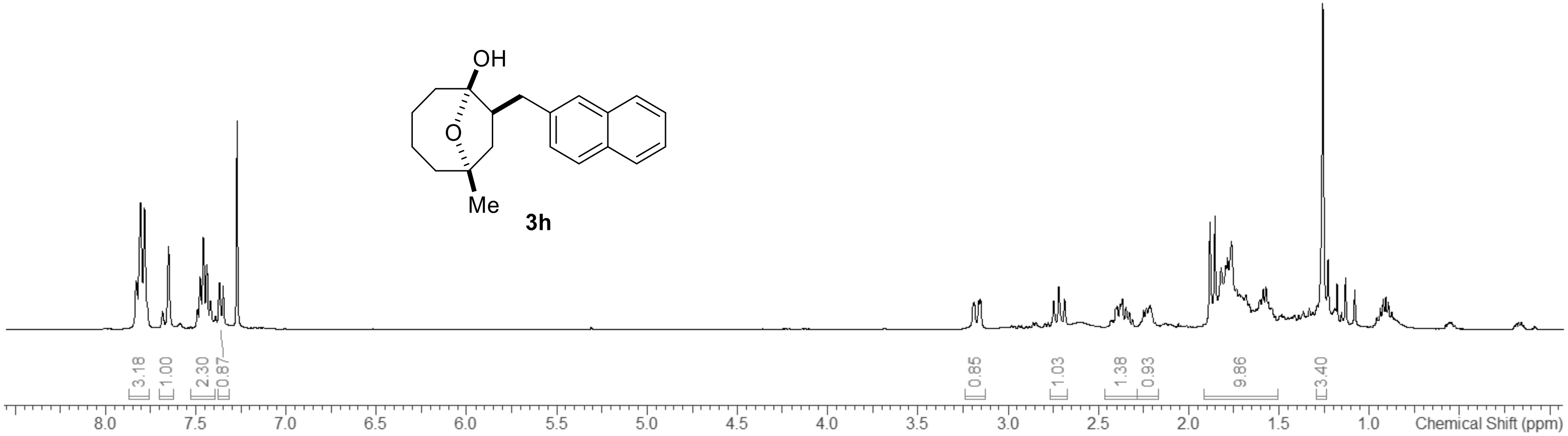


100 MHz, $CDCl_3$

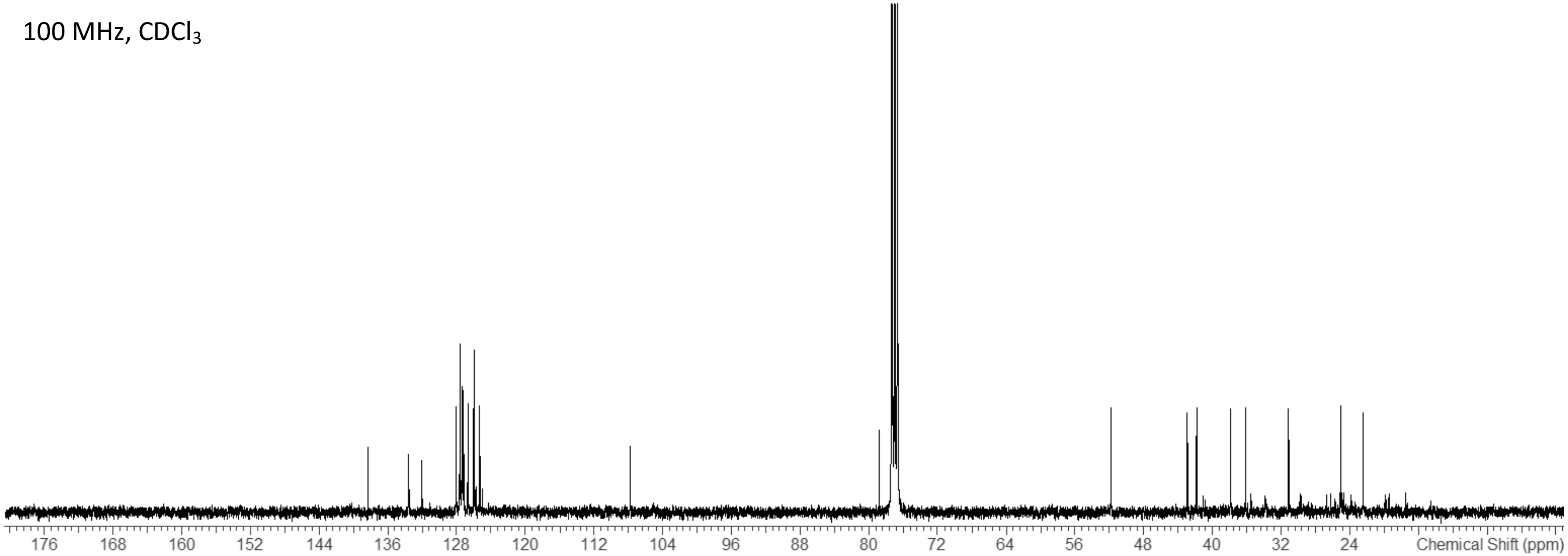

400 MHz, CDCl$_3$

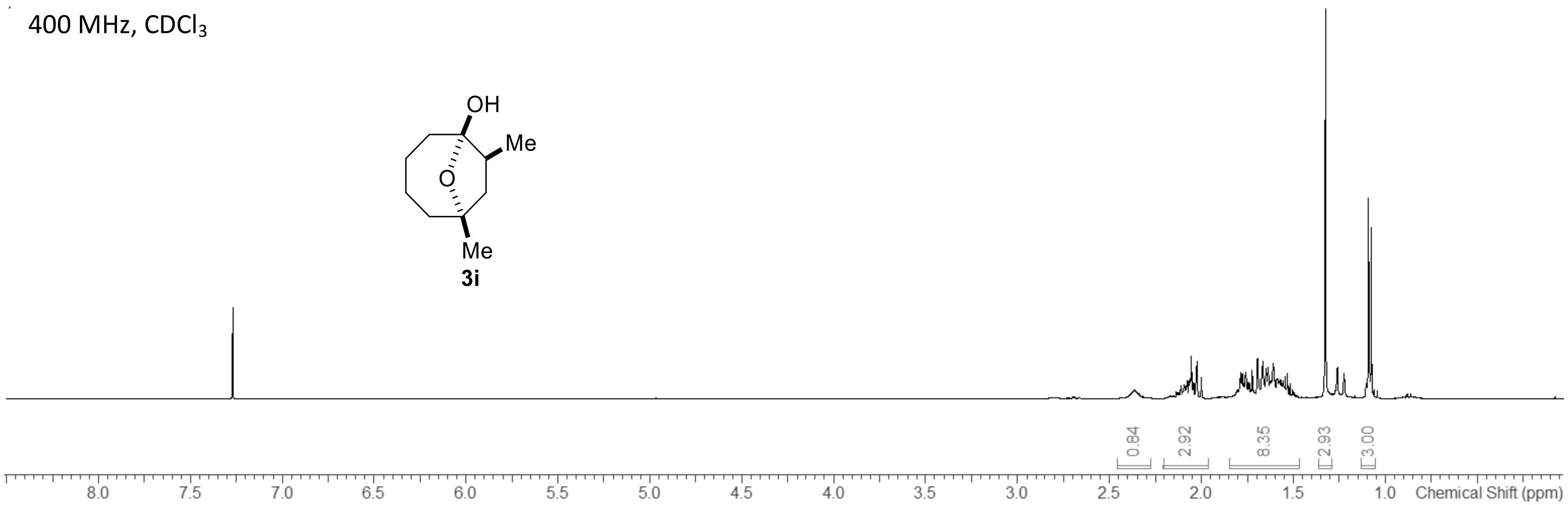


100 MHz, CDCl$_3$

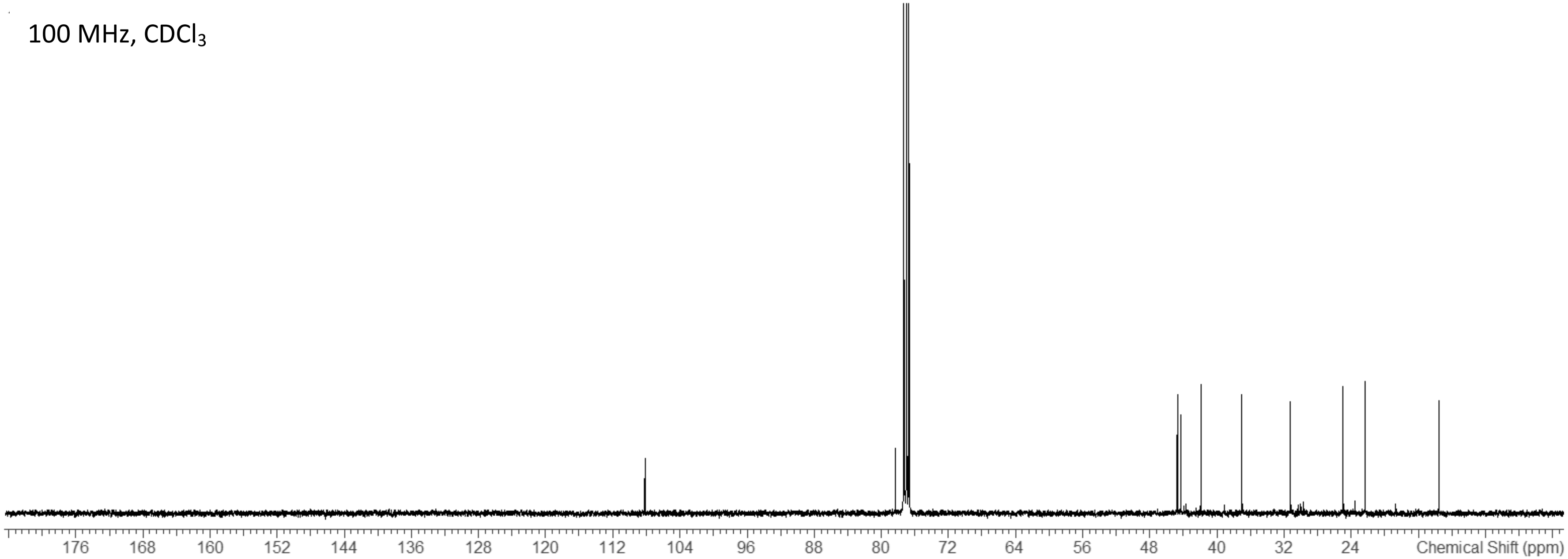

400 MHz, CDCl$_3$

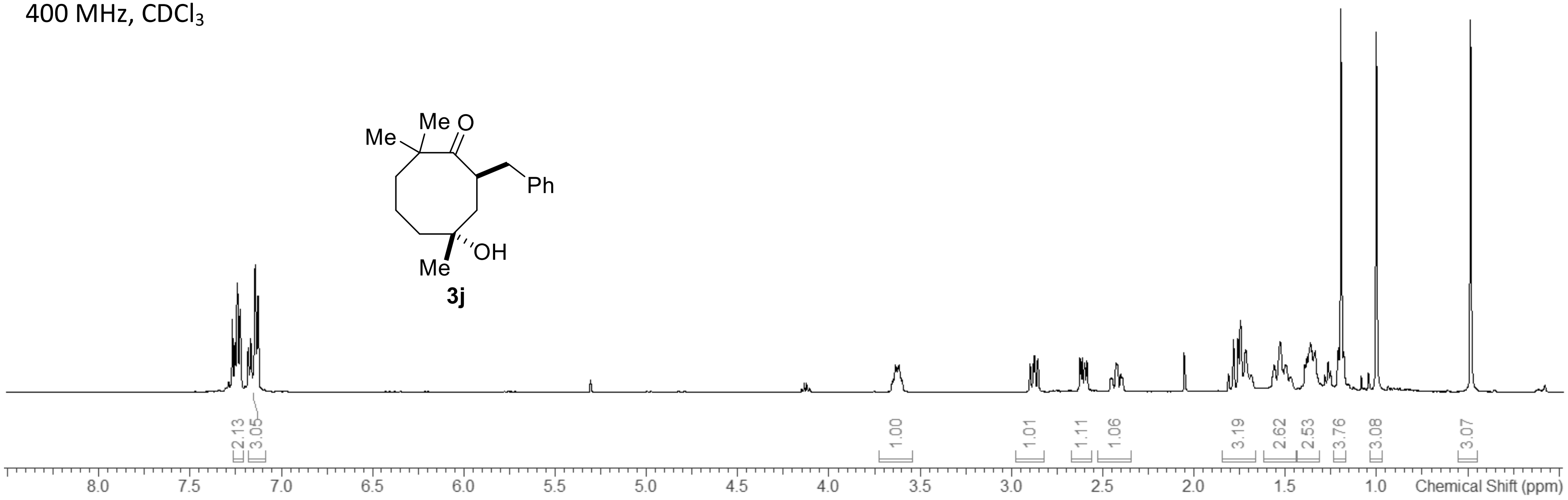


100 MHz, CDCl$_3$

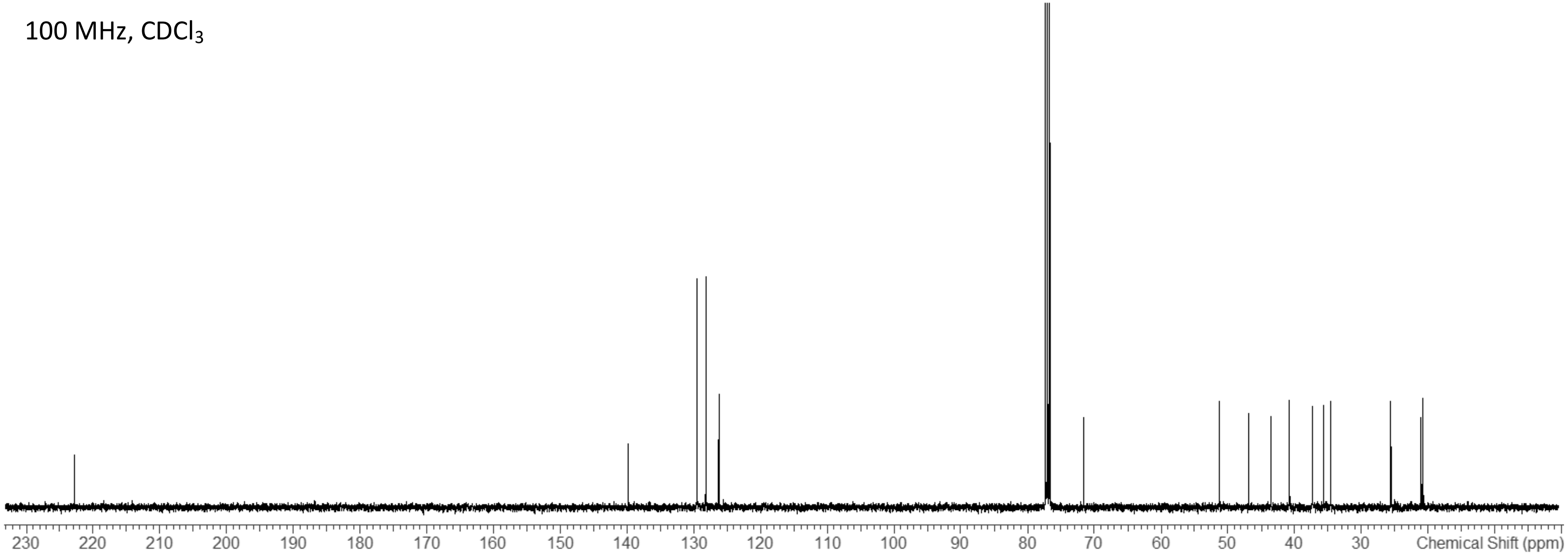

400 MHz, $CDCl_3$

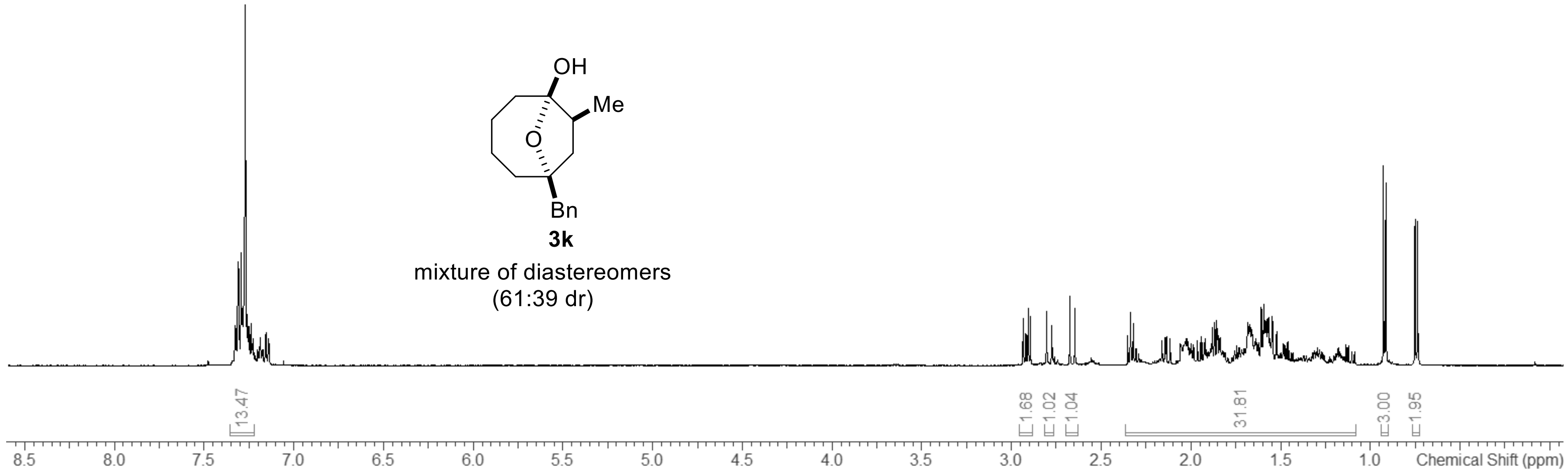


**3k**

mixture of diastereomers
(61:39 dr)

100 MHz, $CDCl_3$

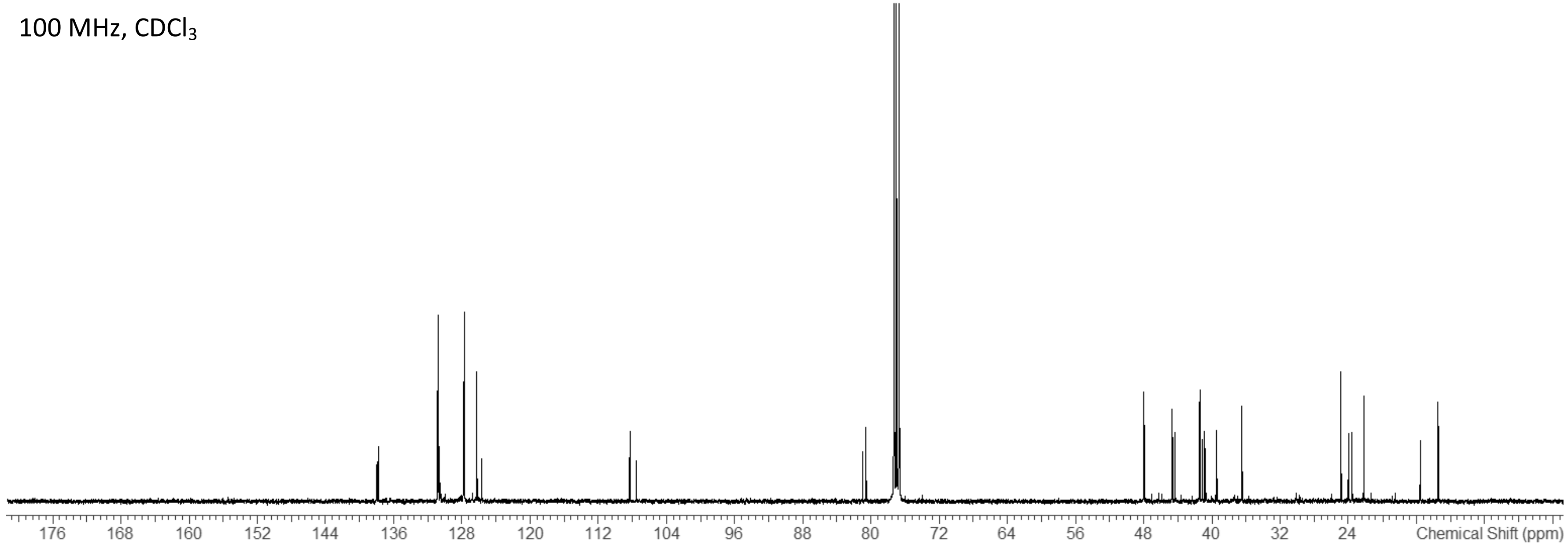

400 MHz, $CDCl_3$

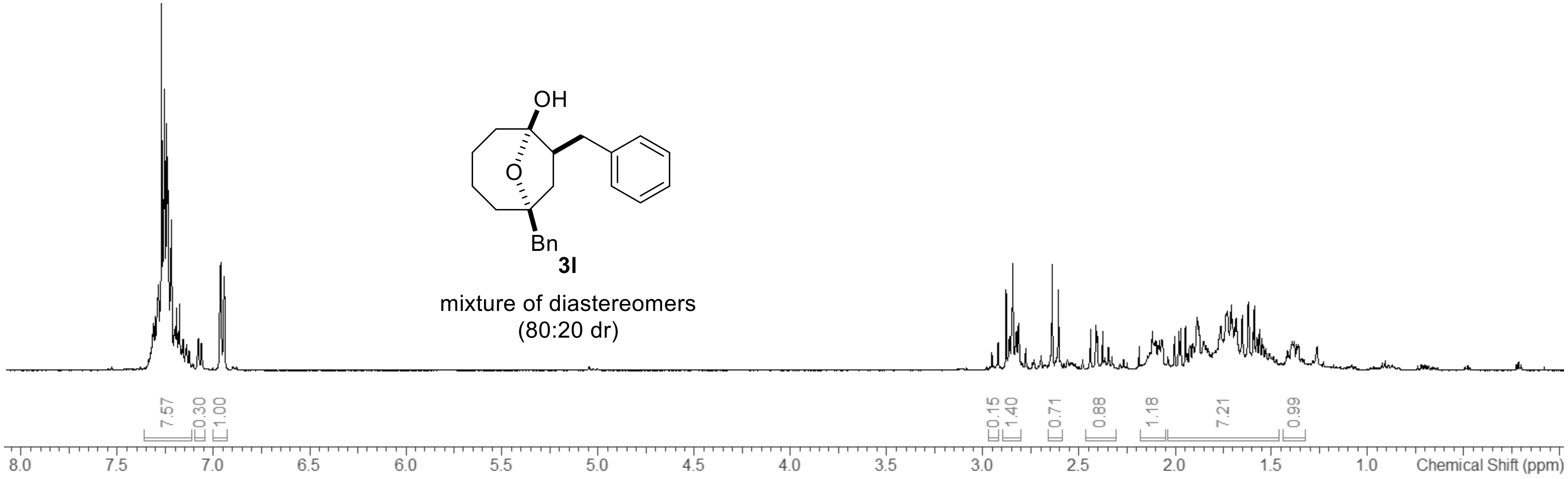

**3l**

mixture of diastereomers
(80:20 dr)

100 MHz, $CDCl_3$

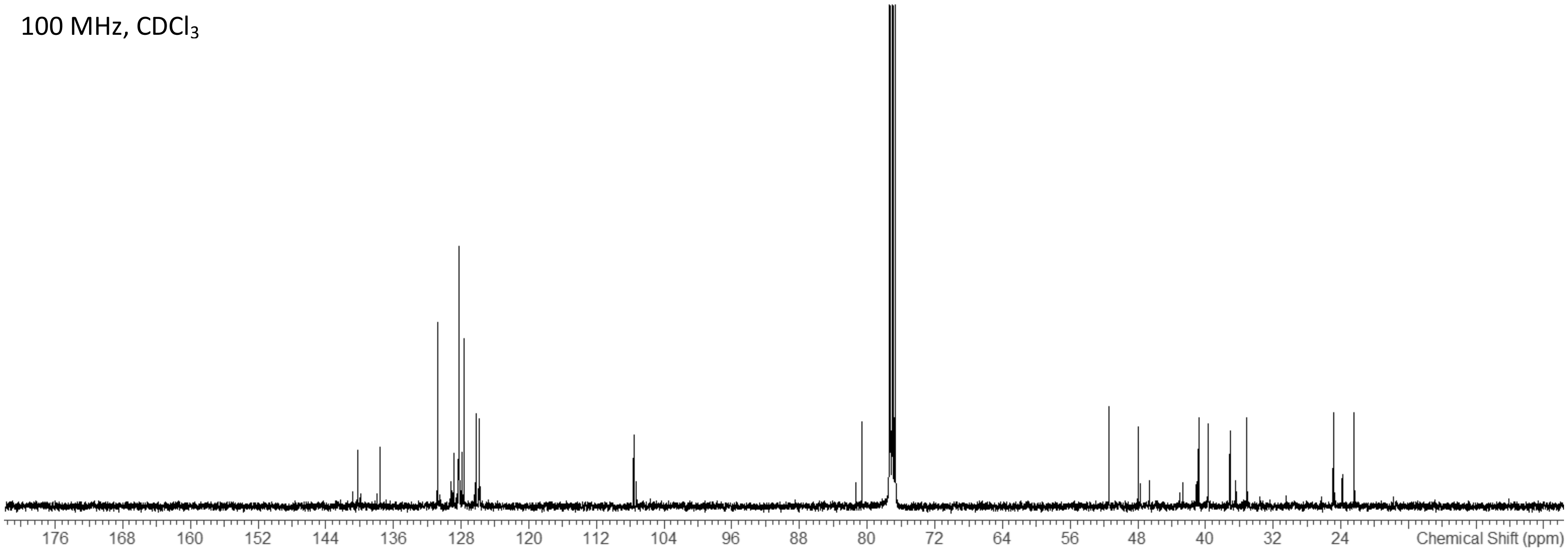

400 MHz, $CDCl_3$

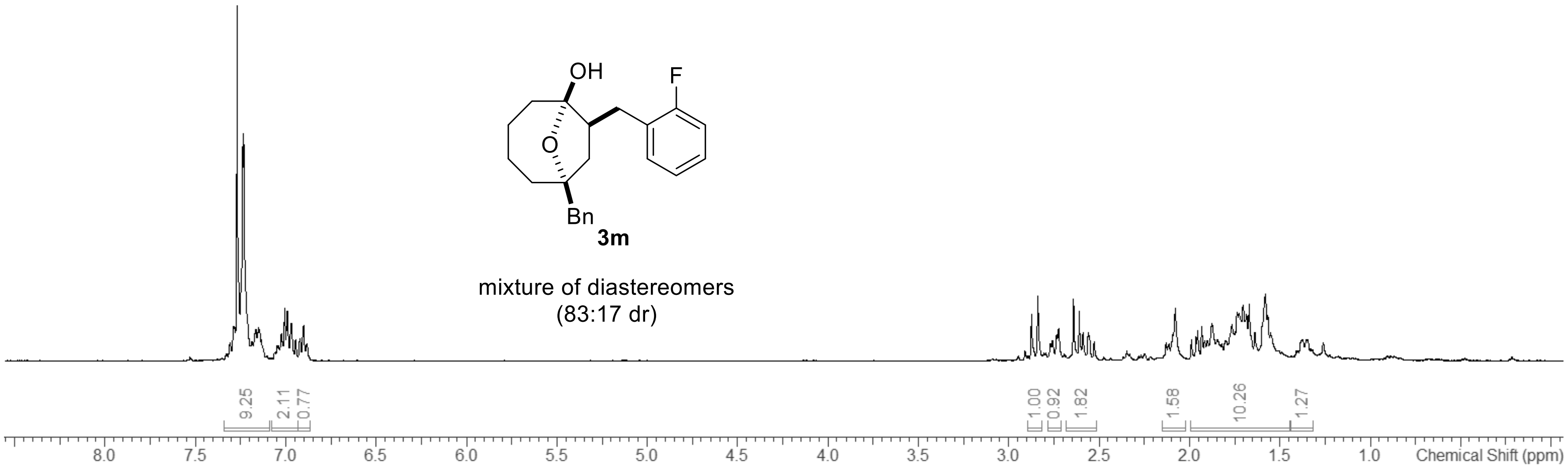


100 MHz, $CDCl_3$

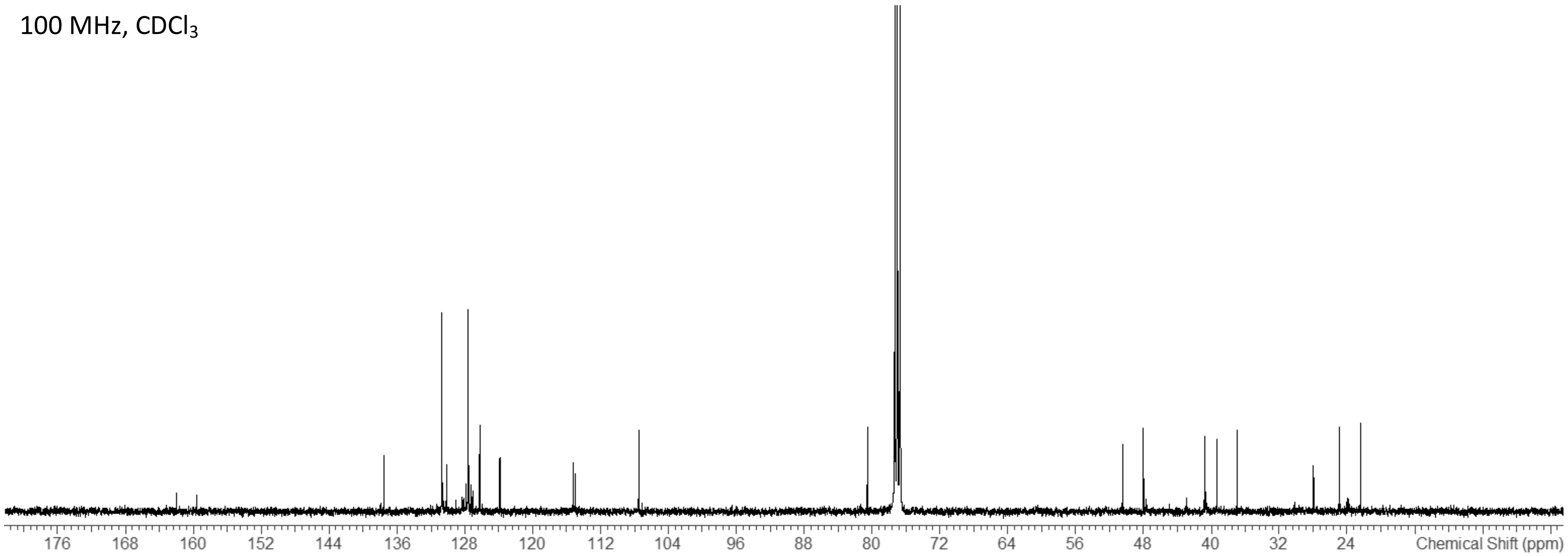

400 MHz, CDCl$_3$

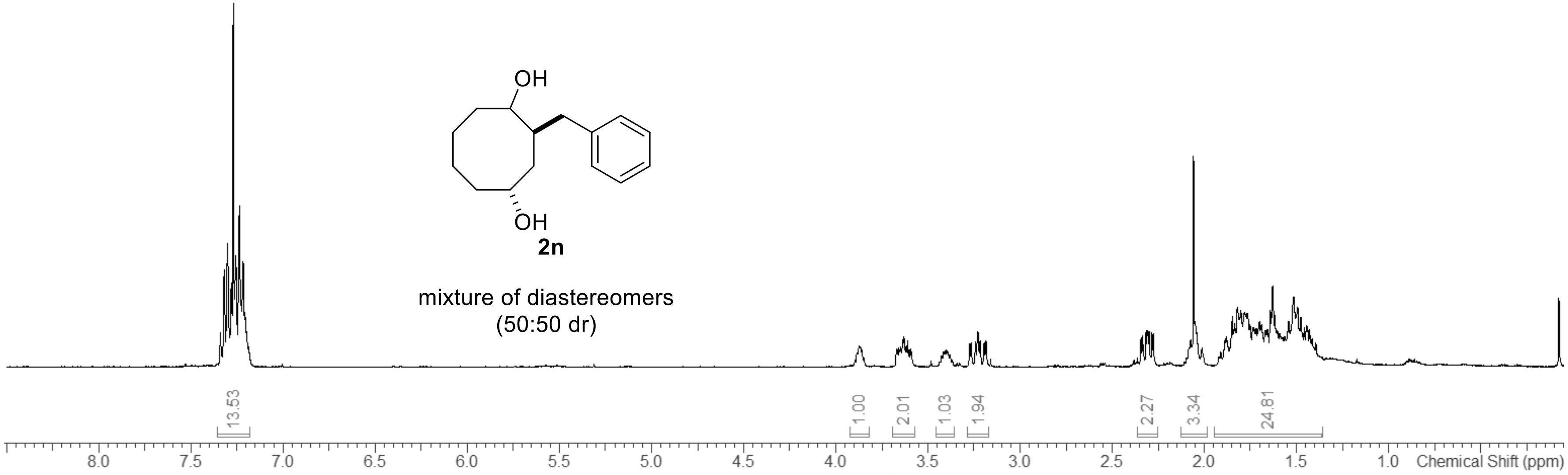


**2n**

mixture of diastereomers
(50:50 dr)

100 MHz, CDCl$_3$

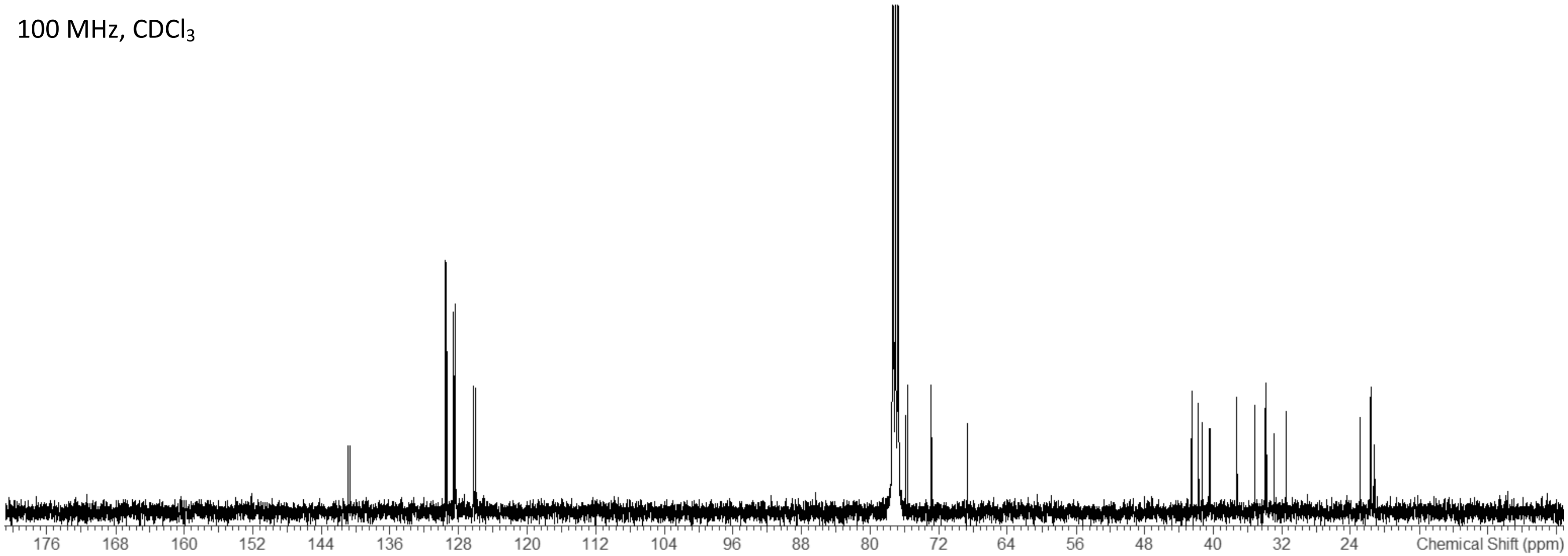

400 MHz, $CDCl_3$

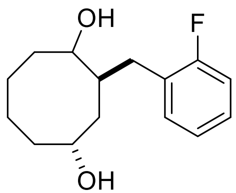


**2o**

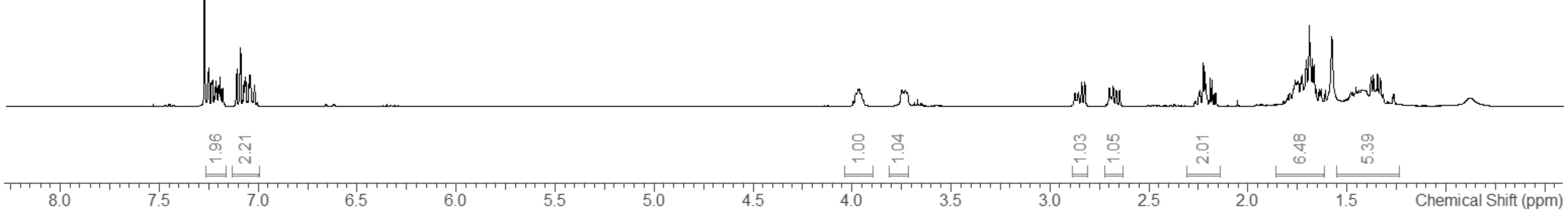


100 MHz, $CDCl_3$

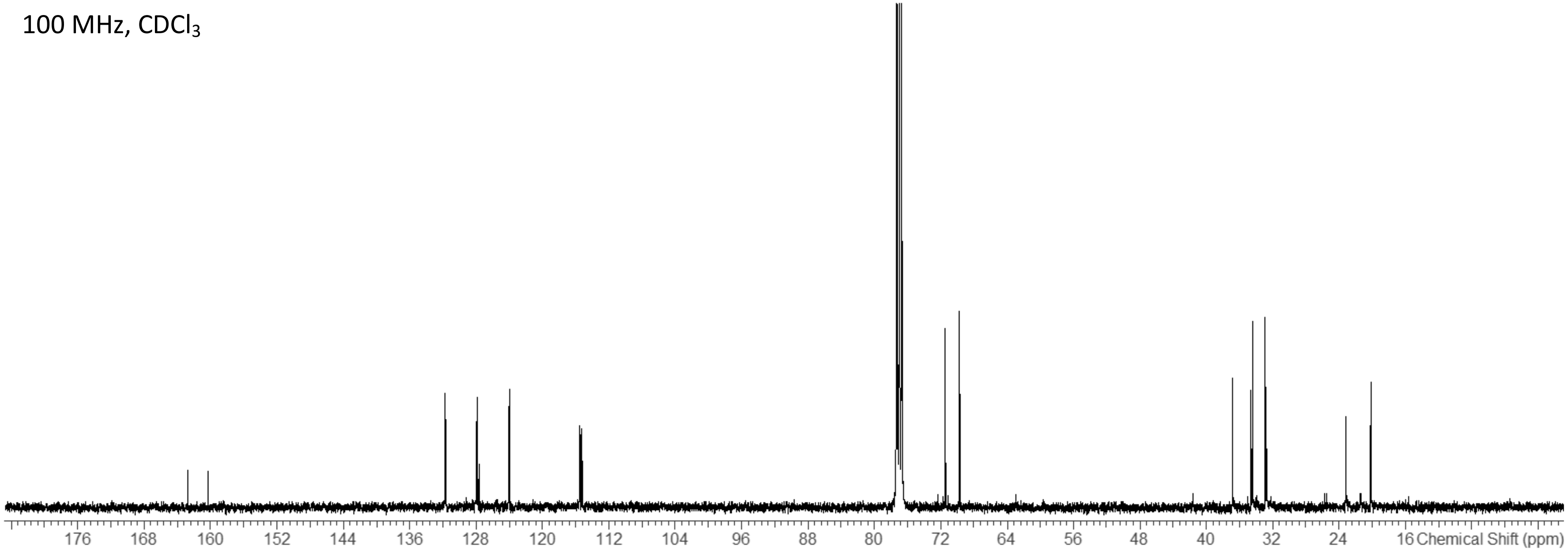

400 MHz, $CDCl_3$

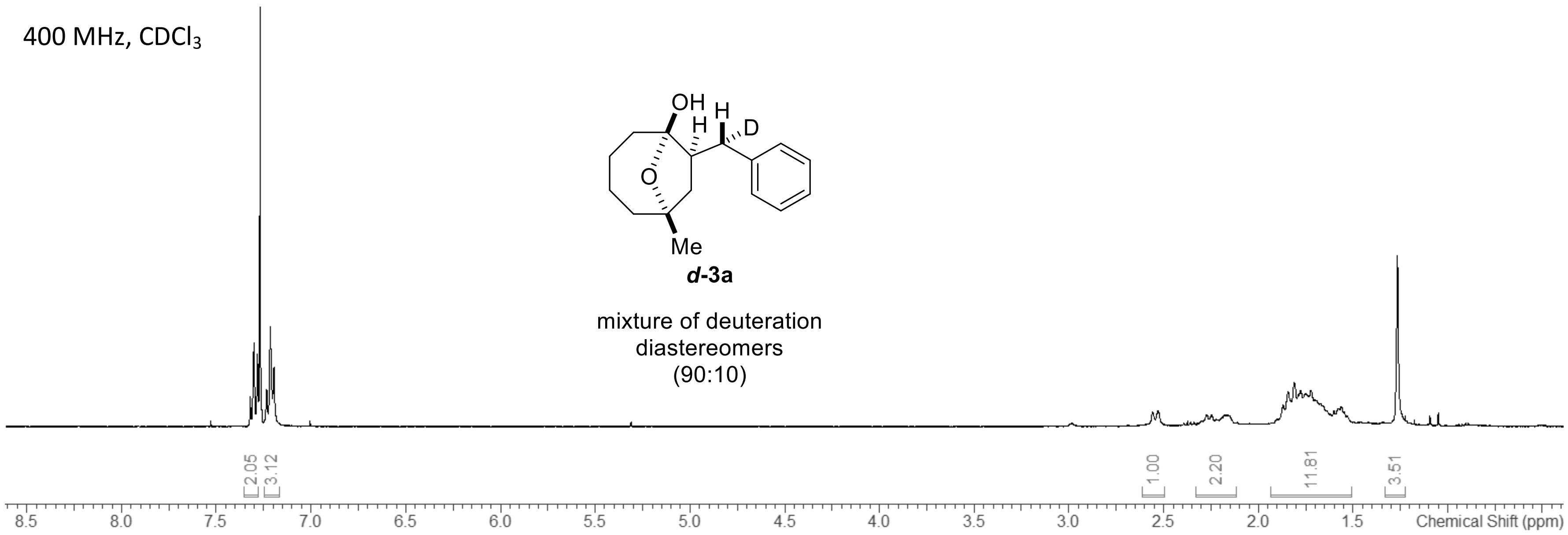


100 MHz, $CDCl_3$

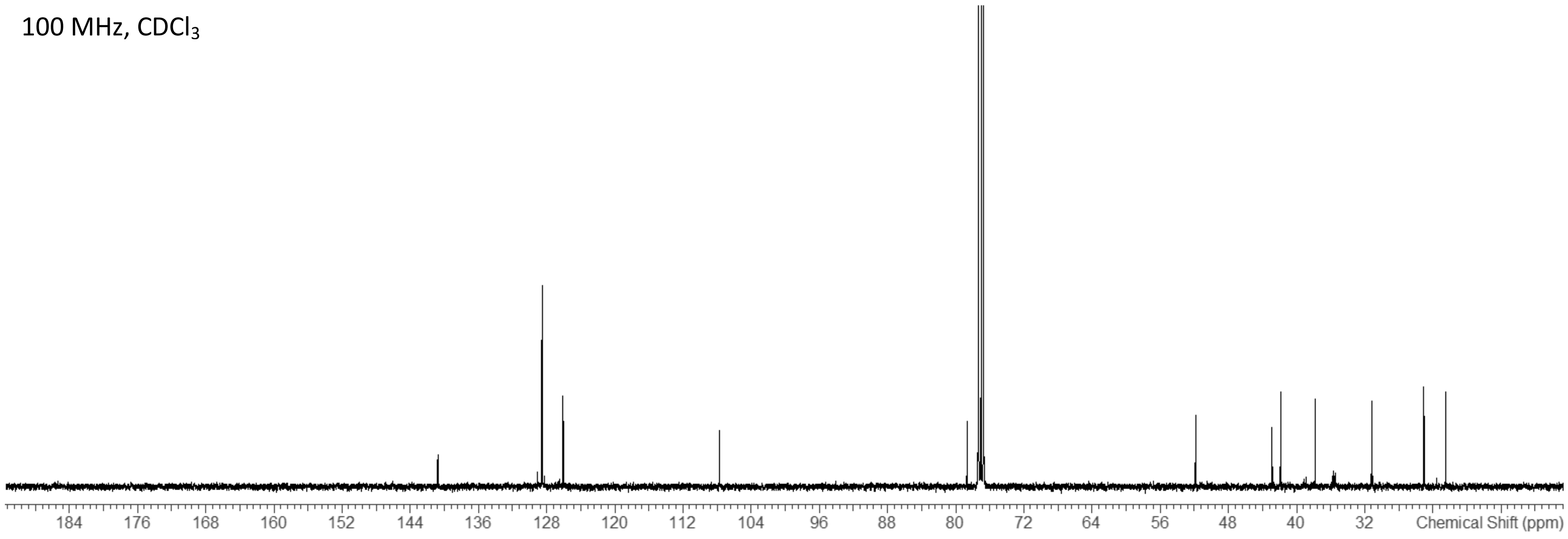

## 10. $D_2O$ experiment

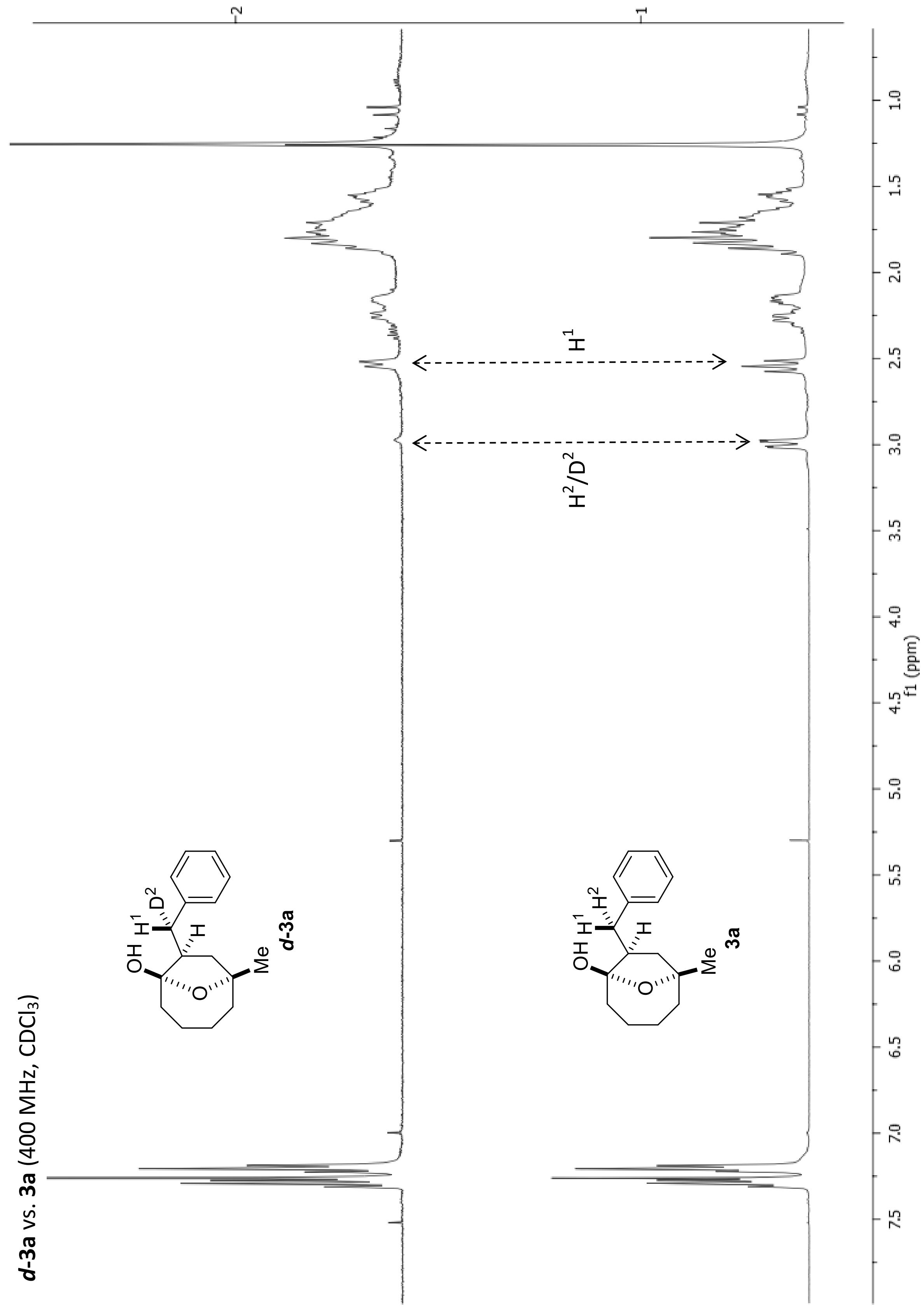

Irradiation of $H^1$ in ***d*-3a major** shows that $H^2$ belongs to ***d*-3a minor**.
(500 Mhz, $CDCl_3$)

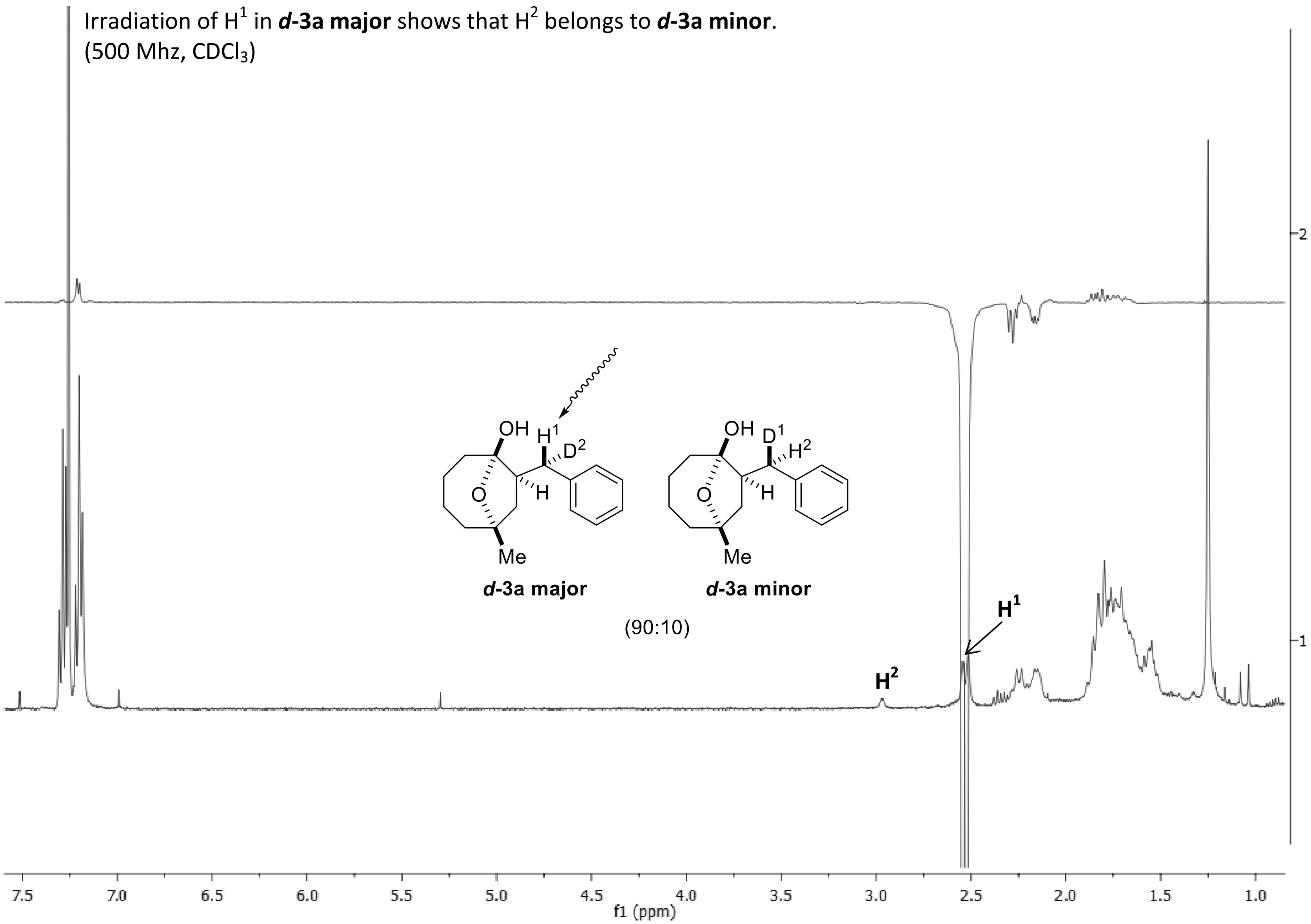

Supplement: Supplementary file 1 — Supplementary [file ANIE-55-12499-s001.pdf]
